# Identification of a pKa-regulating motif stabilizing imidazole-modified double-stranded DNA

**DOI:** 10.1093/nar/gku1306

**Published:** 2014-12-17

**Authors:** Dieter Buyst, Vicky Gheerardijn, Krisztina Fehér, Bjorn Van Gasse, Jos Van Den Begin, José C. Martins, Annemieke Madder

**Affiliations:** 1 Department of Organic and Macromolecular Chemistry, NMR and Structure Analysis Unit, Ghent University, Gent, Oost-Vlaanderen 9000, Belgium; 2 Department of Organic and Macromolecular Chemistry, Organic and Biomimetic Chemistry Research Group, Ghent University, Gent, Oost-Vlaanderen 9000, Belgium

## Abstract

The predictable 3D structure of double-stranded DNA renders it ideally suited as a template for the bottom-up design of functionalized nucleic acid-based active sites. We here explore the use of a 14mer DNA duplex as a scaffold for the precise and predictable positioning of catalytic functionalities. Given the ubiquitous participation of the histidine-based imidazole group in protein recognition and catalysis events, single histidine-like modified duplexes were investigated. Tethering histamine to the C5 of the thymine base via an amide bond, allows the flexible positioning of the imidazole function in the major groove. The mutual interactions between the imidazole and the duplex and its influence on the imidazolium pKa_H_ are investigated by placing a single modified thymine at four different positions in the center of the 14mer double helix. Using NMR and unrestrained molecular dynamics, a structural motif involving the formation of a hydrogen bond between the imidazole and the Hoogsteen side of the guanine bases of two neighboring GC base pairs is established. The motif contributes to a stabilization against thermal melting of 6°C and is key in modulating the pKa_H_ of the imidazolium group. The general features, prerequisites and generic character of the new pKa_H_-regulating motif are described.

## INTRODUCTION

The imidazole moiety contributed by histidine residues is a ubiquitous functionality in proteins and peptides and is known to be involved in many binding and catalysis events. Prominent examples of catalytic sites where imidazole(s) occupies a central role in the mechanism of action include serine proteases, such as α-chymotrypsin (1–3), and ribonucleases, such as RNase A (4–6). With a pKa_H_ value around physiological pH that can be additionally fine-tuned through specific interactions, both the charged (protonated ImH^+^) and the neutral (Im) state can occur in natural systems conferring a versatile general acid/general base character whenever required for binding or catalysis. Not surprisingly, therefore, considerable interest exists in the design of artificial enzymes featuring one or multiple imidazole moieties (7–10). Since this requires a precise organization of the catalytically active moieties, considerable effort has been invested in the *de novo* design of artificial, protein-based enzymes, wherein the engineering of a precise structural organization remains a formidable challenge.

While double-stranded DNA lacks the portfolio of protein side chain functionalities, it features a well-defined and mostly predictable 3D structure. While this should simplify the design efforts, the potential of nucleic acids as potential binders or catalysts has only recently been recognized. A series of successful examples can be found in the work of Roelfes *et al.* (11) where ligands intercalating in a double helix are used to carry out asymmetric transformations using mostly unmodified oligonucleotides. Furthermore, the SELEX (Systematic Evolution of Ligands by EXponential enrichment) technology (12–15) has revolutionized the field of nucleic acid-based binders or so-called aptamers. However, the innate absence of functional side chains in natural nucleic acids and the corresponding nucleotides poses clear limitations in the diversity of binders that can be obtained compared to protein-based ones. Examples exist that circumvent this issue through the introduction of modified residues thereby yielding new non-natural binders (16–19) and catalysts (20–24). However, the SELEX protocol becomes more labor intensive and reproducibility of results is often a problem in these cases. Moreover, no control is possible in terms of positioning of the desired extra functionalities.

**Figure 1.**
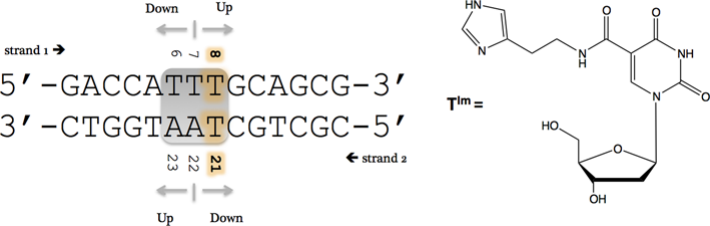
Sequence of the wt (unmodified) DNA template. ‘Up’ follows increasing sequence numbers in the 5‘ to 3‘ direction and ‘down’ is used in the 3‘ to 5‘ direction, with respect to a specific position in the strand. The sites where T^Im^ is introduced and the T-T mismatch are highlighted.

As opposed to the SELEX-based *top-down* approach where a rather random and combinatorial method is applied in the search for enhanced nucleic acid-based binders and catalysts, we became interested in a bottom-up approach for the rational introduction of relevant protein-like functionalities in nucleic acid-based systems. This can be enabled by techniques, such as solid phase synthesis, post-synthetic modifications or enzymatic incorporation of modified analogs (25,26). Here, the systematic introduction via solid phase synthesis of a single imidazole moiety in a duplex DNA structure is presented using T^Im^, a thymine nucleoside bearing an imidazole at C5 (Figure 1).

Because of the stable and predictable Watson–Crick base pairing and stacking interactions, oligonucleotide duplexes allow the design of functional sites in one of the grooves through the carefully planned introduction of functional side chains onto the nucleoside building block. However, one must also consider the impact on the duplex integrity, stability and local structure induced by the modification. A complete picture therefore requires a detailed structural analysis and verification of the modified duplexes in order to validate the design approach. Here, we present a short sequence motif capable of regulating the pKa_H_ of an imidazole functionality tethered to thymine using a short but flexible linker. Using nuclear magnetic resonance (NMR) spectroscopy and unrestrained molecular dynamics (MD), the influence of an incorporated imidazole moiety on duplex stability and structure are delineated, while reciprocally, the impact of the DNA context on the imidazole pKa_H_ through specific interactions involving base pairs in the major groove are presented. The here described pKa_H_-regulating motif, it's portability across other sequences and the procedures used to extract these findings can be of interest for their application in nucleic acid-based binders with enhanced affinities, artificial ribonucleases and potentially for oligonucleotide-based artificial catalysts.

## MATERIALS AND METHODS

### Synthesis of T^Im^-modified DNA

The synthesis of the imidazole-modified nucleoside from thymidine is achieved in three steps as described by Holmes and Gait (27). In a first step, a palladium-catalyzed one-pot carboxamidation reaction is used to attach histamine (Sigma-Aldrich) to 5-iodo-2‘-deoxyuridine (Sigma-Aldrich) via an amide bond. Subsequent *t*Boc-protection of the imidazole N-H and DMTr-protection of the hydroxyl group on the 5‘-position completes the synthesis of a T^Im^ nucleotide building block suitable for incorporation into a DNA sequence via phosphoramidite chemistry using standard protocols (28). Resonance assignment of the imino protons in the T_8_-T_21_ mismatch was achieved by incorporating 5–10% uniformly ^15^N-labeled thymidine (CIL/Buchem) at T_8_ in the unmodified duplex, and opposite T^Im^ in the modified duplexes (29,30). All non-modified, non-labeled strands were purchased from Integrated DNA technologies (IDT, Belgium). Both purchased and synthesized oligonucleotide strands were purified using an ion exchange method (31) in which the oligonucleotides were dissolved in a 95/5 mixture of isopropanol and a 3M NaOAc solution. The mixture was centrifuged (10 min, 7000 revolutions per minute (rpm)) and the oligonucleotide precipitate was resuspended in pure isopropanol and subsequently freeze-dried. The molar quantities of single strands were determined spectrophotometrically by measuring the absorbance at room temperature at 260 nm using a *ϵ* = 99600 M^−1^cm^−1^ (Trinean DropSense 96). The desired double-stranded DNA was obtained from annealing equimolar amounts of the complimentary single strands by heating to 95°C for 30 min, followed by gradual cool-down to room temperature. The annealed duplex sample was dialyzed extensively against H_2_O, using a dialysis membrane with a 3.5 kDa molecular weight cut-off.

### Ultraviolet (UV) thermal denaturation experiments

UV measurements for the determination of the Tm values were performed on a Varian Cary 300 Bio UV/VIS spectrophotometer equipped with a six-cell thermostatted cell holder and N_2_-supply. Melting curves were monitored at 260 nm from 5°C to 90°C with a heating/cooling rate of 0.3°C/min. Three consecutive runs were performed for each duplex, and values represent averages with standard deviations of these runs. The buffer solution contained 100 mM NaCl and 10 mM phosphate (pH 7.0). The oligonucleotide concentration was 1 μM for each strand. The melting temperatures were calculated from the first derivative of the heating curves using the Cary 300 Bio software.

### NMR studies of the modified oligonucleotides

DNA duplexes were dissolved in 550 μl 90/10 H_2_O/D_2_O containing 100 mM NaCl, 0.1 mM EDTA, 0.05 mM NaN_3_ and 0.05 mM DSS (4,4-dimethyl-4-silapentane-1-sulfonic acid) for internal chemical shift referencing. For the assignment of the non-labile protons, the samples were freeze-dried and resuspended in 550 μl of D_2_O sample solution. The pH or pD (corrected for the isotope effect) of the samples was adjusted by adding small aliquots of diluted NaOH and HCl or deuterated equivalents, respectively. Assignment was performed at pH 5.0. All spectra were recorded on a Bruker Avance II spectrometer operating at a ^1^H frequency of 700.13 MHz operating under Topspin 3.1pl6 and using a standard 5 mm inverse TXI-Z ATMA probe head. All measurements for the assignment of the non-labile protons were performed at 25°C (298.15 K), while the 2D NOESY spectra for the assignment of the exchangeable protons were recorded at 5°C (278.15 K). Standard pulse sequences from the Bruker library were used throughout. In D_2_O solution, the residual HDO signal was suppressed using excitation sculpting when necessary (32). All spectra extended over 25.0 ppm (90/10 H_2_O/D_2_O) or 12.0 ppm (D_2_O) along the ^1^H dimension. For the ^1^H 1D spectra (zgesgp), 128 scans of 16K data points were accumulated. Prior to Fourier transformation, the FID's (Free Induction Decay) were zero filled to 32K and were apodized with a squared cosine bell function. Zero-order polynomial baseline corrections were applied. For the COSY (cosydfesgpphpp), TOCSY (clmlevesgpph) and NOESY (noesyesgpph) spectra, 512 t1 increments of 4K data points, 64 scans each, were recorded. Mixing times for the TOCSY and NOESY were 75 and 200 ms, respectively. Apodization with a squared cosine bell function, followed by zero filling and Fourier transformation to a 2K × 2K data matrix, followed by baseline correction yielded the final spectra.

For the spectra recorded in 90:10 H_2_O/D_2_O, the H_2_O signal was suppressed using WATERGATE (33) to account for the larger spectral width (25.00 ppm) 32K data points were collected. For the assignment of imino protons contributed by ^15^N-labeled thymidines, ^15^N-decoupled ^1^H 1D (zggpwgcf3) spectra were recorded and compared to regular spectra to detect the ^15^N-satellites in a 0.5 mM duplex solution. For the assignment of the imino protons, a NOESY spectrum (noesyfpgpphwg) with 80 ms mixing time was recorded, with 512 t1 increments of 4K data points, 64 scans each and processed as described above. The CCPN data model was used for the complete assignment of all duplexes and provides chemical shift data for both exchangeable and non-exchangeable protons (34). This afforded the collection of ^1^H chemical shift perturbations caused by introduction of the imidazolium group and the identification of nOe contacts involving the T^Im^ residue in the duplexes.

### T^Im^ pKa_H_ determination

The pKa_H_ of the imidazole in the T_x_^Im^ was determined by monitoring the pH dependence of one or both of the aromatic proton resonances of the imidazole ring, i.e. Hϵ1 and Hδ2, with 1D ^1^H NMR (35,36). In spite of the considerable ^1^H background signal from the duplex, the lack of other similar functionalities within the DNA structure is exploited to monitor these characteristic resonances. The Hϵ1 resonance occurs mostly downfield from the DNA spectral background, above 8 ppm, while the Hδ2 resonance resides mostly upfield from the aromatic H_6_ and H_8_ protons of the nucleobases. When the imidazole resonances are partially obscured by the duplex background, the mutual long-range ^4^J(Hδ2, Hϵ1) coupling (∼1 Hz) is exploited to generate a characteristic cross-peak in a 2D TOCSY spectrum with 60 ms spinlock time. Since no other protons in this spectral region show any ^1^H -^1^H couplings long range or otherwise, the unambiguous assignment of both the Hϵ1 and Hδ2 protons from this cross-peak is straightforward. As a result, complete *a priori* spectral assignment of the duplex is not necessary for pKa_H_ determination. In all cases, pKa_H_ values were obtained by fitting the chemical shift data obtained between pH values 5 and 10 to a Henderson–Hasselbalch equation. Error values were calculated by using an in-house written Monte Carlo-based algorithm (37). All reported pKa_H_ values were derived from the Hϵ1 chemical shift data since this proton can mostly be observed without any signal overlap. Values obtained, whenever possible, from Hδ2-derived data agreed within error limits with that from Hϵ1.

### Generation of DNA models

Initial non-modified DNA structures for all systems were built using the standard B-DNA parameters in Discovery Studio (Accelrys Software Inc., San Diego, USA, release 4.0). The T^Im^ nucleoside was created starting from thymidine as a template using the builder functionality. Topologies were constructed using the xLEAP interface and AmberTools 12 (38). The parmbsc0 refinement was applied for all nucleic acids, whereas the protonated imidazole ring and subsequent linker parameters were taken from the relevant histidine force field parameters for proteins. The amide bond, attaching the protein-like modification to the thymine base, is described by parameters derived from the AMBER ff12SB force field. Each oligomer was solvated in a truncated octahedral TIP3P water box and placed at least 13 Å (1 Å = 0.1 nm) removed from the edges of the water box. Sodium counter ions were inserted in the water box both for the modified and non-modified DNA structures (25 respectively 26) in order to ensure charge neutrality of the complete system.

### Molecular simulations

All unrestrained MD simulations started following a short standard equilibration protocol that consisted of submitting the initial DNA structures to a two-step energy minimization, first with harmonic restraints (500 kcal.mol^−1^.Å^−2^) on all nucleic acid atoms, then without restraints. Each system was subsequently heated to 300K during a 20 ps NVT run, with weak restraints (10 kcal.mol^−1^.Å^−2^) on the solute. Next, the density of the solvent was allowed to relax during a subsequent 100 ps run, using constant pressure and without imposing any restraints on the DNA structure. Finally, a 50 ns production MD was performed using the NPT ensemble, with snapshots collected every 2 ps.

All MD simulations were run on a single Nvidia GTX680 GPU using the GPU implementation of pmemd provided in the AMBER12 simulation package (39,40). Each simulation was performed using periodic boundary conditions and a 15 Å cut-off for the particle mesh Ewald summations (41) of non-bonding interactions. The SHAKE algorithm (42) was applied to bonds involving hydrogen atoms, allowing a 2 fs time step. Temperature was regulated using Langevin dynamics (43) with a collision frequency of 1.0 ps^−1^. Finally, pressure regulation was obtained using isotropic position scaling with a pressure relaxation time of 2.0 ps. Using this setup, the complete calculation took about 96 h for each DNA duplex.

The calculated trajectories were visualized using VMD 1.9.1 (44) while further in-depth analysis was performed using 3DNA (45) for the structural and conformational DNA parameters and the HBonanza (46) python script for hydrogen bond analysis. Further data post-processing and analysis utilized various VMD plugins complemented with custom-made MATLAB scripts.

Persistence and quality of base pair hydrogen bond formation, the extent of the α/γ conformational space sampled and the torsional parameters (ϵ-ζ) indicative of B_I_/B_II_ conformations were all monitored (see Supplementary Figures S14–S17) for the non-modified wild-type (wt)-duplex and all T_x_^Im^-modified systems (47–49). In addition, the integrity of the B-DNA duplex form during simulation was checked and found to be respected (see Supplementary Figures S18 and S19).

## RESULTS AND DISCUSSION

### Introducing an imidazole function into the major groove

Equipping DNA duplexes with additional functionalities can be most simply achieved by the introduction of custom-made nucleosides through modification at the level of sugar, phosphate backbone or base. Introduction of functionalities at C5 of pyrimidine bases positions the newly introduced moieties in the chiral and hydrophobic microenvironment of the major groove where sufficient space is available for accommodation of multiple functionalities and/or binding of potential substrate molecules. Here, histamine is tethered to the C5 of thymidine via an amide bond, placing the imidazole function in the major groove. Only minimal disruption of the helical structure is expected, in contrast with, for instance, modifications at the C2′ of the ribose sugar (50). This is validated from the MD results described further.

### Selection of the DNA template sequence

The sequence of the DNA template used throughout this work was selected based on three criteria. First, insertion of a modified nucleotide can reduce the overall duplex stability. Therefore, the sequence should be of adequate length and composition to offset such effects. Second, the ideal template should have sufficient AT base pairs distributed throughout its sequence in order to assess the impact of the modification's location on the properties of the duplex. In this respect, varying the T^Im^ location on the same template allows for comparative analysis. Furthermore, a judicious distribution should allow to envisage a variety of catalytic configurations when exploring multiple T^Im^-modified duplexes. Third, choices regarding the sequence length and composition should aim to facilitate spectral interpretation by NMR, e.g. by avoiding local and global symmetries. Also, sequences of considerable length will have a negative impact on synthesis efficiency, further compromising NMR analysis due to limited sample availability.

Taking these considerations into account, the sequence shown in Figure 1 was chosen. As fraying destabilizes base pairs at the duplex ends and a full turn of B–DNA requires about 11 base pairs, a 14 base pair duplex was used as template. To limit the extent of fraying, the duplex was prolonged at each end with one and two GC base pairs, respectively.

To meet the design criteria, a variety of DNA sequences were tested for their overall stability using UV measured melting temperature values (Tm). In addition, inspection of the number and line-width of resonances in the imino region of ^1^H NMR spectra (5–65°C, see Supplementary Figure S4) was used to establish individual base-pair stability in a qualitative manner, together with overall spectral dispersion and appearance of the ^1^H NMR spectrum. The final duplex sequence, hereafter referred to as the wt-duplex shown in Figure 1, represents a trade-off with respect to duplex stability and spectral simplicity, while maintaining sufficient modification points to study the impact of a single modification on the properties of the imidazole function and the duplex template. Since ultimately two (or more) T^Im^ modified building blocks are to be introduced, the location of AT base pairs was also optimized to allow variation in mutual spatial location. With the introduction of multiple T^Im^ building blocks at the same level in the DNA sequence in mind, a T_8_ –T_21_ mismatch was introduced at the center of the sequence. At 48.6°C, the Tm value drops by ∼10°C compared to the full Watson–Crick paired duplexes A_8_T_21_ and T_8_A_21_ (Table 1 (51,52)). However, it enables future investigations of double modification at the same base pair level. As an added bonus, the central mismatch leads to an increase in spectral resolution beneficial for the NMR assignments.

**Table 1.** Overview of melting temperatures and pKa_H_ values for all systems studied

	T^Im^	T-T mismatch				
System		WT	T_6_^ImH+^	T_7_^ImH+^	T_21_^ImH+^	T_8_^ImH+^
Tm(°C)*^a^*	n.r.*^c^*	48.6 ± 0.5	50.5 ± 0.2	50.1 ± 0.3	53.3 ± 0.3	54.6 ± 0.1
pKa_H_*^b^*	7.17 ± 0.02	/	8.13 ± 0.04	7.92 ± 0.04	7.70 ± 0.02	8.88 ± 0.05
			
	**TA**		**AT**		**AT scrambled**	
**System**	**T_8_A_21_**	**T_8_^ImH+^A_21_**	**A_8_T_21_**	**A_8_T_21_^ImH+^**	**sT_21_**	**sT_21_^ImH+^**
Tm(°C)	58.9 ± 0.2	64.1 ± 0.6	57.2 ± 0.2	59.3 ± 0.5	53.2 ± 0.3	59.4 ± 0.2
pKa_H_	/	8.72 ± 0.02	/	7.62 ± 0.03	/	9.07 ± 0.02

*Notes:^a^*Tm values were determined in 100 mM NaCl, 10 mM phosphate buffer, 1 μM duplex at pH = 7.0.

*^b^*pKa_H_-values for the imidazolium group were established using NMR monitoring of selected resonances, as explained in the text.

*^c^*n.r. not relevant, since T^Im^ represents the building block itself rather than a DNA duplex and therefore there is no melting process. In addition, see also Supplementary Table S1.

Initially, four modified duplexes featuring one T^Im^ were investigated and their properties compared to the wt-duplex at pH 7.0. They are referred to as the T_6_^Im^, T_7_^Im^, T_8_^Im^ and T_21_^Im^ duplexes, indicating the incorporation of the imidazole functionalized thymidine at positions 6, 7, 8 and 21 (Figure 1). T_x_^Im^ will be used when simultaneously referring to several of these modified duplexes.

### The introduction of T^Im^ does not destabilize the DNA duplex

The Tm values measured by UV for all T_x_^Im^ at pH 7.0 (Table 1) demonstrate that none of the modified systems are destabilized by the introduction of the imidazole-tethered thymidine. A modest increase is found for the T_6_^Im^ (+1.9°C) and T_7_^Im^ (+1.5°C) duplexes compared to the wt-duplex. In contrast, introducing T^Im^ at the level of the T-T mismatch results in a significant increase in Tm of 6°C for the T_8_^Im^ and 4.7°C for T_21_^Im^ duplexes. This implies that the overall stability of the modified DNA duplex is enhanced to a varying degree by a contribution of the modification and that part of the loss in duplex stability resulting from the presence of the mismatch is undone when introducing one T^Im^ at this position. More insight into the latter follows from comparing fully Watson–Crick paired unmodified and modified AT^Im^ duplexes, all lacking the T-T mismatch. In the case of A_8_T_21_^Im^, stabilization is reduced from 4.7°C to 1.9°C, a value similar to T_6_^Im^ and T_7_^Im^ duplexes. This supports a view where introduction of T^Im^ at position 21 in the mismatched sequence partially compensates the destabilizing effect caused by the mismatch at this position. For T_8_^Im^A_21_, however, stabilization remains high at 5.2°C compared to 6°C in the original mismatched T_8_^Im^ duplex (Table 1). The fact that stabilization is maintained irrespective of the presence of a mismatch in the duplex sequence points to a fundamentally different origin in T_8_^Im^ compared to all other T_x_^Im^ sequences.

### pKa_H_ study of T^Im^-modified duplexes

**Figure 2.**
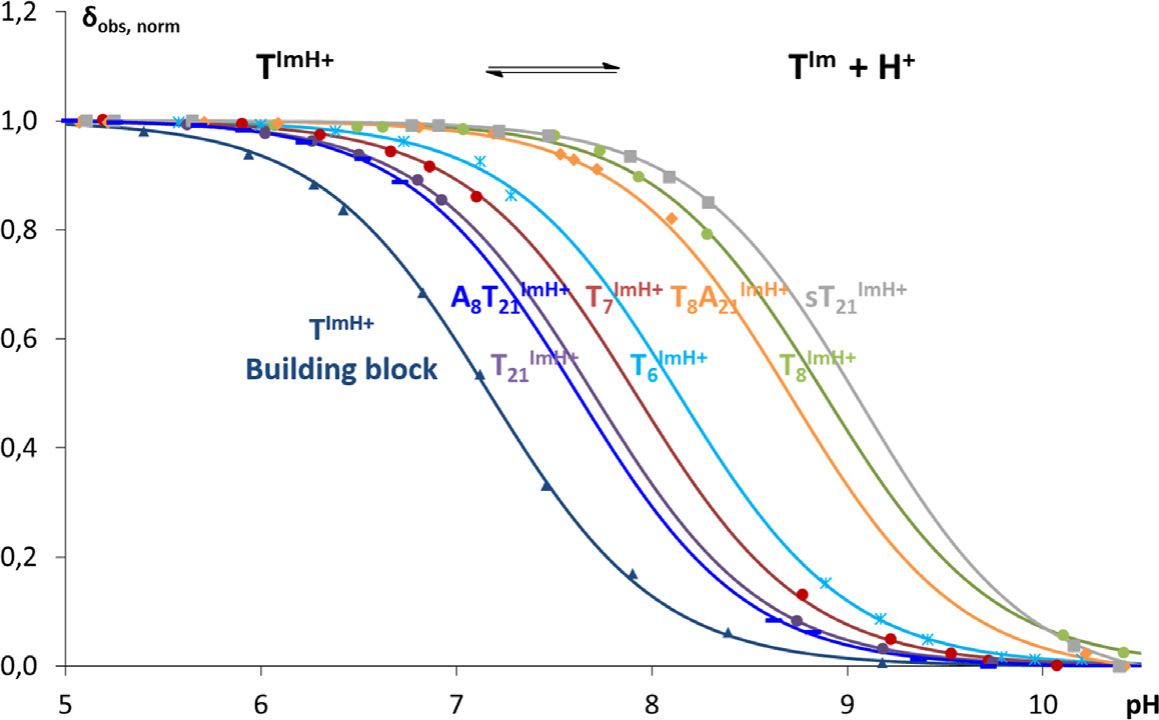
Normalized observed chemical shifts as a function of pH for the isolated building block as well as for all studied T_x_^Im^ duplexes. Both the experimental data and fitted curves are shown. T^Im^ and T^ImH+^ refers to the neutral imidazole protonated and charged imidazolium state of the function, respectively.

The stabilization apparent in T_x_^Im^ duplexes suggests the possibility of an interaction involving the imidazole function, the tether or both. Given the negative charge of the phosphate backbone, an electrostatic interaction involving ImH^+^ appears reasonable as a stabilizing interaction that should contribute to all systems. If present, a concurrent shift in the deprotonation equilibrium of ImH^+^ should be apparent, leading to a rise in pKa_H_ that may correlate with the trend in melting temperature. Since the large UV background of the DNA structure precludes UV-based pKa_H_ determination, 1D ^1^H NMR was used instead. pH-induced chemical shift changes of the non-exchangeable Hϵ1 and Hδ2 imidazole resonances are followed, as detailed in Materials and Methods. The experimental data and fitted titration curves are presented in Figure 2 while pKa_H_ values are collected in Table 1. At 7.17, the pKa_H_ of the T^Im^ nucleoside falls within the pKa_H_ range reported for imidazole (7.0) and 2- or 4-methylimidazole (7.75 and 7.9) respectively (53,54).

Once incorporated in the duplex sequence, the pKa_H_ value in the T_x_^Im^ systems increases between 0.53 and 1.75 units compared to the T^Im^ nucleoside. All pKa_H_ values show that, at pH 7.0, the imidazole group is protonated for ∼90% or more in all T_x_^Im^ duplexes. Thus, a positively charged imidazolium is present in all cases, capable of contributing stabilizing electrostatic interactions with the negatively charged phosphate backbone. At 8.88 ± 0.18, the pKa_H_ of T_8_^Im^ is 0.9 units higher than the average pKa_H_ value of the other three T_x_^Im^ duplexes (7.92 ± 0.03). This significantly higher pKa_H_ is well maintained (8.72 ± 0.02) in the T_8_^Im^A_21_ duplex, underlining that its higher value is independent from the T-T mismatch at this position. T_21_^Im^, on the other hand, displays the lowest pKa_H_ value, a feature maintained in the A_8_T_21_^Im^ duplex. Thus, introducing T^Im^ on opposing sides of the T-T mismatch leads to a quite different effect on the imidazolium group's acidity. For T_6_^Im^ and T_7_^Im^, the pKa_H_ remains close to that observed for T_21_^Im^. Most notable, however, is the existence of a correlation between the significantly higher pKa_H_ and Tm values irrespective of the mismatch when introducing T^Im^ at position 8. This marked position-dependence suggests a specific interaction somehow tied into the local base pair context. This proposal is further supported by noting that when a T^Im^ is introduced at the same base-pair level but on opposing strands, the space available for the imidazolium group within the major groove must necessarily be different when the integrity of the duplex is maintained.

### nOe-contact mapping and chemical shift perturbation of the T_x_^Im^ duplexes

**Figure 3.**
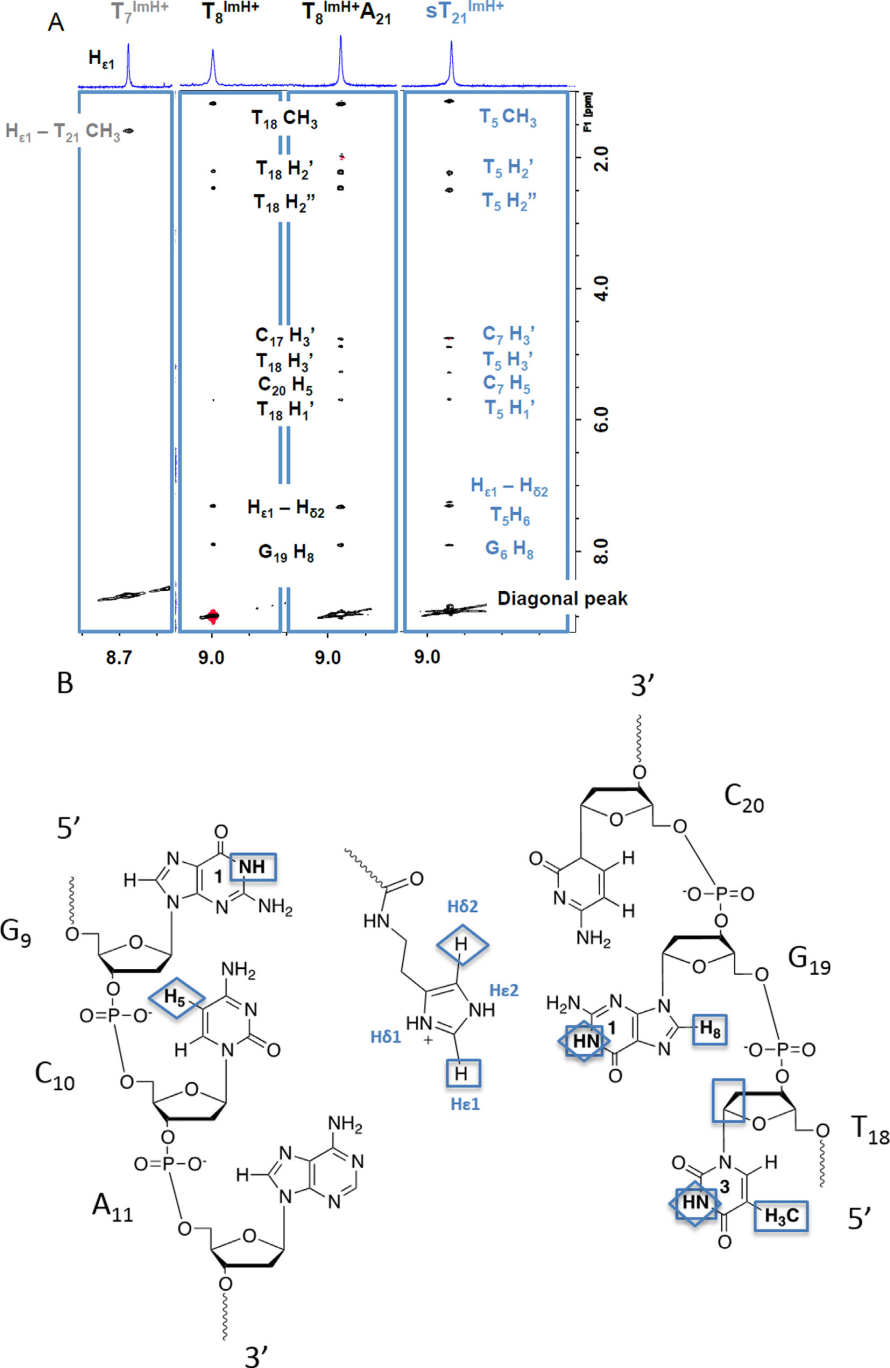
(A) Overview of the specific nOe contacts of the Hϵ1 proton of T_8_^Im^, T_8_^Im^A_21_, sT_21_^Im^ and T_7_^ImH^ to the DNA backbone (2D NOESY, 200 ms, 700 MHz, 25°C, D_2_O). (B) Schematic overview of the specific nOe-contacts from the T_8_^ImH+^ Hϵ1 (□) and Hδ2 (◊) protons to the nucleotides up-stream from the modification site.

The assignment of imino- and non-exchangeable resonances for all T_x_^Im^ was achieved using established methods for unlabeled oligonucleotides (55–57), and was performed at pD/pH 5 to ensure full protonation of the imidazolium group. The NMR spectra, in terms of sequential nOe contacts and intraresidue COSY intensities, are consistent with a right-handed double helix in the B-family of conformations. From these, chemical shift perturbations and nOe contacts involving the modified T^Im^ could be analyzed. In the case of the T_8_^Im^ duplex, clear chemical shift perturbations larger than 0.1 ppm extend up-sequence from the modified base pair to the A_11_-T_18_ base pair, i.e. 3 base pairs from the T_8_ -T_21_ mismatch (see Supplementary Figure S8). A marked up-field chemical shift change of 0.46 ppm is observed for the T_18_ methyl group, indicating a major change in the local environment of the major groove. This is in line with a set of distinct nOe contacts (Figure 3B) that connects the Hϵ1 and Hδ2 protons of the imidazole ring to H1 and H8 of G_19_ and H1 of G_9_ (position +1), H5 of C_10_ (position +2) and H3, H1′, H2′ and H2″ of T_18_ (position +3). These indicate that the imidazolium group extending from the T_8_^Im^ base folds back into the major groove toward the base pairs up-sequence from T_8_^Im^. A long-range nOe to the T_18_ methyl group accompanies the aforementioned up-field shift for the latter. The extent of chemical shift perturbation (Supplementary Figure S8) and cluster of nOe's observed for the T_8_^Im^ duplex contrasts sharply with the T_6_^Im^, T_7_^Im^ and T_21_^Im^ ones, where chemical shift perturbations quickly wane in number and magnitude beyond the immediate neighboring base-pairs. Even more indicative are the nOe's involving the imidazolium that are scarce and very weak. For instance, T_7_^Im^ only displays a nOe contact between Hϵ1 and the methyl of the neighboring T_21_ base (see Figure 3A). Another element differentiating T_8_^Im^ is that it is the only modified duplex where the exchangeable Nϵ2 proton is in slow exchange with the solvent, allowing its tentative assignment at pH 5 and lower temperature (5°C) and the extraction of a number of nearest neighbor nOe contacts (see Supplementary Figure S23). The interactions in the T_6_^Im^, T_7_^Im^ and T_21_^Im^ systems are too transient to allow the visualization and identification of the exchangeable imidazole protons under these conditions. Combined with the melting temperature and pKa_H_ studies, all data available for the T_8_^Im^ system are compatible with a preferred and stable conformation of the imidazolium functionality inside the major groove, placing it in the vicinity of the C_10_G_19_ and A_11_T_18_ base pair steps. The formation of a specific interaction linked to this conformation could not only contribute to an increased thermal stability but also enhance the proton acceptor capacity of the imidazole and hence account for the observed significant increase in pKa_H_ for the T_8_^ImH+^. This hypothesis also implies that systems that lack the increase in stability and pKa_H_ lack this specific interaction, as shown by the dearth of nOe contacts for the other T_x_^Im^ systems.

While distance restraints extracted from nOe data can be used for structure calculations, the sparse nature of the long-range nOe contacts along the linear DNA sequence, combined with the inability to use ^13^C and ^15^N editing to harvest most of the nOe data would undoubtedly prevent to produce an ensemble of sufficient precision and accuracy to investigate the structural details of the modified duplexes, and the nature of the specific interaction in sufficient detail. As an alternative, therefore, we turned to unrestrained MD simulations, which were confronted with the nOe data, to further explore the specific nature of the DNA-imidazolium interactions in the T_x_^Im^ duplexes.

### MD of the wt sequence and general impact of the T^Im^ modification

MD is established as a reliable simulation method to study a broad range of biomolecules including DNA, with simulation times recently extending up to the millisecond timescales (58). Using a protocol similar to that described by Pauwels *et al.* (59), we used MD simulations of all T_x_^Im^ duplexes in explicit solvent to investigate the conformational behavior of the imidazole moiety. Since all NMR data were recorded at pD 5.0, simulations used a charged imidazolium group, which is explicitly referred to as T^ImH+^. To minimize bias, all simulations started with the tether and imidazolium extended outwards from the major groove into the solvent and ran over 50 ns to allow sufficient sampling of conformational space to reveal specific interactions. Subsequently, the trajectories were also confronted with the experimental NMR data to obtain a complete picture.

In order to estimate the impact of the modification on the DNA structure as a whole, a reference simulation of the same time span was performed on the original non-modified wt sequence. This first simulation was also used to evaluate the overall quality of the MD trajectory and stability of the B-DNA type helix during the simulation by extracting a number of parameters known to be good indicators of the simulation quality (*vide supra*, (60)). When all these parameters are considered (Supplementary Figures S14, S16 and S18), the wt trajectory shows no unexpected behavior. The end base pairs show indications of fraying as can be expected, imino hydrogen bonds within the duplex sequence are persistent and the conformational space explored remains within the regions expected for the B-DNA conformational space. A notable exception is the G_9_ base neighboring the T_8_ -T_21_ mismatch, which shows two excursions from the global minimum in the α/γ conformational space. These occur toward the end of the simulation trajectory and involve two known and allowed local minima (47). A few other bases show similar tendencies of much shorter duration, but overall the structure and individual base pairs remain well within the regions expected of the B-DNA conformational space. Identical analysis of the relevant parameters for the T_8_^Im^ duplex (Supplementary Figures S15, S17 and S19) demonstrates that the simulation trajectory of the modified systems features an overall conformational sampling that is highly similar to that of the wt trajectory. Changes are only apparent for the modified base itself (T_8_^Im^), the opposite base (T_21_) and the base pairs in the immediate vicinity (G_9_, C_10_, T_6_ and A_23_). For instance, when analyzing the B_I_/B_II_ torsional parameter for the T^Im^-modified base, the majority of structures still feature the DNA backbone either in the B_I_ or B_II_ conformation, however, some excursions outside these areas can be observed as well. Once a base pair is more than two positions up- or down-sequence from the modification site, no unexpected features can be discerned (see also Supplementary Figure S24 for the corresponding ^31^P spectra). Thus, the MD simulation trajectories are proposed to provide a good basis to explore the conformation and interaction of the ImH^+^ moiety in the T_8_^Im^ duplex and by extension in all modified duplexes.

### MD reveals a specific interaction in the T_8_^Im^ duplex

**Figure 4.**
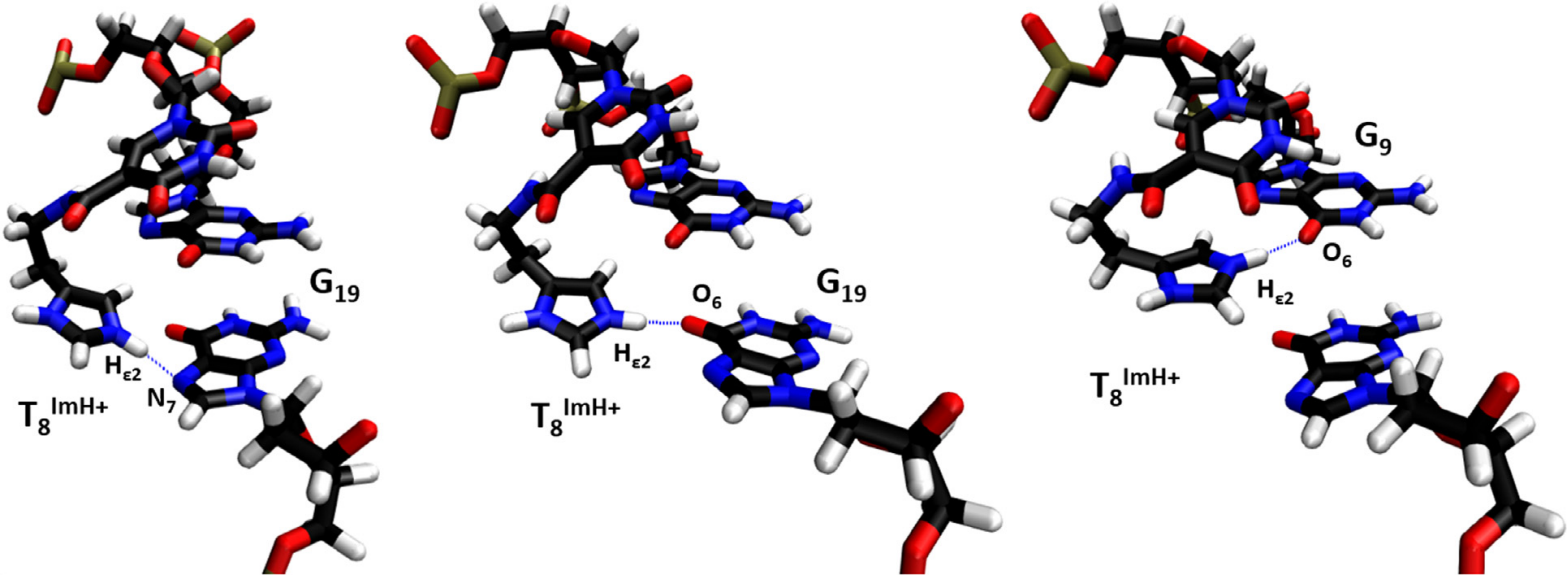
Three specific hydrogen bond patterns of the T_8_^ImH+^ functionality with the G_9_ and G_19_ bases from the Hoogsteen side. The respective hydrogen bond donor and acceptor are indicated in each snapshot. Visualized using VMD 1.9.1.

**Figure 5.**
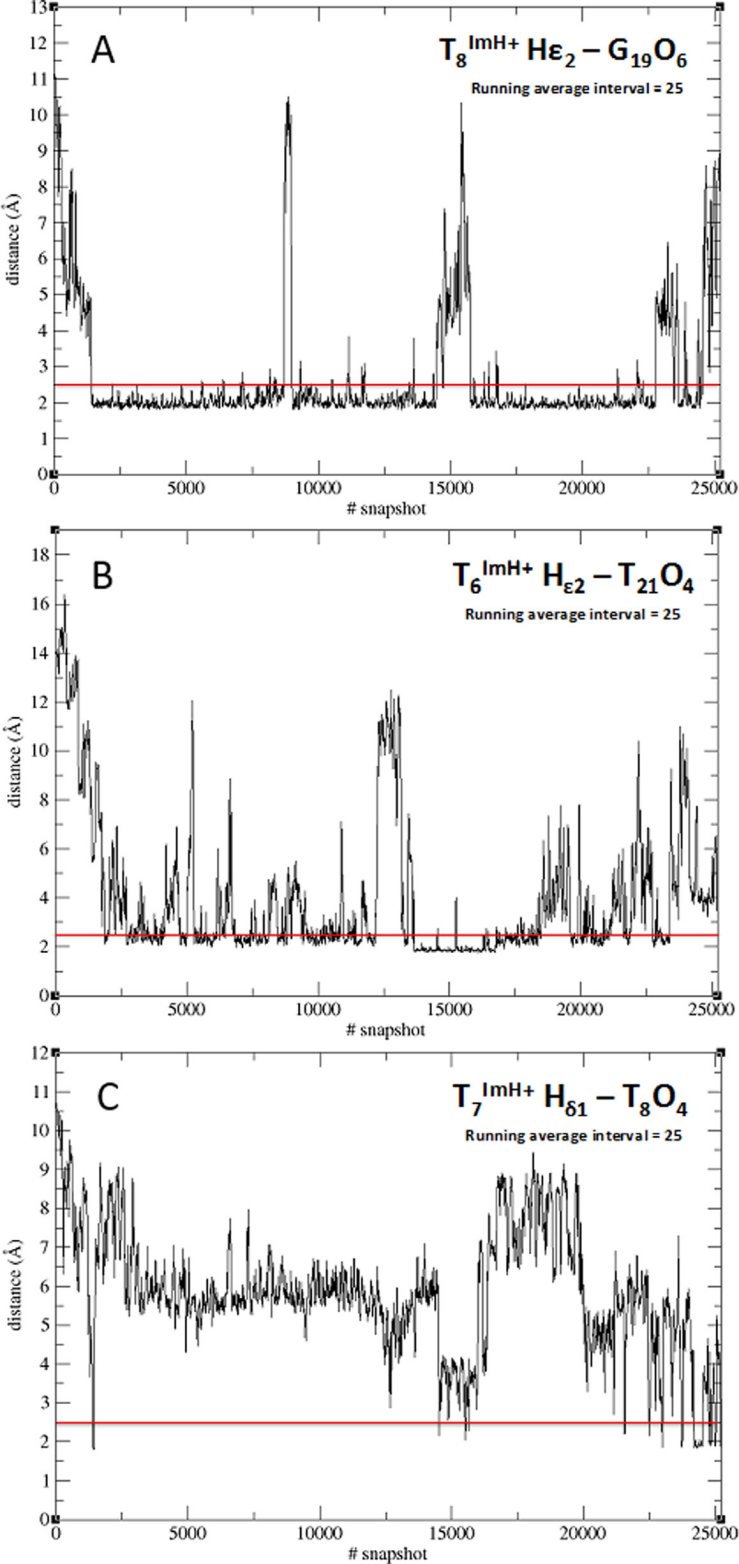
Overview of the persistence of the observed specific hydrogen bonds involving the T_6_^ImH+^, T_7_^ImH+^ and T_8_^ImH+^ bases in the corresponding duplexes. A running average over 25 structures is presented over the duration of the entire 50 ns trajectories.

Analysis of the hydrogen bond network involving the modified nucleoside over the MD simulation trajectory of T_8_^Im^ shows that a first hydrogen bond is initially formed after 2 ns between NHϵ2 of the imidazolium and N7 of G_19_, rapidly followed by hydrogen bond formation with the O6 along the Hoogsteen edge of G_19_ (Figure 4). Both the donor acceptor distance (<3 Å) and donor-hydrogen-acceptor angle (>150°) in the latter are satisfied for 30% of the simulation time. Even when both hydrogen bond criteria are not met, the donor-acceptor distance indicates that the imidazolium remains within the major groove, in close vicinity to the Hoogsteen edge of the G_9_C_20_ and C_10_G_19_ base pairs for over 90% of the total simulation time (Figure 5). This would also account for the 0.46 ppm upfield chemical shift change of the T_18_ methyl group, which is situated close to the face of the imidazolium group inside the major groove. Alternative but very low persistence hydrogen bond interactions involving N7 of G_19_ (1.3%) and O6 (1.8%) of G_9_ complete the hydrogen bond interaction pattern involving T^Im^ (Figures 4 and 5). In contrast to the T_8_^Im^ duplex, the imidazolium group in the other T_x_^Im^ duplexes remains overall solvent accessible only showing transient or low persistence interactions with the major groove of the duplex. These include low persistence hydrogen bonds formed between the Hδ1 of T^Im^ and the phosphate backbone oxygen of the preceding base: A_5_ (1.6%) in the T_6_^Im^ and T_6_ (8%) in the T_7_^Im^ duplexes. In addition, the imidazolium Hϵ2 proton in T_6_^Im^ appears involved in a hydrogen bond with the O4 carbonyl oxygen of the mismatched T_21_ base on the opposite strand for 11.2% of the total simulation time, while for T_7_^Im^ the O4 of the T_8_ base is involved, albeit only 2% of the simulation time (Figure 5).

When no productive hydrogen bond is formed, the imidazolium moiety is seen to make frequent (T_6_^Im^) or prolonged (T_7_^Im^) excursions away from the interaction sites toward the solvent, underlining the transient nature of the interactions. This compares well with the chemical shift perturbation data (see Supplementary Figures S5 and S6), where perturbations are limited to the base pair and the immediately neighboring base pairs. As for the nOe data, only few nOe-contacts are present in these T_x_^Im^ duplexes, which supports the transient nature of the hydrogen-bonding interactions inferred from the MD simulation. Thus, the behavior of both the T_6_^Im^ and T_7_^Im^ duplexes appears quite similar. In the MD simulation trajectory of T_21_^Im^, transient hydrogen bonds involving NHδ1 from the imidazole and a phosphate oxygen of either T_21_ or C_20_ as acceptor can be found for 7.5% and 12.8% of the simulation time. No other acceptors for hydrogen bonds involving the surrounding bases could be identified, in contrast with T_6_^Im^ and T_7_^Im^. Again, frequent excursions of the imidazole moiety toward the linker are present (see Supplementary Figure S20). Since the chemical shift perturbation and nOe data is likewise limited to the effects mentioned for the T_6_^Im^ and T_7_^Im^ duplexes, good agreement is maintained between the experimental data and the simulation.

### Confrontation of nOe and simulation data for the T_8_^Im^ duplex

**Figure 6.**
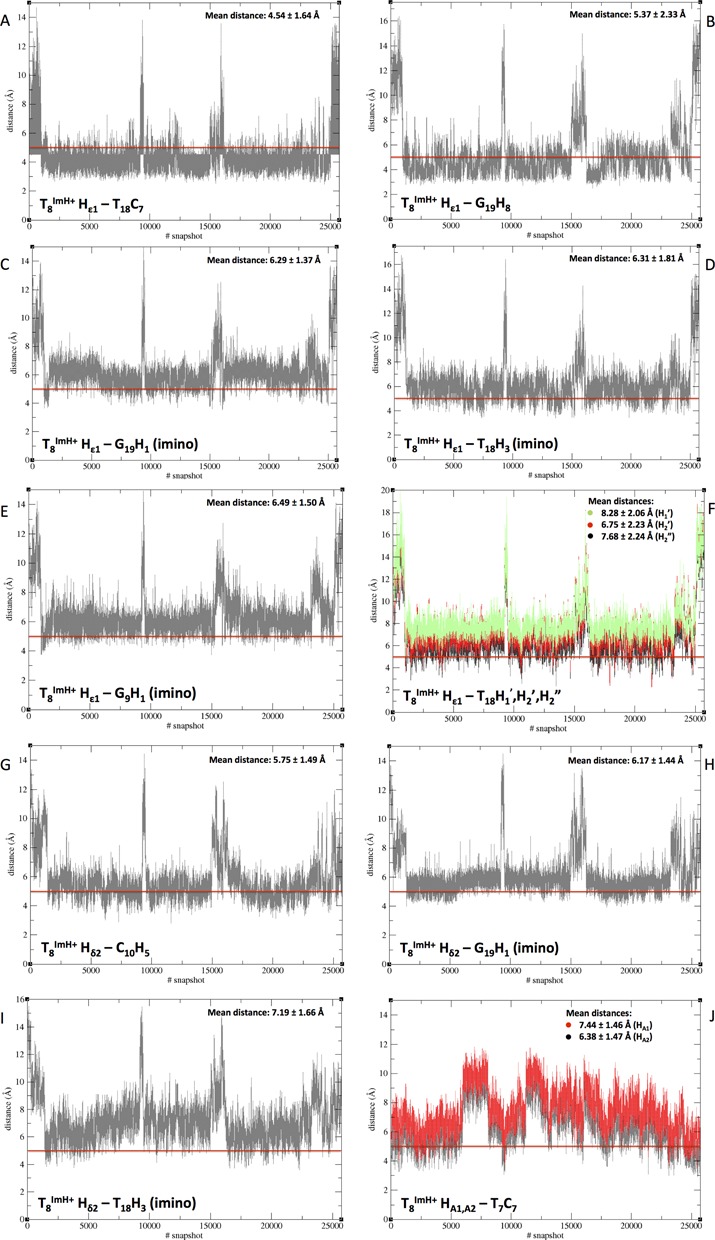
Overview of the extracted distances from the 50 ns trajectory run of T_8_^Im^ which are confronted with the nOe contact limit at 5Å, indicated by the red line on each graph. T^ImH+^ explicitly refers to the protonated imidazolium state of the T^Im^ nucleoside at position 8. The abscissa refers to snapshots and can be converted to time (ps) by multiplying with two (one shot per two seconds).

A total of 13 nOe's unambiguously assigned from the NOESY spectrum of T_8_^Im^ as shown in Figure 3B were matched to the simulation by extracting the associated interproton distance as a function of simulation time (Figure 6). These distances should consistently dip below the 5 Å mark in order to generate the observed nOe's. Since the simulation starts with an extended conformation for the T^ImH+^ modification, each distance starts around 10–12 Å, and rapidly decreases as the imidazole moiety folds back on the duplex. Over the entire simulation trajectory, matching of the interproton distances corresponding to the eight nOe contacts involving the resolved Hϵ1 resonance establishes that the one to the T_18_ methyl averages below 5 Å (Figure 6A). The other distances associated with clearly discernable nOe contacts make regular excursions below 5 Å but average above the threshold value. The distance to G_19_ H8 satisfies the threshold for half the simulation time in good agreement with the nOe data (Figure 6B). The Hϵ1–G_19_ H1 (imino) distance, generally close and on average above the threshold is indeed one of the weaker nOe contacts (Figure 6C). The same holds true for the T_8_^Im^ Hϵ1–T_18_ H3 (imino) (Figure 6D) and T_8_^Im^ Hϵ1–G_9_ H1 (imino) distance (Figure 6E). Only the T_8_^Im^ Hϵ1–T_18_ H1′ distance (Figure 6F, green), also showing the weakest nOe contact, does not drop below 5 Å. Overall, the average distance values are in good agreement with the relative intensities of the nOe contacts. In view of the weakness of some nOe contacts, especially those involving the T_18_ deoxyribose protons (Figure 6F), the more important deviations from the threshold value could actually result from spin-diffusion via common neighboring spins thereby underestimating the actual distance.

Hδ2, the other non-exchangeable proton present on the imidazole functionality, also shows a number of characteristic nOe contacts. Because this resonance is located in the H8-H6 region of the ^1^H spectrum, assignment of relevant nOe contacts for analysis is difficult. Nevertheless, three characteristic through space contacts can be accessed: T_8_^Im^ Hδ2–C_10_ H5, T_8_^Im^ Hδ2–G_19_ H1 and T_8_^Im^ Hδ2–T_18_ H3 (imino). As is the case with Hϵ1, the contacts of Hδ2 concerning the imino protons (G_19_ H1 and T_18_ H3 (imino)) are weak, hereby suggesting the extracted distances with the respective protons should be close to 5 Å (Figure 6H and I). These average to 6.17 and 7.19Å, respectively. On the other hand, the clear nOe to C_10_ H5 is associated with an average distance of 5.75 Å (Figure 6G), and regularly dips below 5 Å in the simulation.

The final relevant nOe contacts concern the HA1 and HA2 protons of the ethylene tether closest to the amide bond, connecting the imidazole ring to the T_8_^Im^ base. As could be expected, these protons are in spatial proximity to neighboring T_7_ methyl (Figure 6J, HA1 red and HA2 black), but yield distances averaging between 6.38 and 7.44 Å.

Thus, of the set of 13 nOe's, the most intense ones are seen to correspond to distances close to or below the 5 Å threshold, while weaker nOe's generate distances above, which correlate well with the ranking of the nOe intensities. Taking into account the unrestrained nature of the MD simulation, the overall agreement with the nOe data appears very satisfactory.

For all the T_x_^ImH+^ systems studied so far, the unrestrained MD confirms that only the T_8_^ImH+^ system shows a sustainable and specific hydrogen bond pattern of its imidazole functionality with the DNA scaffold. T_6_^ImH+^, T_7_^ImH+^ and even T_21_^ImH+^ on the other hand do not. These observations substantiate the proposal that the markedly higher pKa_H_ for T_8_^ImH+^ is linked to the existence of a specific interaction motif. This motif apparently arises in a three base pair cassette composed of the modified T^Im^ base pair followed by a GC/CG sequence allowing for interaction between the imidazole and the hoogsteen side of a guanine at position +2 (opposing strand, here G_19_) or the guanine at position +1 (same strand, here G_9_) from the modification.

### Importance of the T_8_-T_21_ mismatch: should it stay or can it go?

An obvious question is whether a T-T mismatch is a prerequisite for the occurrence of this motif. Considering T_8_^Im^A_21_ in Table 1, the obtained pKa_H_ of 8.72 ± 0.02 is very close to the original value of 8.88 ± 0.05 for T_8_^ImH+^. Similarly, with respect to the unmodified T_8_A_21_ wt sequence, the melting temperature at pH 7.0 has increased with 5.2°C thereby indicating a significant rise in stability. The chemical shift of the T_18_ methyl resonance, a fingerprint for the interacting imidazolium group, also moves 0.44 ppm up-field as observed before in the T_8_^Im^ duplex. Analyzing the nOe data involving the imidazolium modification for T_8_^Im^A_21_, a set of near-identical nOe contacts can be found (Figure 3A), indicating the integrity and similarity of the interaction in the absence of the T-T mismatch. When mapping the specific nOe contacts involving T^ImH+^ to the duplex, all the contacts present in the T_8_^Im^ system also can be seen with T_8_^ImH^A_21_. Three additional nOe contacts are visible as well (Figure 3A). The nOe connecting T_8_^Im^ Hϵ1 to C_20_ H5 averages to 6.67 ± 2.30 Å, and makes regular excursions below the 5 Å threshold. The other two nOe's also involving Hϵ1 are either satisfied to a lesser extent or deemed to far as indicated from their average distances found to be 8.84 ± 2.47 Å for T_18_ H3′ and 9.70 ± 1.72 Å for C_17_ H3′, respectively. It is probable that spin diffusion, possibly enhanced due to rigidifaction as a result of removing the T-T mismatch, is the underlying reason for the appearance of these last two nOe contacts. Nevertheless, the new C_20_ H5 nOe contact appears to indicate the possibility of a somewhat different local conformation that still includes the interaction to the G_9_-C_20_ base pair.

The comparison of the hydrogen bond pattern obtained from the complete MD trajectories for the T_8_^ImH+^ and T_8_^ImH+^A_21_ duplexes does not reveal a significant difference (see Supplementary Figure S21). During the trajectory, the T^ImH+^ functionality in the T_8_^Im^ duplex engages in three distinct hydrogen bond patterns of which the T_8_^Im^ NHϵ2–O6 G_19_ is most persistent (29.7%), with transiently populated alternatives (<2%) involving the O6 of G_9_ or N7 of G_19_ (*vide supra*). In case of the T_8_^Im^A_21_ duplex, only two hydrogen bonds are present: the T_8_^ImH+^ NHϵ2–O6 G_19_ one, which is almost equally persistant (27.1%), and T_8_^ImH+^ NHϵ2 –O6 G_9_ that is again only transiently populated (1.5%). Thus, no notable differences are observed with regards to the MD trajectories of both duplexes. In conclusion, all experimental and simulation data indicate the preservation of the specific imidazolium–DNA hydrogen bond interaction in T_8_^ImH+^A_21_, proving that the T_8_-T_21_ mismatch, though present in the current system for a well-defined reason (*vide supra*), is not a necessity for an imidazolium-mediated duplex interaction and concomitant stabilization. This further confirms the exact composition of the motif to consist of a 3 base pair ‘cassette’ wherein the T^Im^ nucleotide is followed by a GC/CG sequence and where the exact nature of the T^Im^ pairing partner base can be either T or A.

### General applicability of the new motif: built to last?

**Figure 7.**
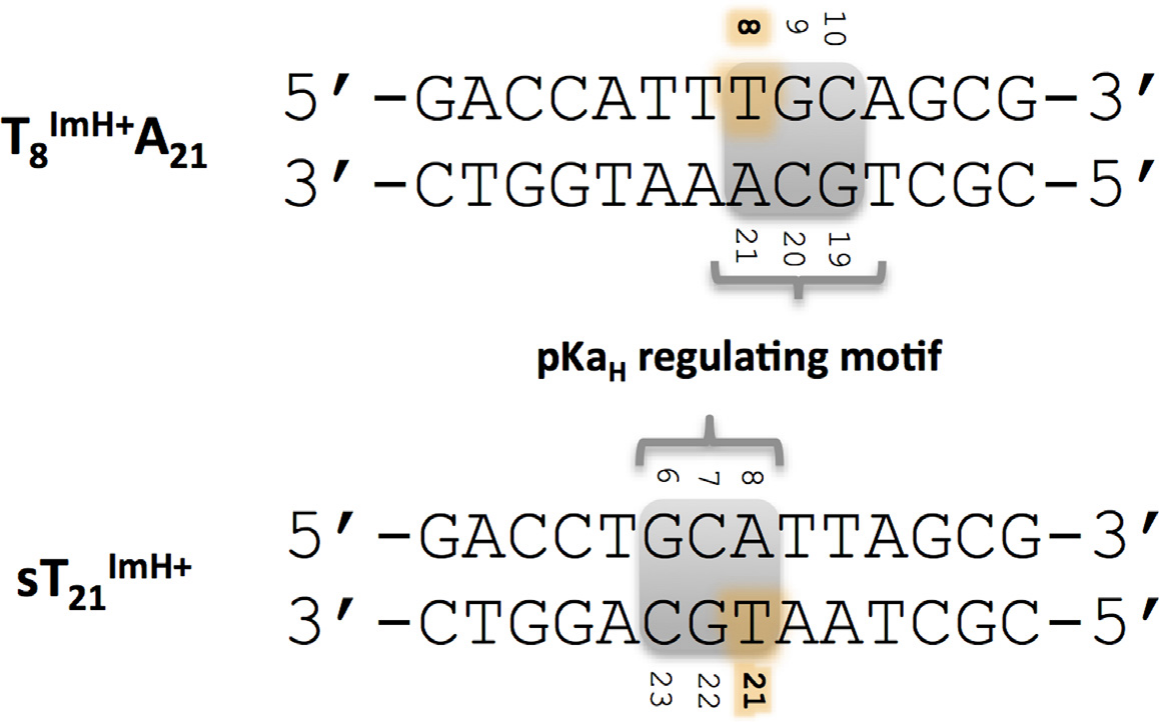
Comparison of the original motif in the T_8_^Im^A_21_ system and the same motif in a new scrambled sequence sT_21_^Im^.

How robust is this potentially pKa_H_-regulating motif? In other words, if this cassette would be introduced in a different sequence, will the specific interaction still be intact? This was addressed via a ‘scrambled’ modified and non-mismatched duplex, sT_21_^Im^ (Figure 7).

Compared to the original T_8_^Im^A_21_ duplex, the cassette was translated three positions and rotated 180° to yield a G_6_C_7_A_8_•T_21_G_22_C_23_ sequence, expected to generate the pKa_H_-regulating motif. The A_5_T_24_ base pair, preceding the motif in the wt-duplex, was flipped to T_5_A_24_, as this would position the T_24_ methyl in a similar position for an up-field chemical shift as observed in the T_8_^Im^ and T_8_^Im^A_21_ duplexes. The melting temperature of sT_21_^Im^ shows an increase of 6.2°C compared to the unmodified sT_21_ duplex, and the pKa_H_ is 9.07 (Table 1). Both values are clearly in line with those for the T_8_^Im^A/T duplexes indicating the presence of the interaction with the imidazolium group. In addition, a similar chemical shift perturbation profile (see supplementary Figure S10), including a 0.44-ppm up-field shift of the T_5_ methyl, and a set of specific nOe contacts can be observed (Figure 3A), concomitant with the expected T_21_^Im^ NHϵ2–O6 G_6_ hydrogen bond in the MD trajectory, with a 35.3% persistence. These data provide final confirmation of the viability of the cassette as a pKa_H_-regulating motif in DNA duplex sequences.

## CONCLUSIONS AND OUTLOOK

Imidazole residues have been engineered into the major groove of a DNA duplex. As demonstrated by detailed molecular modeling studies and experimentally verified by NMR data, a new functional imidazolium-based motif has been uncovered that increases the imidazolium pKa_H_, with respect to the free modified nucleoside, by more than one unit. Having established the general applicability of this pKa_H_-regulating motif, a summary of the different properties can be extracted. These in turn can be used as a checklist to confirm whether the specific interaction is present or absent. The modified system suspected of having a specific hydrogen bond interaction between the imidazole functionality and its DNA backbone must feature (i) a general increase in melting temperature of 6 ± 1°C with respect to the non-modified wt sequence, (ii) show a clear downfield shift in the 1D ^1^H spectrum of the Hϵ1 resonance to ≈9.0 ppm in the presence of the interaction as opposed to ≈8.7 ppm without the hydrogen bridge, (iii) demonstrate a clear and specific set of 2D nOe contacts starting from Hϵ1 of the modification at position *n* to the base at position *n* + 3 of the opposite chain, (iv) have a significant increase in pKa_H_ of the T^ImH+^-modification to ≈8.7–9.0 or higher, where in the absence of the motif the pKa_H_ is located around a lower value of ≈7.9. Fulfilling these requirements guarantees that the system at hand does exhibit the specific imidazole pKa_H_-increasing interaction. The exploration of duplexes carrying multiple T^Im^ modifications wherein one or more pKa_H_ motifs contribute will be the object of future investigations using our combined NMR and MD approach.

## SUPPLEMENTARY DATA

Supplementary Data are available at NAR Online.

SUPPLEMENTARY DATA
