# Supplementary material for: Identification of a pKa-regulating motif stabilizing imidazole-modified double-stranded DNA
Source: Nucleic Acids Res. 2014 Dec 17;43(1):51–62. doi: 10.1093/nar/gku1306 (PMC4288195; doi:10.1093/nar/gku1306)
Supplement: SUPPLEMENTARY DATA [file supp_43_1_51__index.html]

Identification of a pKa-regulating motif stabilizing imidazole-modified double-stranded DNA — Identification of a pKa-regulating motif stabilizing imidazole-modified double-stranded DNA — SUPPLEMENTARY DATA 

# Identification of a pKa-regulating motif stabilizing imidazole-modified double-stranded DNA

## SUPPLEMENTARY DATA

**Files in this Data Supplement:**

- SUPPLEMENTARY DATA
