# Supplementary material for: Identification of a pKa-regulating motif stabilizing imidazole-modified double-stranded DNA
Source: Nucleic Acids Res. 2014 Dec 17;43(1):51–62. doi: 10.1093/nar/gku1306 (PMC4288195; doi:10.1093/nar/gku1306)
Supplement: SUPPLEMENTARY DATA [file supp_gku1306_nar-01928-f-2014-File002.pdf]

SUPPORTING INFORMATION – IDENTIFICATION OF A PKA-REGULATING MOTIF STABILIZING IMIDAZOLE MODIFIED DOUBLE STRANDED DNA

Dieter Buyst[1], Vicky Gheerardijn[2], Krisztina Fehér[1], Bjorn Van Gasse[1], Jos Van Den Begin[2], José C. Martins[1,]* and Annemieke Madder[2,]*

[1] Department of Organic and Macromolecular Chemistry, NMR and Structure Analysis Unit, Ghent University, Gent, Oost-Vlaanderen, 9000, Belgium
[2] Department of Organic and Macromolecular Chemistry, Organic and Biomometic Chemistry Research Group, Ghent University, Gent, Oost-Vlaanderen, 9000, Belgium

*Corresponding Authors: Annemieke.Madder@UGent.be & Jose.Martins@UGent.be

Table of Contents

Synthesis of the T^{Im} modified building block S1
Synthesis of T_x^{Im} modified oligonucleotides S6
Concentration determination S6
General assignment of all (non-) exchangeable ^{1}H resonances S6
Chemical shift perturbation overview of all T_x^{Im} modified duplexes S7
- T_6^{Im} modified duplex S8
- T_7^{Im} modified duplex S9
- T_{21}^{Im} modified duplex S10
- T_8^{Im} modified duplex S11
- $T_8^{Im}A_{21}$ modified duplex S12
- sT_{21}^{Im} modified duplex S13
- $A_8T_{21}^{Im}$ modified duplex S14

pKaH regulating motif in $T_8^{Im}A_{21}$ and sT_{21}^{Im} S15
Validation molecular dynamics trajectory: wild type and T_8^{Im} S16
- Conformational α/γ space sampling wild type S16
- Conformational α/γ space sampling T_8^{Im} S20
- B_I/B_{II} space sampling wild type S24
- B_I/B_{II} space sampling T_8^{Im} S29
- Zp/Zp(h) space sampling wild type S33
- Zp/Zp(h) space sampling T_8^{Im} S36

Hydrogen bond persistence T_{21}^{Im} duplex S39
Comparison hydrogen bond persistence $T_8^{Im}A_{21}$ versus T_8^{Im} S40
Overview specific Hε1 nOe-contacts S41
Overview specific Hε1 nOe-contacts to exchangeable protons S42
31P spectrum of the T_8^{Im} and wild type systems S43
Extended melting temperatures table including thermodynamic data S44

Synthesis of the T^{Im} modified building block

All chemicals and solvents (Sigma-Aldrich, Fluka, Acros) were purchased and used without any further purification, except dichloromethane, which was distilled from CaH_2 prior to use. All reactions were performed under argon or nitrogen with magnetic stirring and were monitored by thin layer chromatography (TLC) using SIL G-25 UV254 pre-coated silica gel plates (0.25 mm thickness). Reactions under CO atmosphere were carried out in a Parr-High-Pressure reaction vessel with particular care. TLC plates were visualized by using anisaldehyde (5% anisaldehyde in ethanol with 1% sulfuric acid) or PMA (5 % phosphomolybdic acid in ethanol) solutions. Flash column chromatography was performed by using BIOSOLVE silica gel (0.063–0.200 mm particle size, 20–30 g silica/ 1 g compound/ KieselgelMerck, 230-400 mesh, Type 9385, 60 Angström). ^{1}H NMR spectra were recorded at 300 MHz, ^{13}C NMR spectra were recorded at 75 MHz. Chemical shifts (δ) are reported in units of parts per million (ppm), with the residual ^{1}H or ^{13}C peaks of the solvent used as internal standards ($(CD_3)_2SO$: δH = 2.50 ppm and δC = 39.52 ppm; $CDCl_3$: δH = 7.26 ppm and δC = 77.16 ppm). The following abbreviations are used to explain the observed multiplicities: s, singlet; d, doublet; t, triplet; q, quadruplet; m, multiplet; br, broad; band, several overlapping signals; AB, AB system with strongly skewed signals; app, indicates an "apparent" multiplicity, for which only the observed average coupling constant can be quoted, in absence of information on the real J-values. Where given, assignments of resonances were confirmed by standard COSY and HSQC 2D NMR experiments. High resolution mass spectra (HRMS) were recorded with an Agilent Accurate-Mass Quadrupole Time-of- Flight mass spectrometer.

*5-(2-(1-(tert-Butoxycarbonyl)imidazole-4-yl)ethylaminocarbonyl)-2'-deoxyuridine, **1**.* 5-Iodo-2'-deoxyuridine (1 g, 2.8 mmol) was dissolved in anhydrous DMF (20 ml) and histamine (3 eq., 944 mg, 8.5 mmol), Et_3N (6 eq., 2.4 ml, 17 mmol) and $Pd(PPh_3)_4$ (0.05 eq., 162 mg, 0.14 mmol) were added in the order listed. The reaction mixture was incubated in a Parr-High-Pressure reaction vessel at 70 °C under 50 psi (4 bar) carbon monoxide for 48 h. The reaction mixture was cooled to room temperature before being filtered over celite and concentrated under vacuum to an oil. The residu was taken up in anhydrous DMF (10 ml) and to the stirring solution under argon, Et_3N (3 eq., 1.2 ml, 8.5 mmol) and *tert*-butyl dicarbonate (1.5 eq., 930 mg, 4.2 mmol) was added. After 15-30 min stirring at room temperature, the reaction was quenched with anhydrous methanol (5 mL), stirred for 10 min. and again concentrated to an oil under vacuum. The oil was then purified on a silica gel column using a gradient of 5-10 % methanol in dichloromethane as eluent to give a white foam (860 mg, 65 %). R_f = 0.23 (CH_2Cl_2: CH_3OH 9:1). ^{1}H NMR ($(CD_3)_2SO$, 300 MHz): δ 11.84 (s (br), 1H), 8.78 (t, *J*= 6.5 Hz, ,1H), 8.68 (s, 1H), 8.11 (d app, *J*= 1.2 Hz, 1H), 7.31 (d app, *J*= 1.2 Hz, 1H), 6.11 (t app, *J*= 6.6 Hz, 1H), 5.28 (d, *J*= 4.1 Hz, 1H), 5.00 (t, *J*= 4.14 Hz, 1H), 4.22 (m, 1H), 3.86 (q, *J*= 3.7 Hz, 1H), 3.56 (t, *J*= 3.7 Hz, 2H), 3.52 (q, *J*= 6.5 Hz, 2H), 2.69 (t, *J*= 6.5 Hz, 2H), 2.21 (ddd AB, *J*= 3.6, 6.6 and 13.7 Hz, 1H), 2.12 (dt AB, *J*= 6.6 and 13.7 Hz, 1H), 1.56 (s, 9H). ^{13}C APT NMR ($(CD_3)_2SO$, 75 MHz): δ 163.1, 161.4, 149.5, 145.8, 140.8, 123.9, 113.6, 105.3, 87.9, 85.5, 85.1, 70.4, 61.2, 45.7, 37.9, 27.8, 27.4; HRMS (ESI): *m/z*: calcd for $C_{20}H_{26}O_8N_5$: 464.1781 $[M-H]^-$; found: 464.1791.

*5-(2-(1-(tert-Butoxycarbonyl)imidazole-4-yl)ethylaminocarbonyl)-5'-O-(4,4'-dimethoxytrityl)-2'-deoxyuridine, **2***. Compound 1 (386 mg, 0.83 mmol) was dried by co-evaporation with dry pyridine (3 x 5 mL), dried overnight under vacuum and dissolved in dry pyridine (5 mL) and dry DCM (2 mL). 4,4'-dimethoxytrityl chloride (1.2 eq., 338 mg, 1 mmol), dissolved in a mixture of dry pyridine (3 mL) and dry DCM (1 mL), was slowly added to the stirring nucleoside solution at 0°C. The reaction was stirred overnight at room temperature and quenched by the addition of anhydrous methanol (3 mL). The solvent was removed under reduced pressure and the residue re-dissolved in DCM (30 mL) and washed with $NaHCO_3$ aq, sat. (30 mL) and brine (30 mL). The combined organic phases were dried on Na_2SO_4 and reconcentrated under vacuum to an oil. The oil was then purified by chromatography on silica gel using a gradient of 0-5 % methanol in dichloromethane + 1 % Et_3N to give a white foam (420 mg, 66 %). Rf = 0.53 (CH_2Cl_2: CH_3OH 9:1). ^{1}H NMR ($CDCl_3$, 300 MHz): δ 9.17 (t, *J*= 6.9 Hz, 1H), 8.54 (s, 1H), 8.11 (d app, *J*= 1.3 Hz, 1H), 7.17-7.42 (band, 10H), 6.83 (m, 4H), 6.20 (t app, *J*= 6.5 Hz, 1H), 4.35 (dt, *J*= 5.0 and 6.5 Hz, 1H), 3.95 (q app, *J*= 5.0 Hz, 1H), 3.78 (s, 6H), 3.66 (q, *J*= 6.9 Hz, 2H), 3.51 (dd AB, *J*= 5.0 and 10.2 Hz, 1H), 3.36 (dd AB, *J*= 5.0 and 10.2 Hz, 1H), 2.87 (t, *J*= 6.9 Hz, 2H), 2.46 (ddd AB, *J*= 5.0, 6.5 and 13.7 Hz, 1H), 2.23 (dt AB, *J*= 6.5 and 13.7 Hz, 1H), 1.60 (s, 9H). ^{13}C APT NMR ($CDCl_3$, 75 MHz): δ 163.3, 161.8, 158.7, 150.0,, 147.0, 145.7, 144.7, 140.8, 135.8, 130.2, 128.2, 128.1, 127.1, 114.0, 113.4, 106.8, 87.0, 85.8, 85.7, 85.4, 72.6, 63.9, 55.4, 46.1, 40.3, 39.5, 28.4, 28.1; HRMS (ESI): *m/z*: calcd for $C_{41}H_{44}O_{10}N_5$: 766.3088 $[M-H]^-$; found: 766.3081.

*5-(2-(1-(tert-Butoxycarbonyl)imidazole-4-yl)ethylaminocarbonyl)-5'-O-(4,4'-dimethoxytrityl)-3'-(2-cyanoethyl-N,N'-diisopropylphosphoramidite)-2'-deoxyuridine, **3***. The DMTr-protected nucleoside 2 (395 mg, 0.51 mmol) was evaporation 3x with dry DCM and further dried in vacuo for 2-3h. The resulting foam was dissolved in dry DCM (15 mL) and cooled in an ice bath. To the stirring nucleoside solution, DIPEA (4 eq., 0.36 mL, 2 mmol) and 2-cyanoethyl-N,N'-diisopropylchlorophosphoramidite (2 eq., 0.23 mL, 1 mmol) was added dropwise. The reaction mixture was stirred for 1 h at room temperature and quenched with 5 % $NaHCO_3$ aq.

sol. (2 mL) at 0 °C. The mixture was then diluted with DCM (30 mL) and washed with 5 % $NaHCO_3$ aq. sol. (40 mL) and brine (3x 40 mL). The combined organic phases were dried on Na_2SO_4 and re-concentrated under vacuum to yield a white foam. The product was used without further purification (428 mg, quantitative conversion). Rf = 0.62, 0.66 (CH_2Cl_2: CH_3OH 9:1). ^{31}P NMR ($CDCl_3$, 121 MHz): δ 149.14, 148.74 ppm. HRMS (ESI): m/z: calcd for $C_{50}H_{61}O_{11}N_7P$: 966.4166 $[M-H]^-$; found: 966.4153.

Reagents for DNA synthesis were obtained from Proligo Reagents (Sigma-Aldrich). Non-modified complementary strands were purchased from Integrated DNA Technologies. All RP-HPLC data of the modified oligonucleotides were recorded on an Agilent 1200 system equipped with a Phenomenex Clarity Column (250 x 4.6 mm, 5 μm) at 50 °C with 0.1 M TEAA buffer (with 8 % ACN) and acetonitrile as mobile phase (linear gradient: 0-30 % ACN in 15 min, 30-100 % ACN in 3 min.). The chromatographs were analyzed at 260nm. Mass spectra analysis of the oligonucleotides was performed on a Agilent G1946C LC/MSD-VL 1100 Series HPLC equipped with API-ESI source. Data were acquired in the negative ionization mode from m/z = 50 to m/z = 1500. The mass spectra were deconvoluted; i.e., the molecular weights of the oligonucleotides were reconstructed from the spectra using the Agilent LC/MSD Chemstation software (version A.08014).

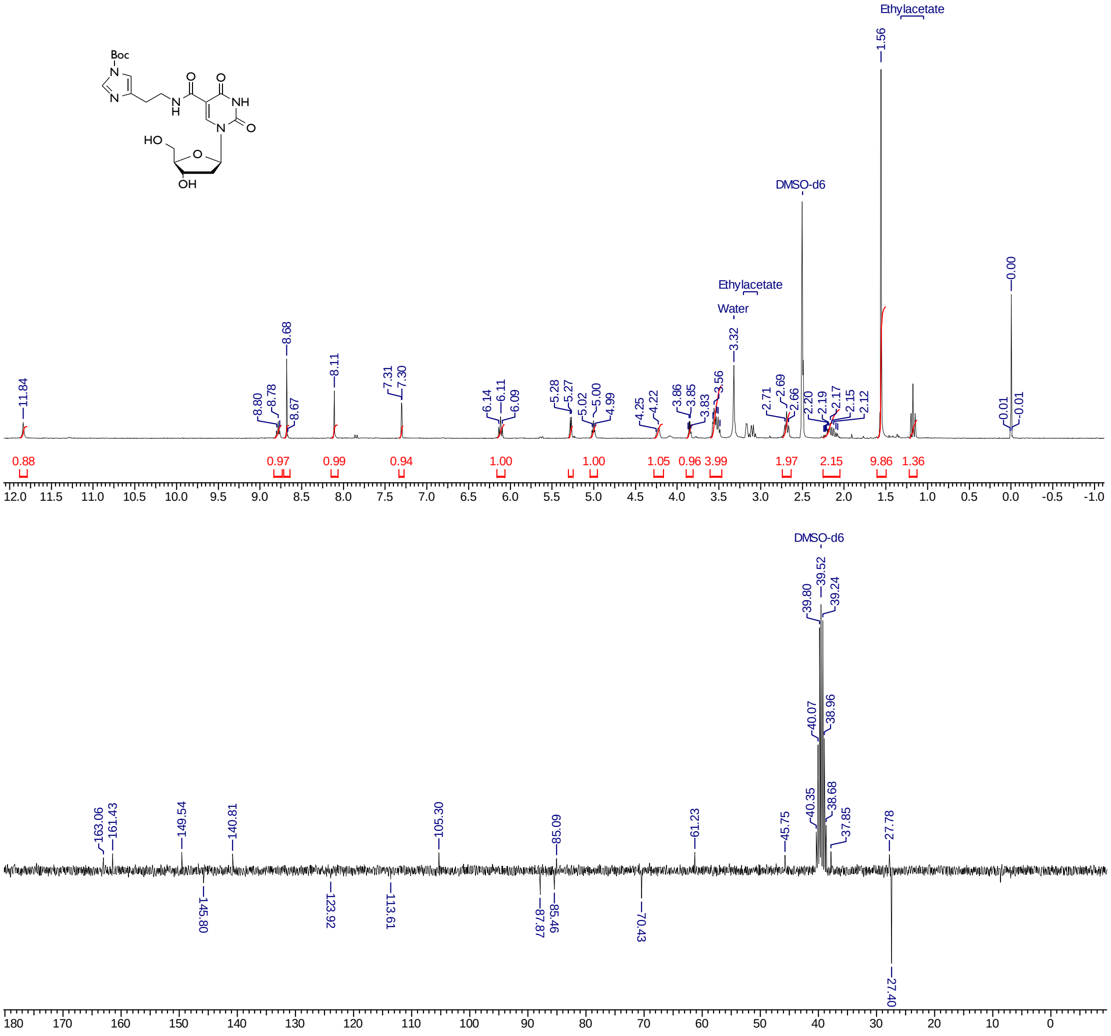

Figure S1. ^{1}H and ^{13}C spectrum of the T^{Im} modified building block synthesis after the CO-insertion Pd-cross coupling reaction (25°C, 300MHz, $(CD_3)_2SO$-d6, traces of EtOAc and water).

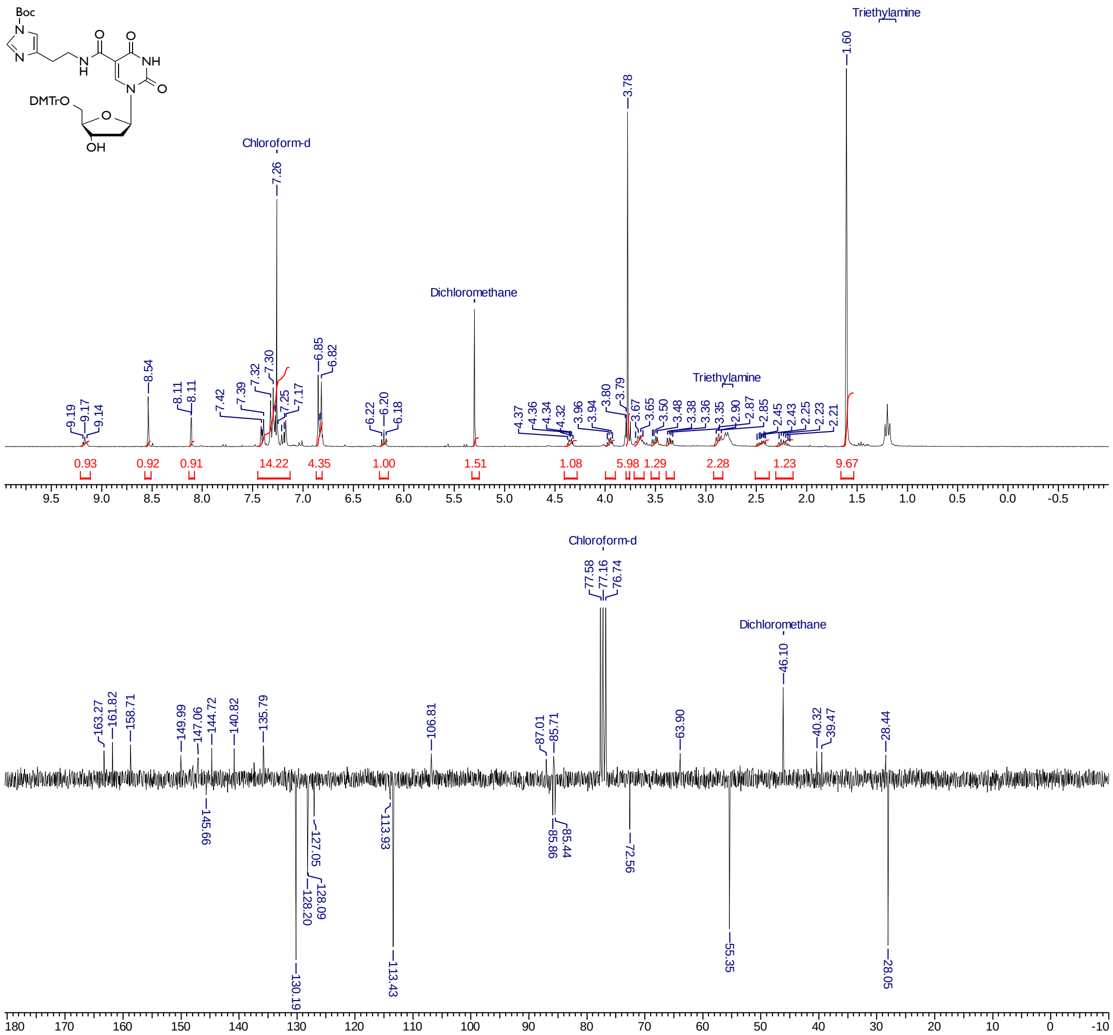

Figure S2. ^{1}H and ^{13}C of the T^{Im} modified building block synthesis after the DMTr-protection (25°C, 300MHz, $CDCl_3$, traces of Et_3N and CH_2Cl_2).

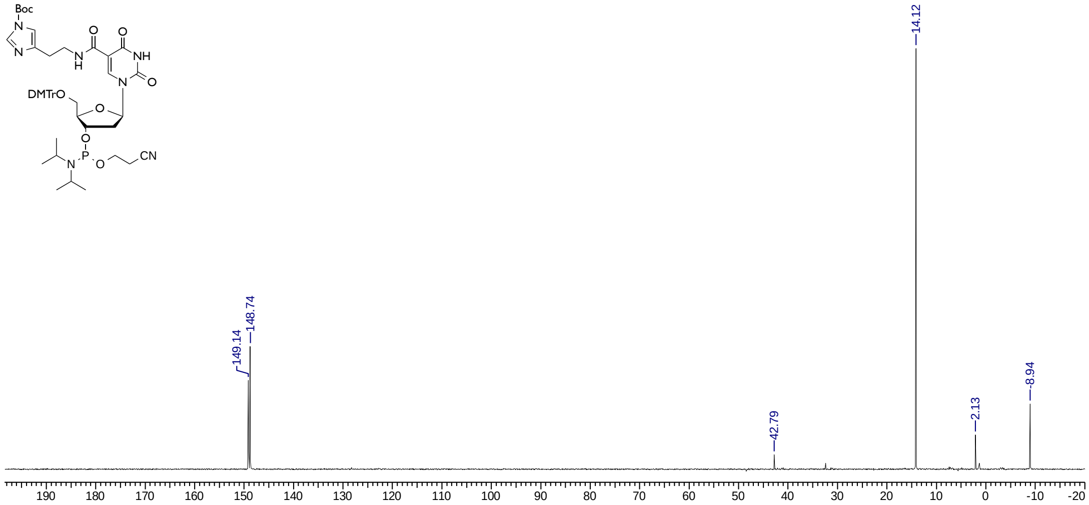

Figure S3. ^{31}P spectrum of the T^{Im} modified phosphoramidite (25°C, 300MHz, $CDCl_3$, traces of hydrolyzed products, phosphoramidite used without purification)

Synthesis of T_x^{Im} modified oligonucleotides.
All oligonucleotides were synthesized DMTr-ON using an ABI 394 DNA synthesizer at 1 μmol scale. Standard automated synthesis protocol was used except for coupling of the modified phosphoramidites. The synthesis column was removed from the automated DNA synthesizer for the manual coupling of the modified residues. For this introduction, 0.2 M solution of the modified phosphoramidite in dry acetonitrile (0.4 mL) and 0.1 M solution of DCI in dry acetonitrile (0.5 mL) were mixed together in a syringe and pushed in small portions (0.05 mL) over the reaction column (total coupling time 15 min.). The column was then re-installed on the synthesizer and automated synthesis was resumed.
The synthesized oligonucleotides were cleaved from solid support and deprotected by incubation at 55°C overnight in a concentrated aqueous ammonia solution. After cleavage and deprotection the samples were purified on SEP-PAK® C18 cartridges (obtained from Waters).

Concentration determination.
All experiments to determine the concentration of oligonucleotides were performed on a Trinean DropSense® 96 equipped with DropPlate® reader. Concentrations were calculated using the DropQuant® software.

General assignment of all (non-) exchangeable ^{1}H resonances
A first step in any NMR study is the assignment of the resonances of interest. Non-labile ^{1}H resonances were assigned using the standard sequential assignment procedures. The imino protons have to be assigned separately. For all duplexes, the imino protons can be divided into three groups based on their chemical shifts: (i) guanine imino protons involved in G•C base-pairs resonating between 13.2 and 14.7 ppm, (ii) thymine imino protons involved in A•T base-pairs resonating between 12.3 and 13.1 ppm and (iii) the imino protons of the T•T mismatched base-pair resonating between 10 and 11.2 ppm. Since imino protons of adjacent base-pairs are constrained to be approximately 3.4 Å below or above one another in a B- DNA duplex, imino proton resonances can be assigned based on nOe contacts between imino protons of adjacent base-pairs.

A general overview of the imino region of the wild type sequence and the studied alternative sequences can be seen in figure S4. The corresponding names of the different sequences indicated next to their respective spectra refer to the base pairs altered with respect to the numbering in the original wild type sequence.

For all duplexes, all imino proton resonances were visible, apart from the G1 and G14 imino protons, belonging to the terminal base pairs. For the wild type duplex, two additional, unassigned resonances are visible at the lowest temperature, which can most likely be attributed to the imino protons of the terminal base pair, although it was not possible to make a definitive assignment based on the 2D NOESY spectra. However, since a similar pattern of nOe contacts can be observed for both imino proton resonances of the T•T mismatch, it is not possible to distinguish these two resonances from one another using the traditional imino assignment strategy based on 2D NOESY spectra. Therefore, the assignment of the imino protons of the mismatched base pair had to be accomplished via the site-specific incorporation of a partially ^{15}N-labelled thymidine nucleotide, either at position T8 or T21. As was illustrated previously, such a site-specific, partial ^{15}N-incorporation can aid the assignment of the labeled spins, while limiting the cost compared to a site-specific 100% enrichment. For the T_8^{Im} and T_{21}^{Im} duplexes, the ^{15}N-labelled nucleotide was incorporated opposite the chemically modified nucleotide, at positions T21 and T8 respectively. For the T_6^{Im} and T_7^{Im} duplexes, position T_8 was arbitrarily chosen for the incorporation of the ^{15}N-labelled nucleotide.

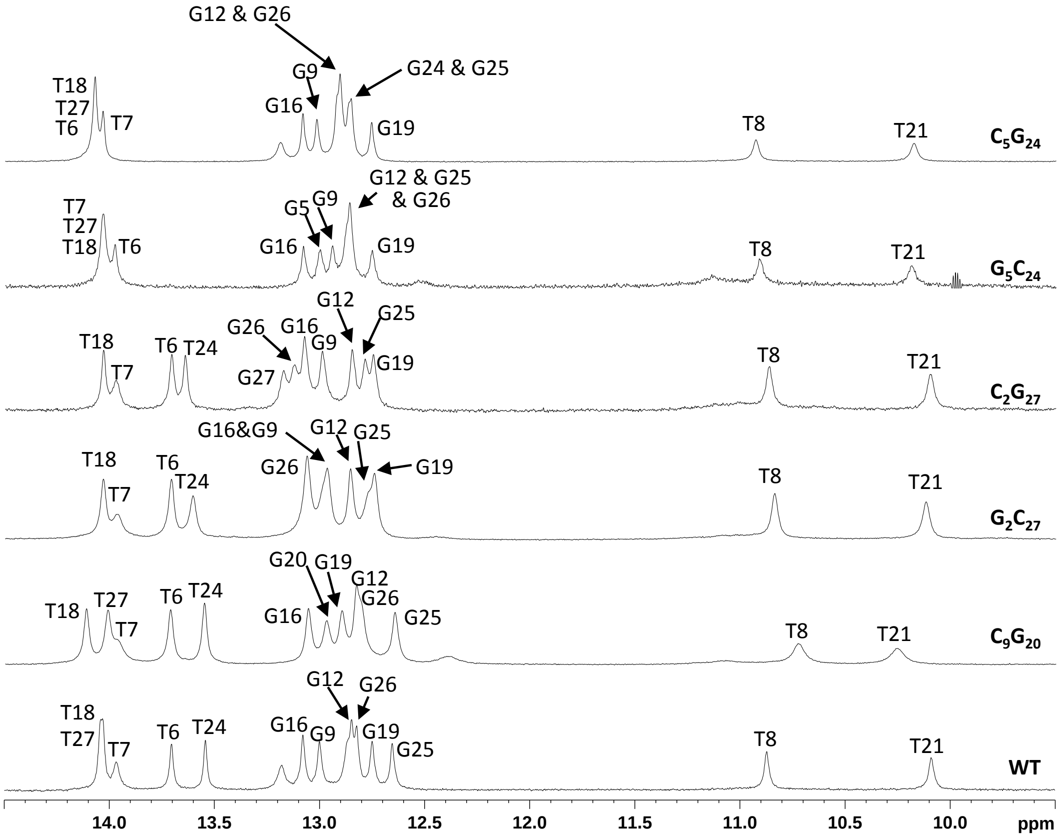

Figure S4. Overview of the ^{1}H imino region of the wild type sequence (wt) and the proposed alternative sequences. The corresponding names of the different sequences indicated next to their respective spectra refer to the base pairs altered with respect to the numbering in the original wild type sequence.

Naming conventions of figures in supplementary material

Since all data was recorded at pD 5.0 and all the pKaH-values are found to be higher than 7.7, the duplexes feature an imidazolium-group which is indicated explicitly in all subsequent figures by the use of TxImH+.

Chemical shift perturbation overview of all modified T_x^{Im} duplexes

In the following figures (S5 – S11) the chemical shift perturbation of each modified T_x^{Im} duplex with respect to the corresponding non-modified template sequence are shown, both for the exchangeable imino protons as for the non-exchangeable sugar and aromatic base protons. In each case, the spectra used for the perturbation mapping of the imino protons are measured at 5°C and pH 5.0 in H_2O/D_2O 90%/10%. In a similar fashion, the perturbation mapping of the non-exchangeable protons is performed using data recorded at 25°C and pD 5.0 in 100% D_2O.

T_6^{Im} modified duplex

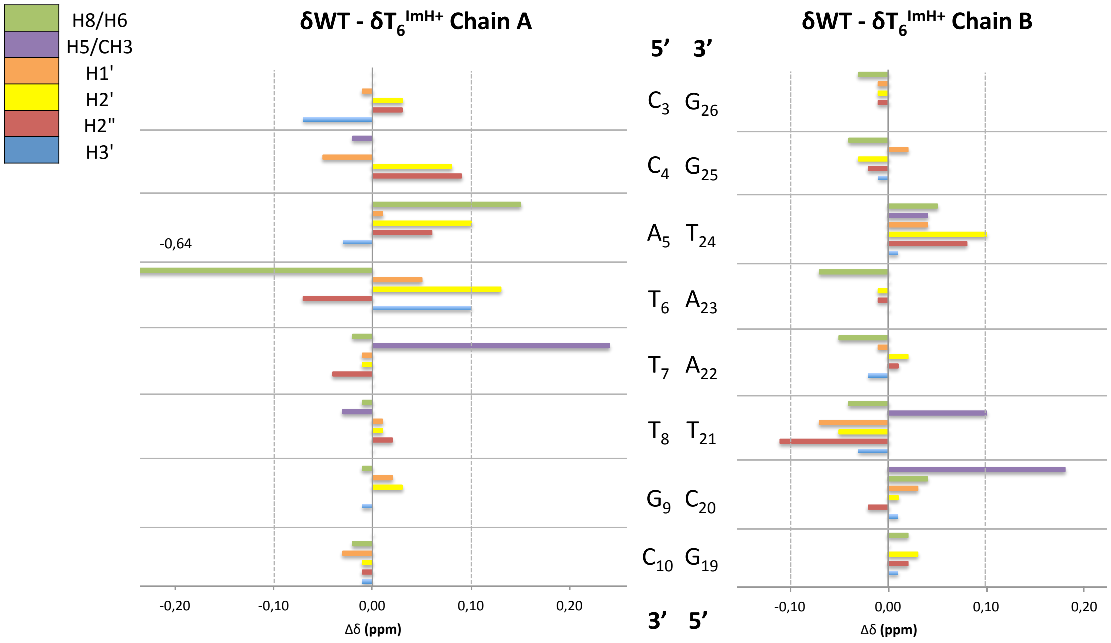

Figure S5. Chemical shift perturbation of the imino (grey, top) and non-exchangeable protons of the T_6^{Im} duplex with respect to the wild type sequence (bottom).

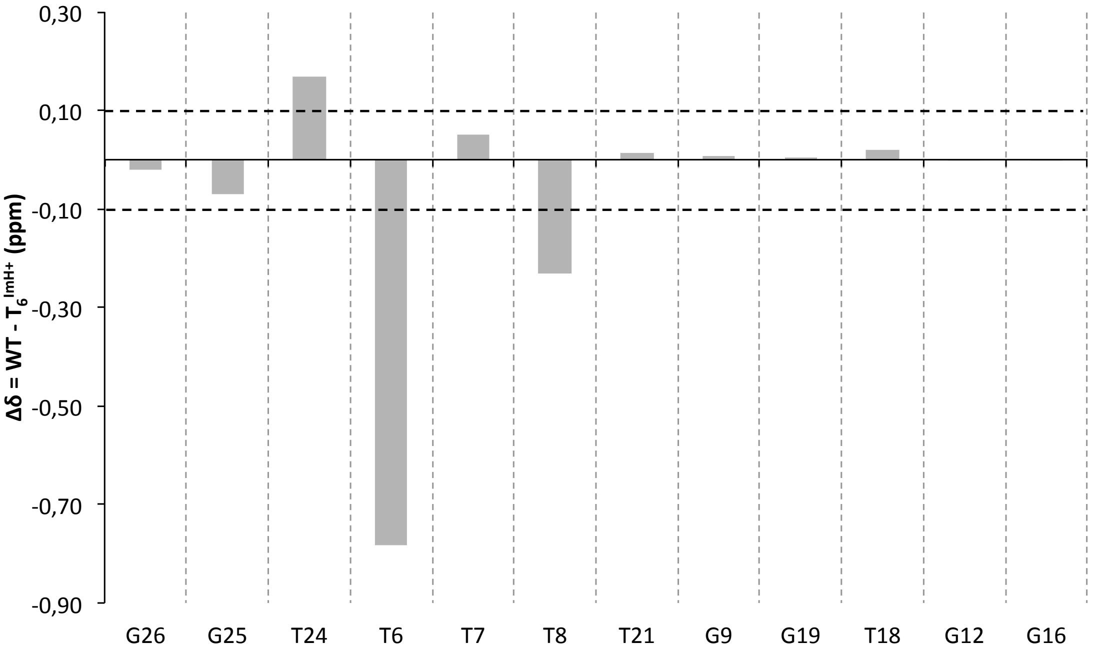

T_7^{Im} modified duplex

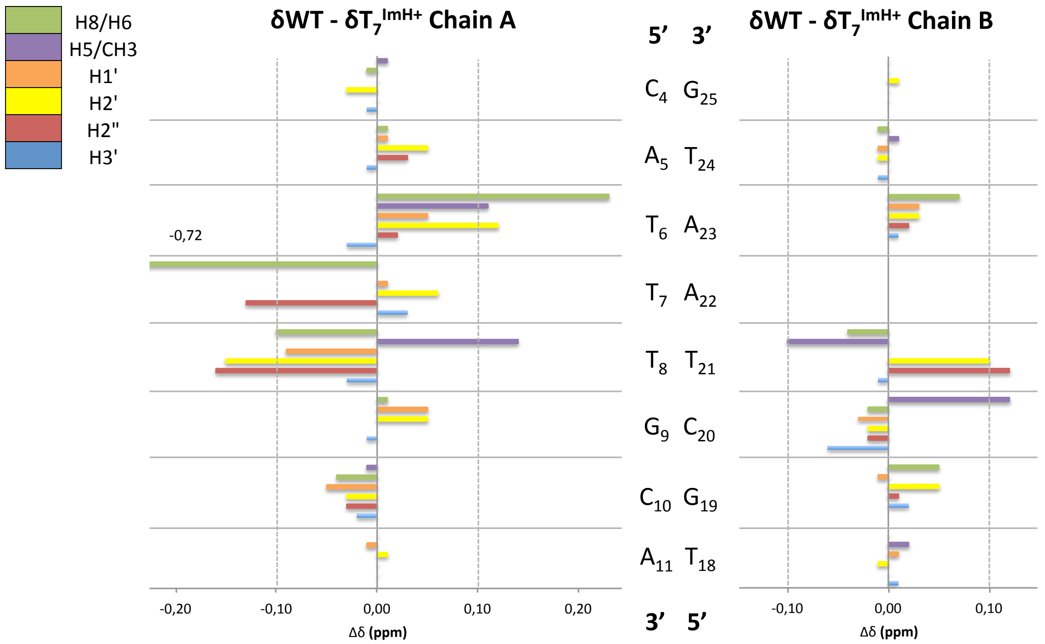

Figure S6. Chemical shift perturbation of the imino (grey, top) and non-exchangeable protons of the T_7^{Im} duplex with respect to the wild type sequence (bottom).

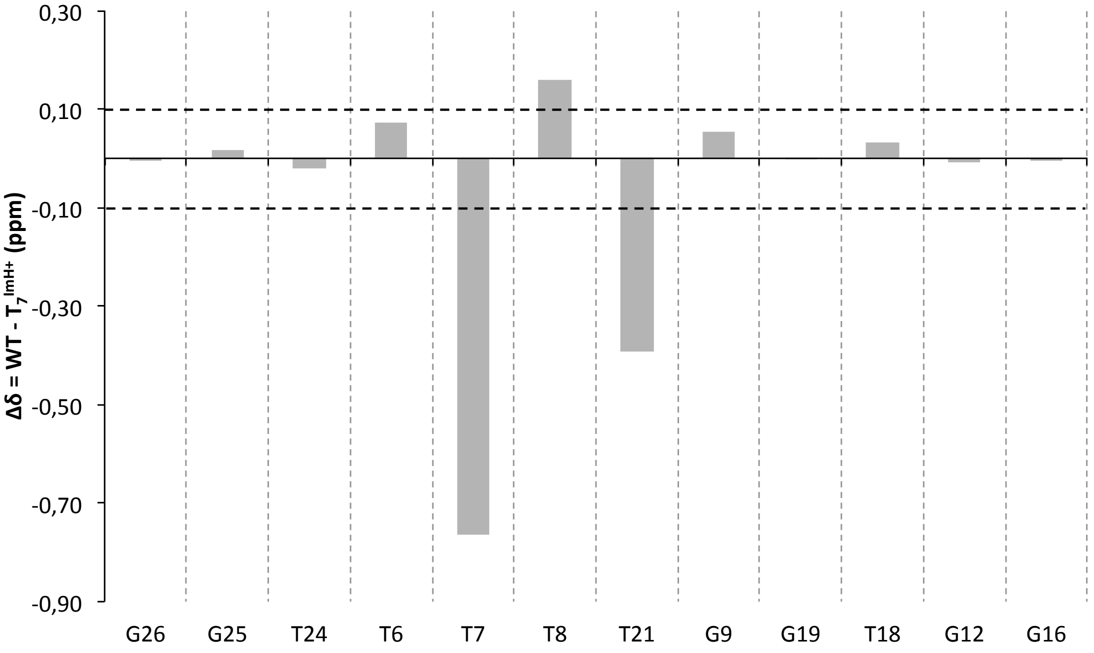

T_{21}^{Im} modified duplex

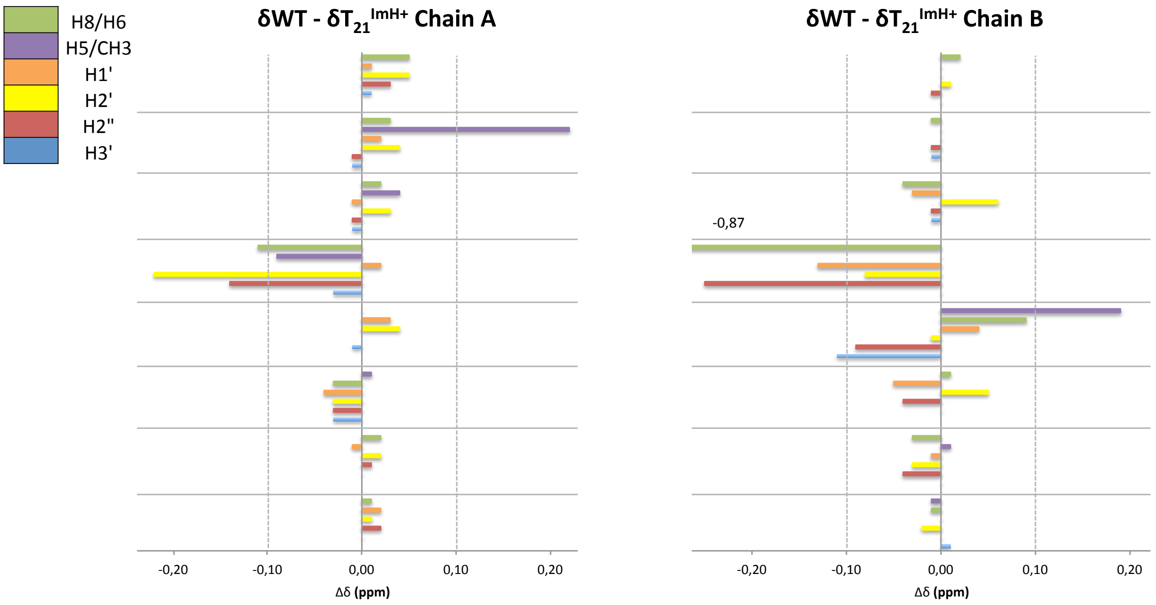

Figure S7. Chemical shift perturbation of the imino (grey, top) and non-exchangeable protons of the T_{21}^{Im} duplex with respect to the wild type sequence (bottom).

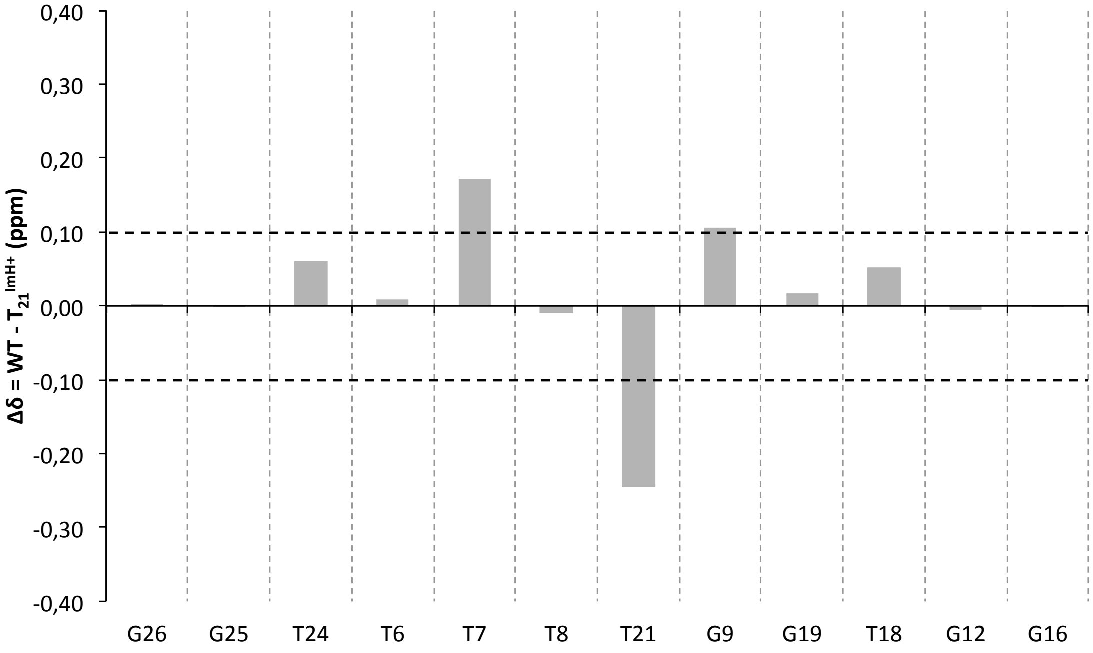

T_8^{Im} modified duplex

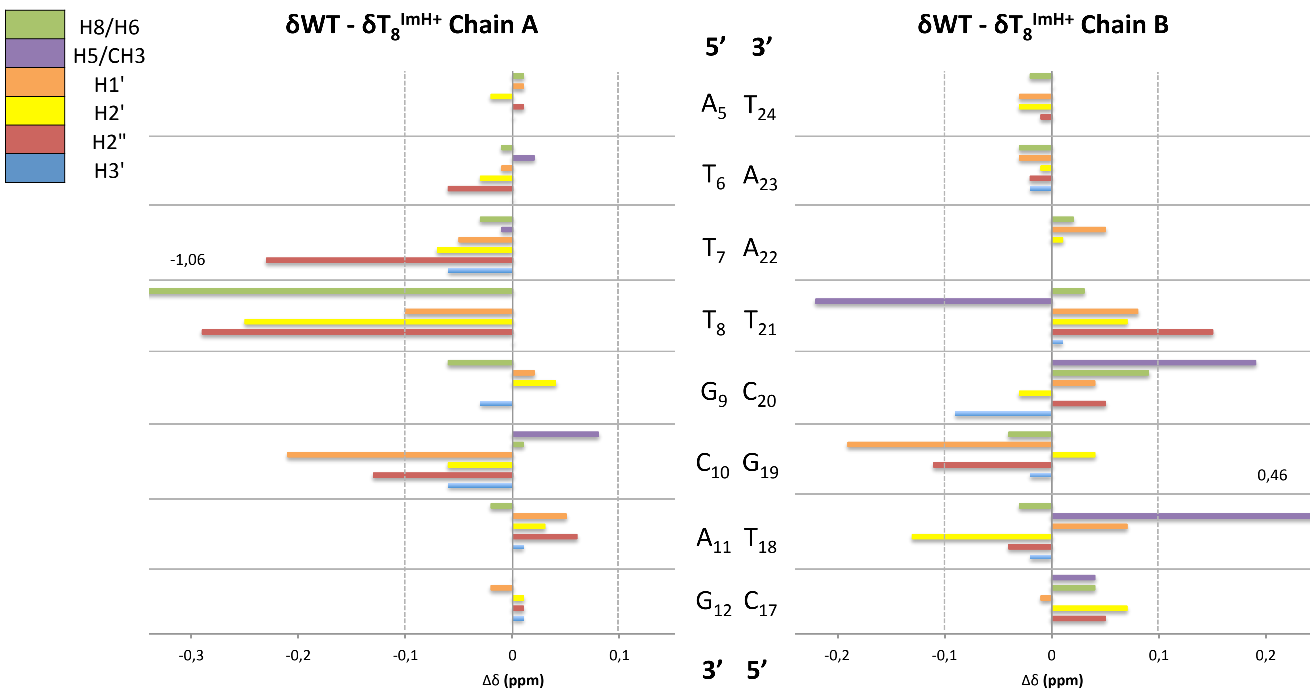

Figure S8. Chemical shift perturbation of the imino (grey, top) and non-exchangeable protons of the T_8^{Im} duplex with respect to the wild type sequence (bottom).

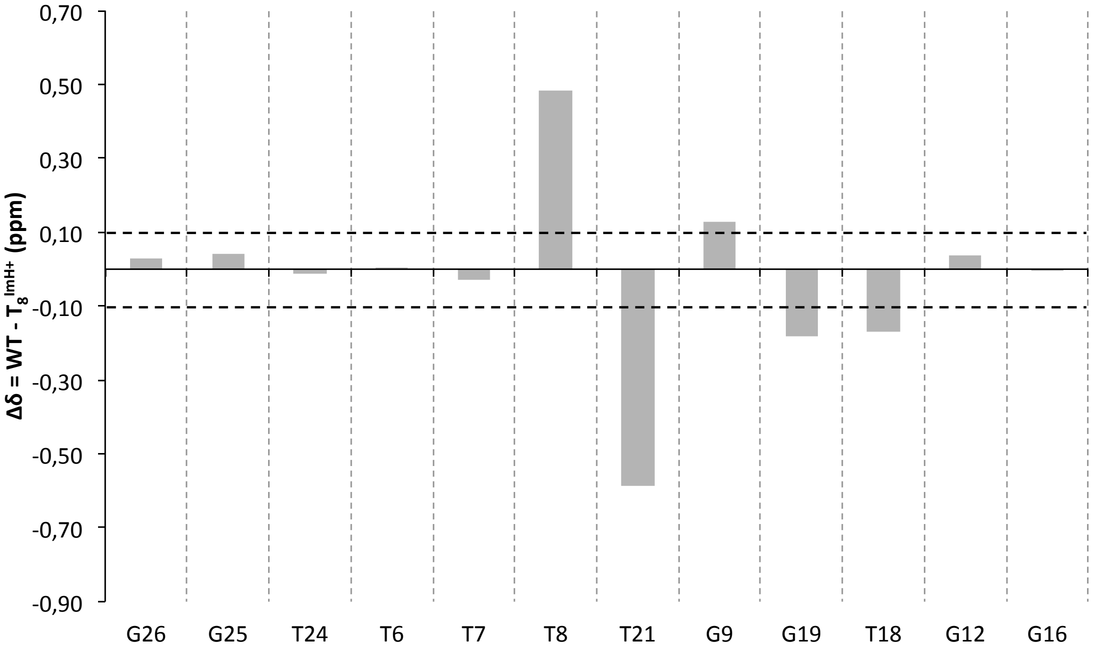

$T_8^{Im}A_{21}$ modified duplex

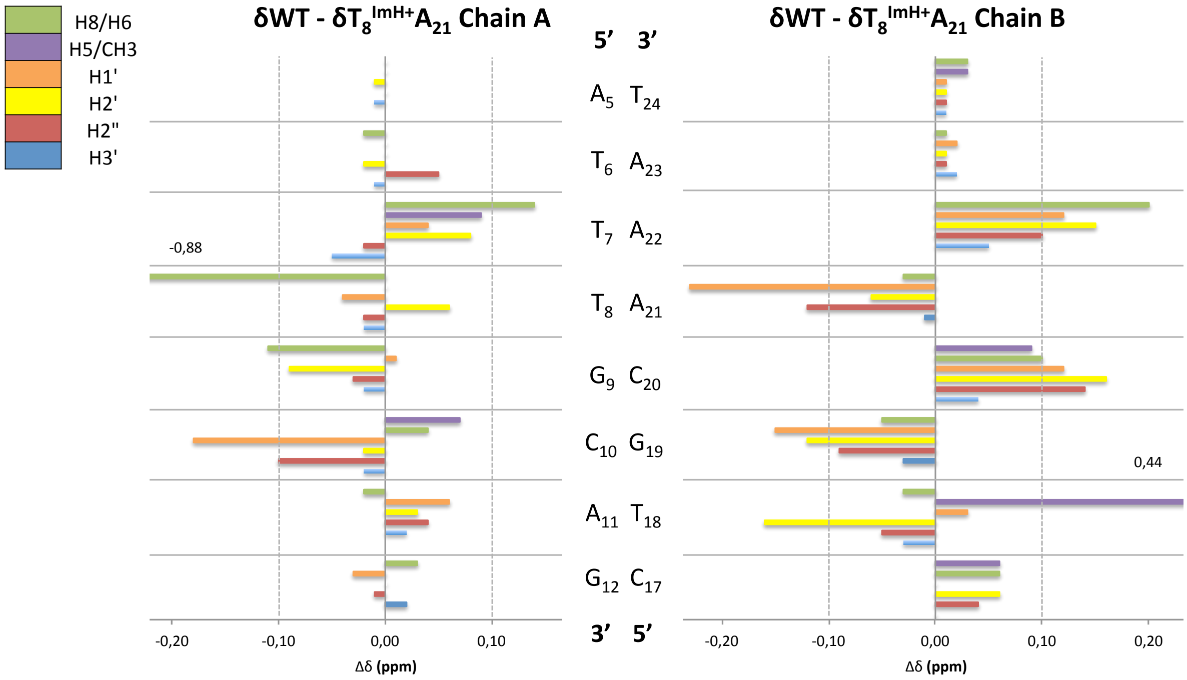

Figure S9. Chemical shift perturbation of the imino (grey, top) and non-exchangeable protons of the $T_8^{Im}A21$ duplex with respect to the T8A21 wild type sequence (bottom).

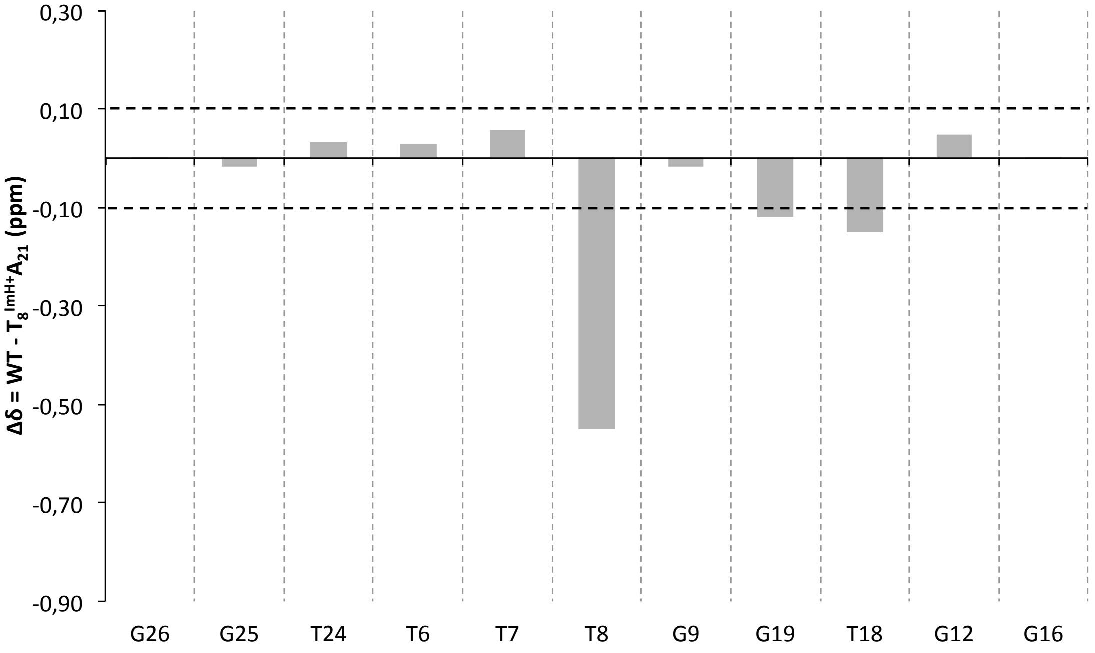

sT_{21}^{Im} modified duplex

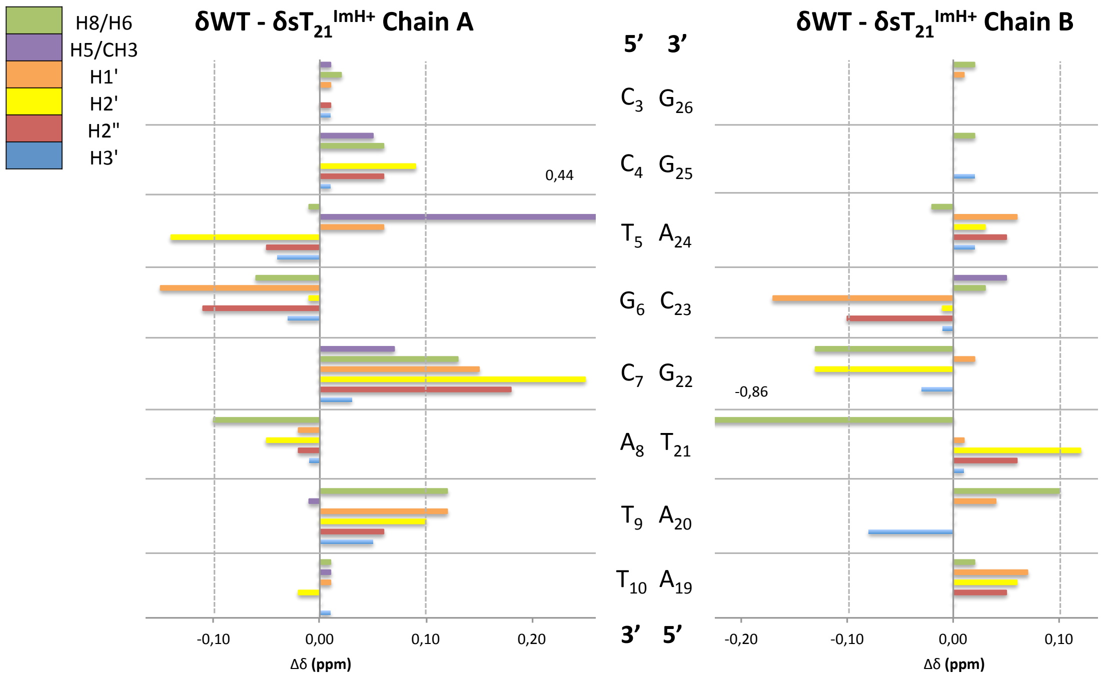

Figure S10. Chemical shift perturbation of the imino (grey, top) and non-exchangeable protons of the sT_{21}^{Im} duplex with respect to the sT_{21} wild type sequence (bottom).

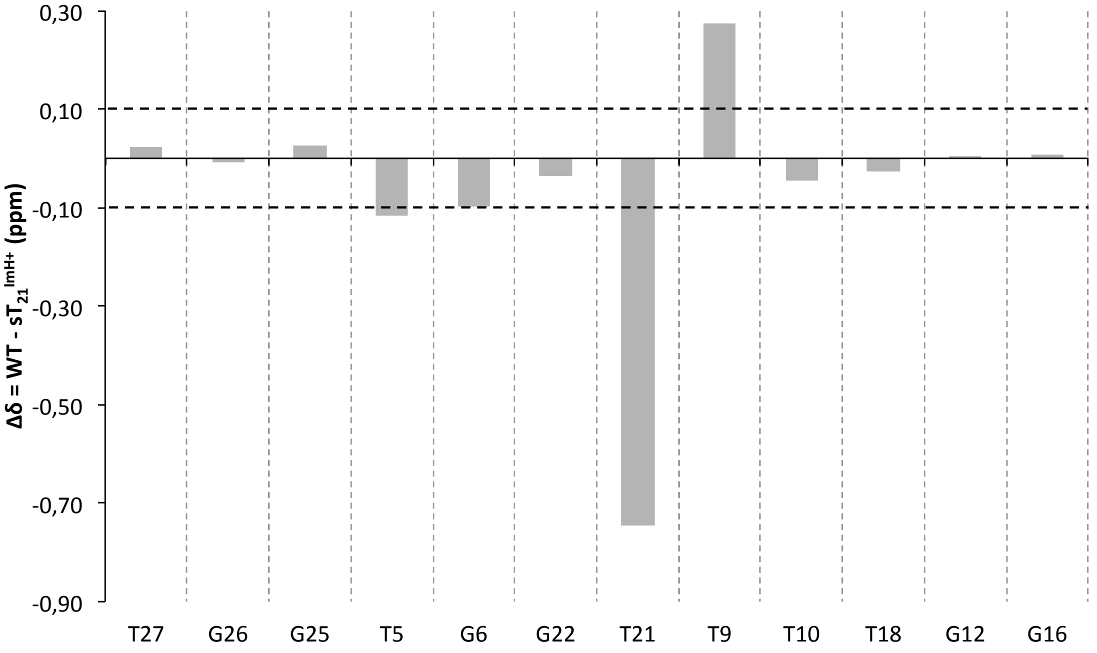

$A_8T_{21}^{Im}$ modified duplex

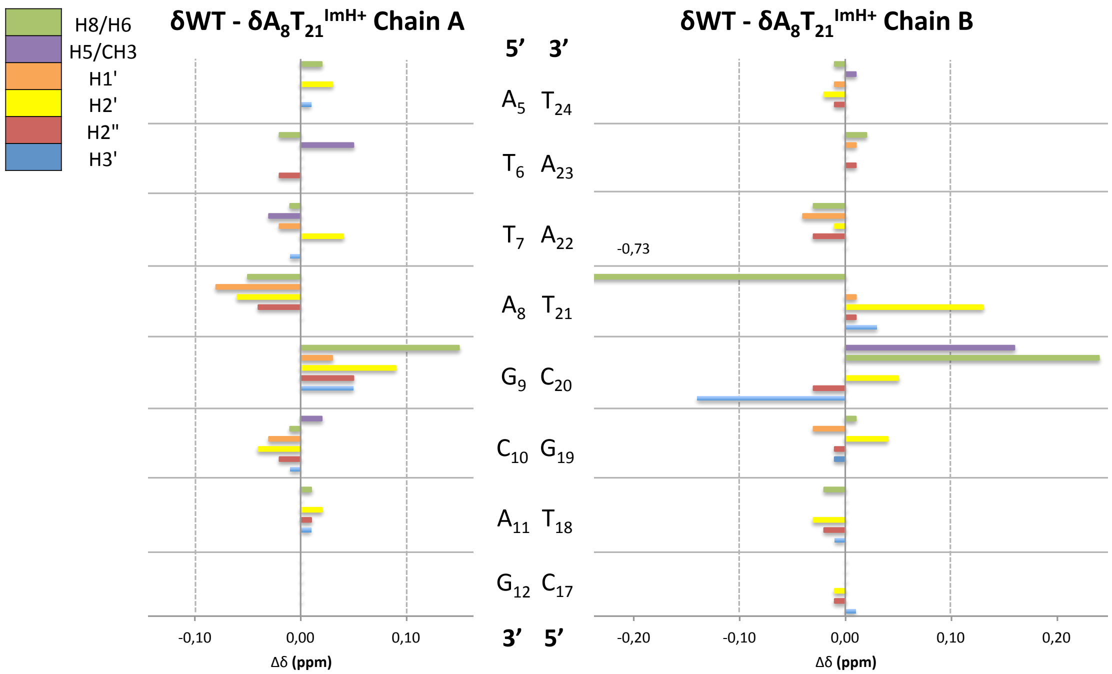

Figure S11. Chemical shift perturbation of the imino (grey, top) and non-exchangeable protons of the $A_8T_{21}^{Im}$ duplex with respect to the A_8T_{21} wild type sequence (bottom).

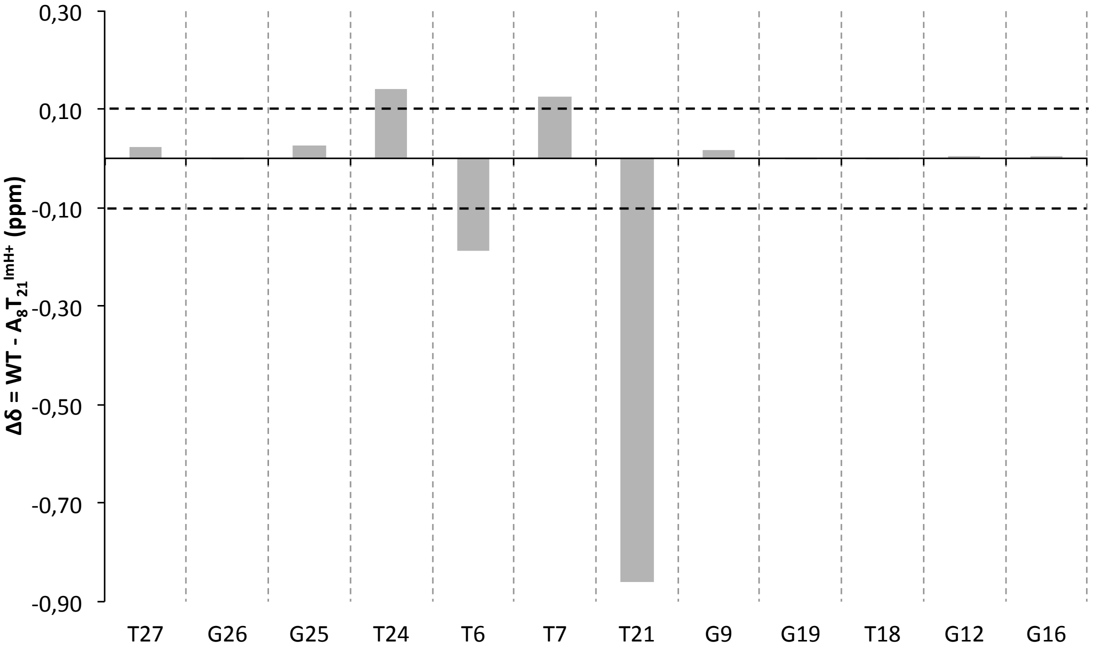

Pka_H-regulating motif in $T_8^{Im}A_{21}$ and sT_{21}^{Im}

In figure S12 – S13, structural snapshots corresponding with the most persistent hydrogen bond conformations from the molecular dynamics run of both the $T_8^{Im}A_{21}$ and sT_{21}^{Im} duplexes are shown

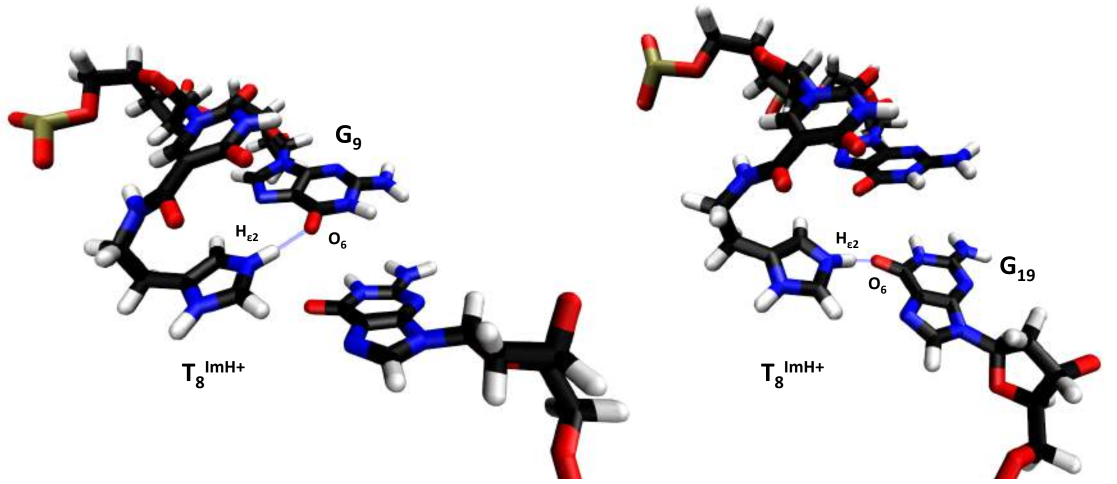

Figure S12. Main conformations with an intact hydrogen bond motif for the $T_8^{Im}A_{21}$ duplex. In both cases the carbonyl O_6 atom from G_9 and G_{19} respectively are the main hydrogen bond acceptors and Hε1 of the modified T_8^{ImH+} base the hydrogen bond donor.

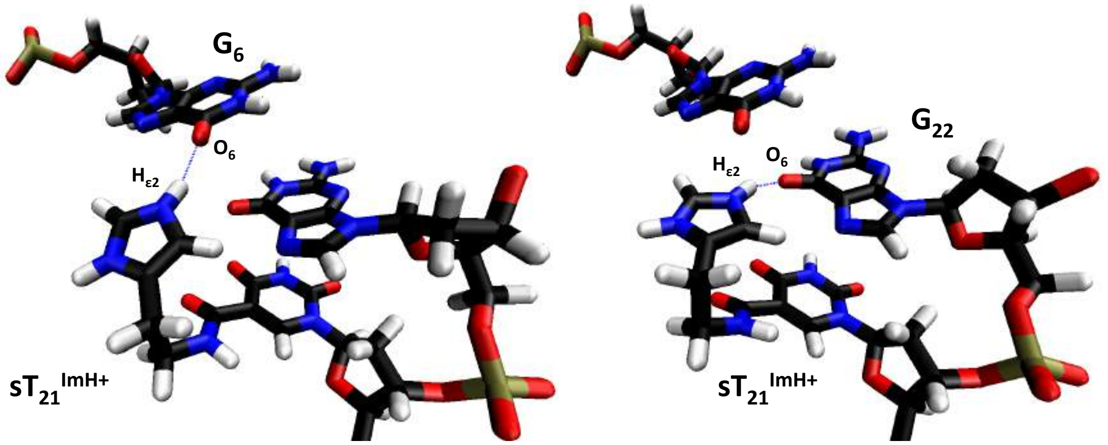

Figure S13. Main conformations with an intact hydrogen bond motif for the sT_{21}^{Im} duplex. In both cases the carbonyl O_6 atom from G_9 and G_{19} respectively are the main hydrogen bond acceptors and Hε1 of the modified T_{21}^{ImH+} base the hydrogen bond donor.

Validation molecular dynamics trajectory: wild type and T_8^{lm}

During the trajectory analysis, both 5' and 3' outer ends of each DNA strand will not be taken into account since these ends have a tendency to fraying and hence are prone to show a-typical conformational behaviour. In addition, when a T-T mismatch is present in the DNA duplex, no preferential wobble base pair is imposed on the starting structure, meaning at the start of every trajectory, the system is free to choose which wobble conformation it will adopt.

Considering the sampling of the conformational α/γ space, Várnai and Hartmann have shown that three combinations of α/γ correspond to local energy minima: g+/g- (canonical), g-/t and g+/t in their respective order of decreasing stability for free B-DNA structures. Other non-canonical α/γ conformations are predominantly found in protein-DNA complexes and hence represent higher-energy states, which are not freely accessible for DNA in solution.

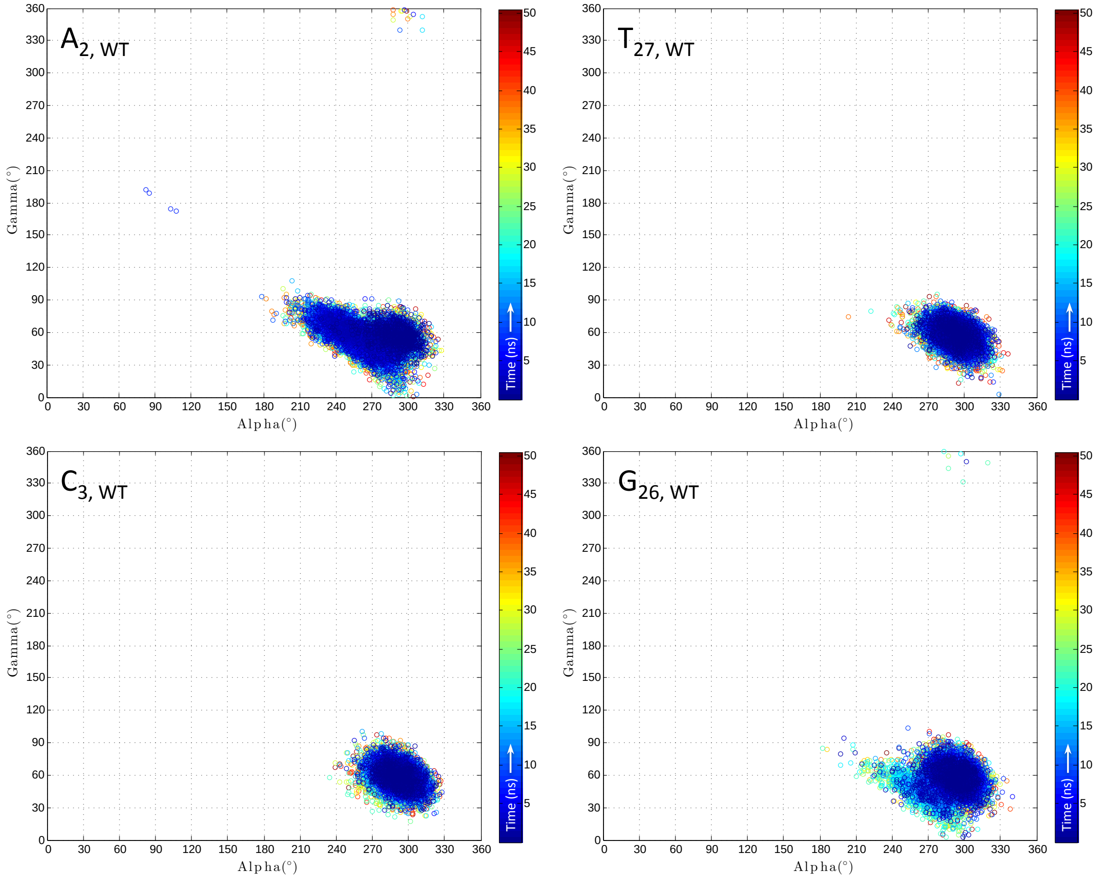

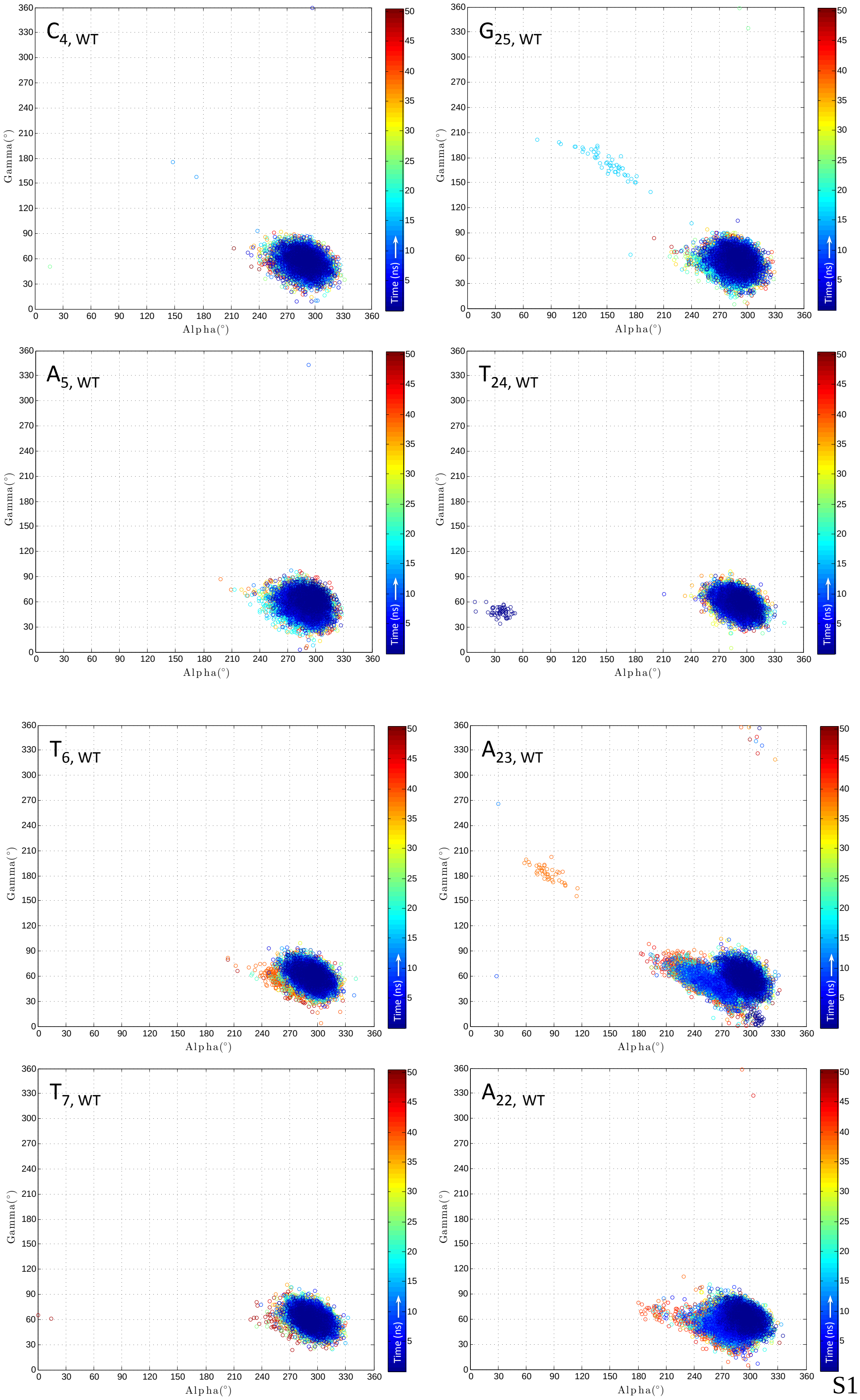
C4, WT
G25, WT
A5, WT
T24, WT
T6, WT
A23, WT
T7, WT
A22, WT
Gamma(°)
Alpha(°)
Time (ns)

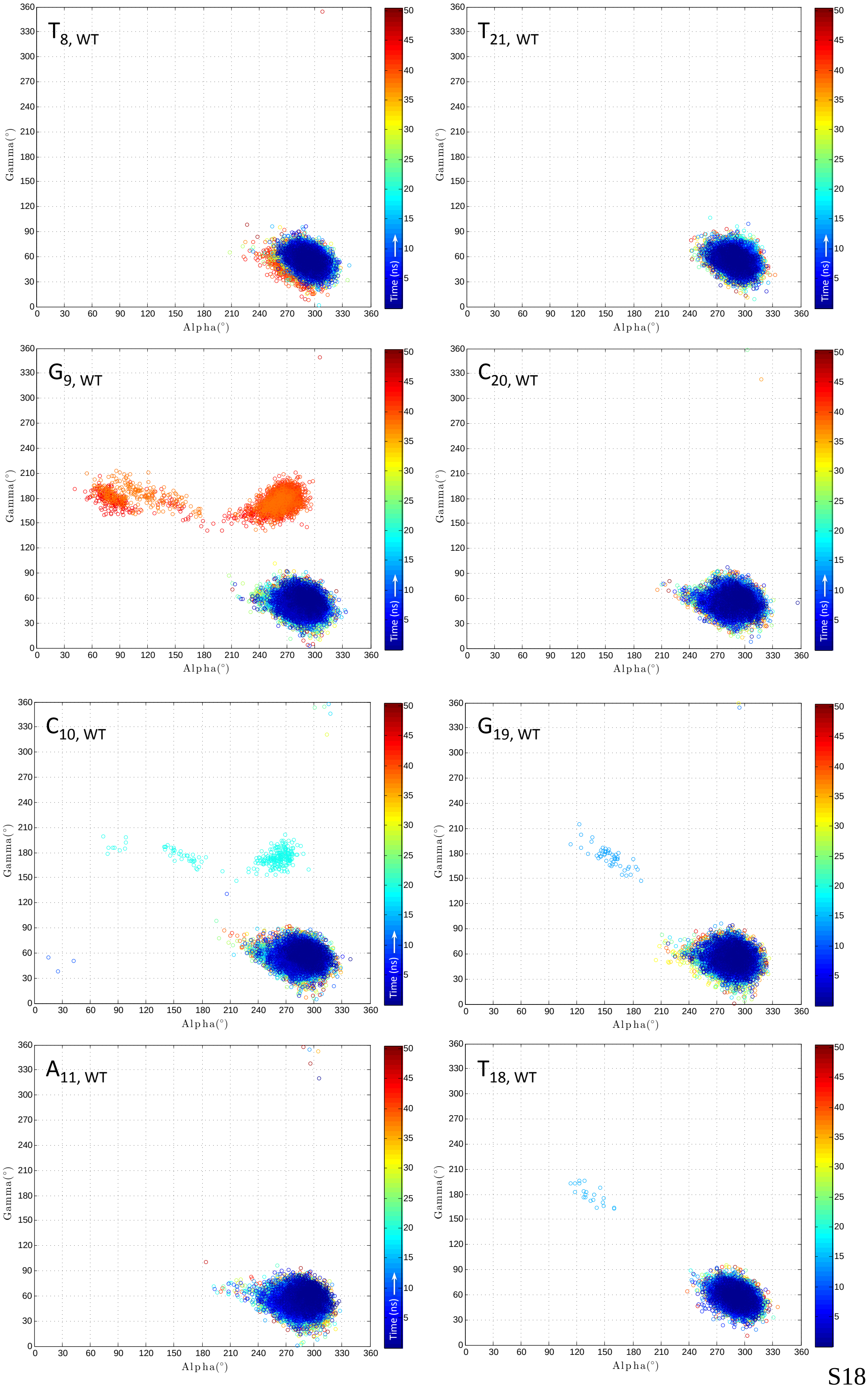

T8, WT
T21, WT
G9, WT
C20, WT
C10, WT
G19, WT
A11, WT
T18, WT
Gamma(°)
Alpha(°)
Time (ns)
0 30 60 90 120 150 180 210 240 270 300 330 360
5 10 15 20 25 30 35 40 45 50

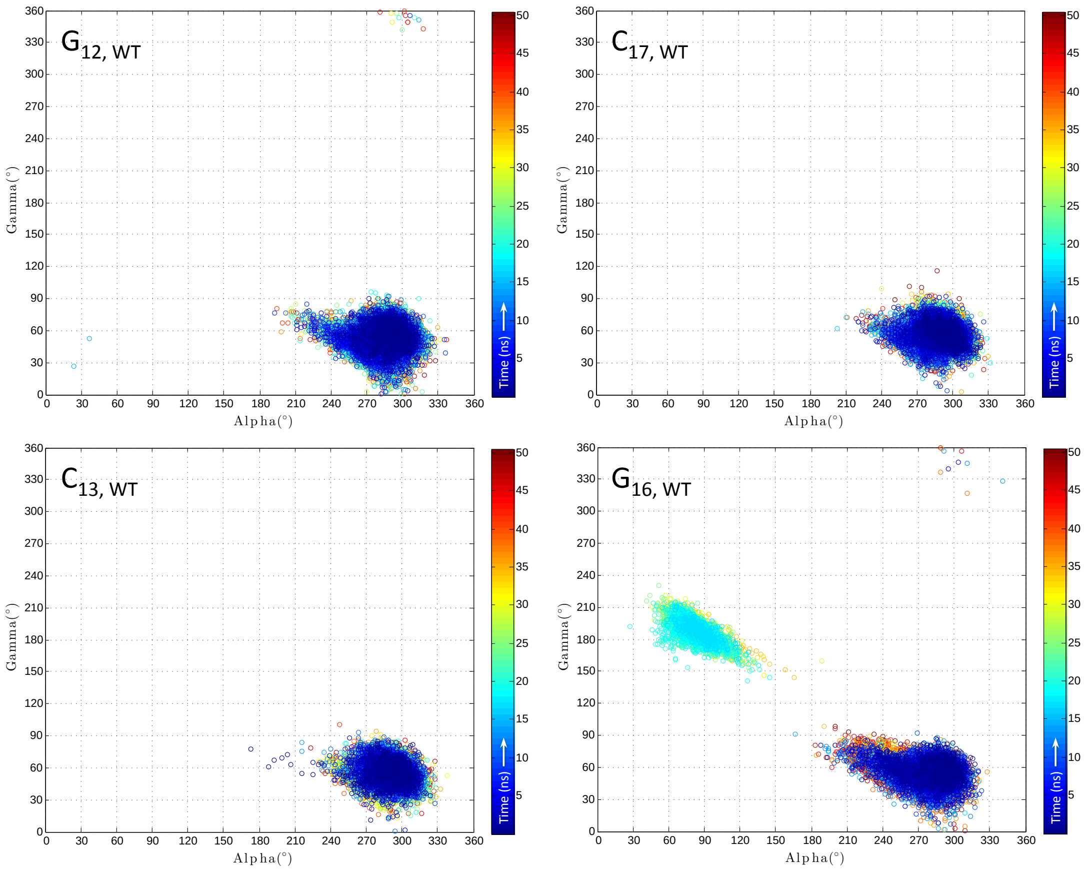

Figure S14. α/γ sampling of the equilibration and production molecular dynamics trajectory of the wild type duplex for a total period of 50,120ns. Each data point corresponds with a snapshot every 2ps. All base pairs are shown except for the 5’ and 3’ ends.

For the wild type sequence, it may be clear that for all the relevant base pairs, the majority of the conformations are located in the $\alpha/\gamma \approx g^-/g^+ \approx$ (300°, 30°) energy minimum. In addition, the G_9 base also samples both the g^+/t and g^-/t local minima, characterized by higher-energy than the global minima, near the end of the trajectory. To a minor extent, G_{16} also shows this behaviour although here a higher mobility is not unexpected due to the close presence to one of the duplex ends.

This observation by and large remains true for the same base pairs of the T_8^{lm}modified duplex, with two main differences for T_7 and G_9. First off, where T_7 in the wild type sequence only populates the g^+/g^- global minimum, the g^-/t local minimum is now also significantly populated. Furthermore this transition is reversible. With G_9 as well, a reversible transition from g^-/g^+ to g^+/t for a short duration of time can be observed. In addition, where with G_9 in the wild type sequence both local minima are sampled, this is no longer the case for G_9 in T_8^{lm}, during the simulated time window. Interestingly enough, no significant differences can be observed for T_8 of T_8^{lm} duplex with respect to T_8 base in the wild type sequence.

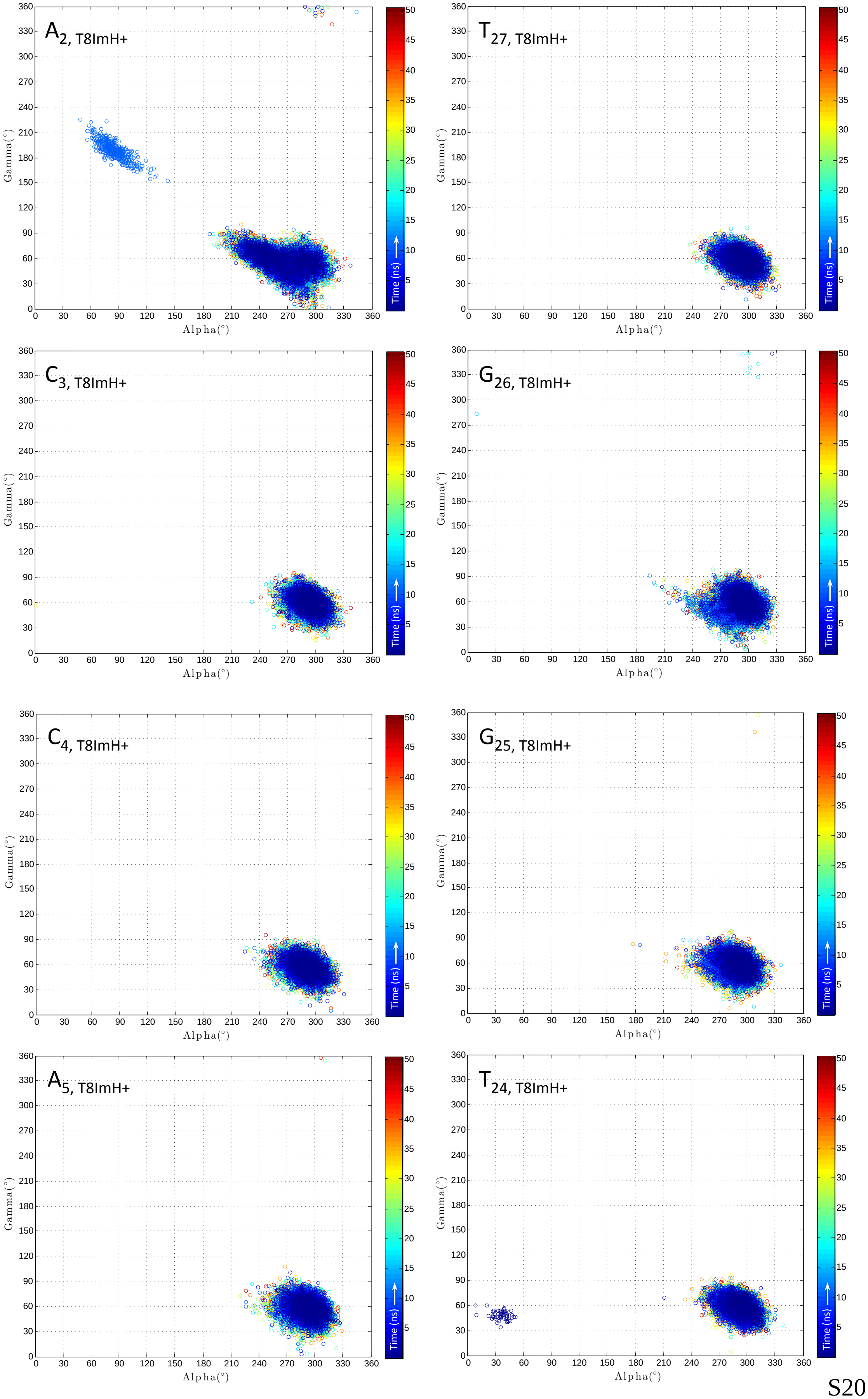
A2, T8ImH+
T27, T8ImH+
C3, T8ImH+
G26, T8ImH+
C4, T8ImH+
G25, T8ImH+
A5, T8ImH+
T24, T8ImH+
Gamma(°)
Alpha(°)
Time (ns)

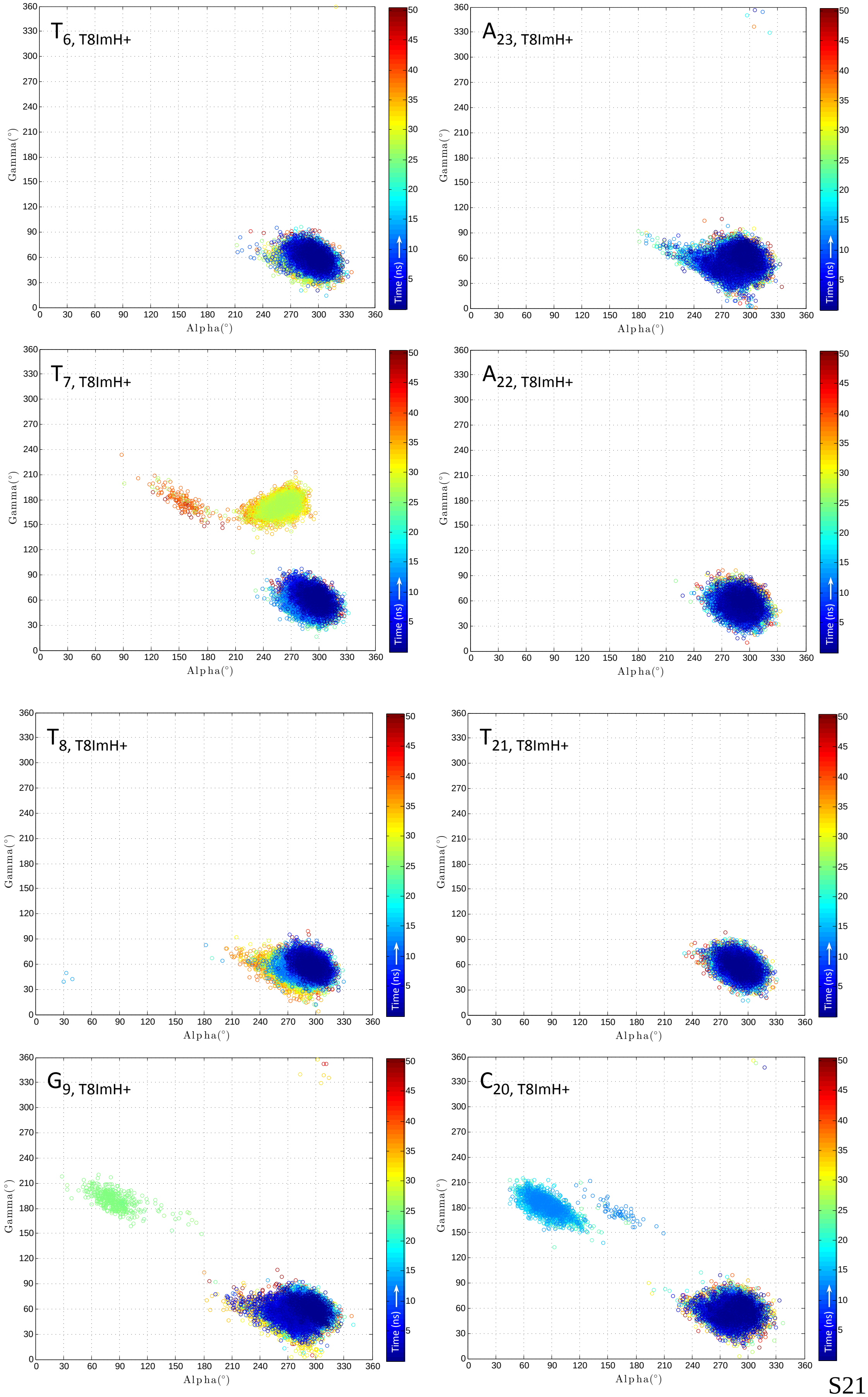
T6, T8ImH+
A23, T8ImH+
T7, T8ImH+
A22, T8ImH+
T8, T8ImH+
T21, T8ImH+
G9, T8ImH+
C20, T8ImH+
Gamma(°)
Alpha(°)
Time (ns)

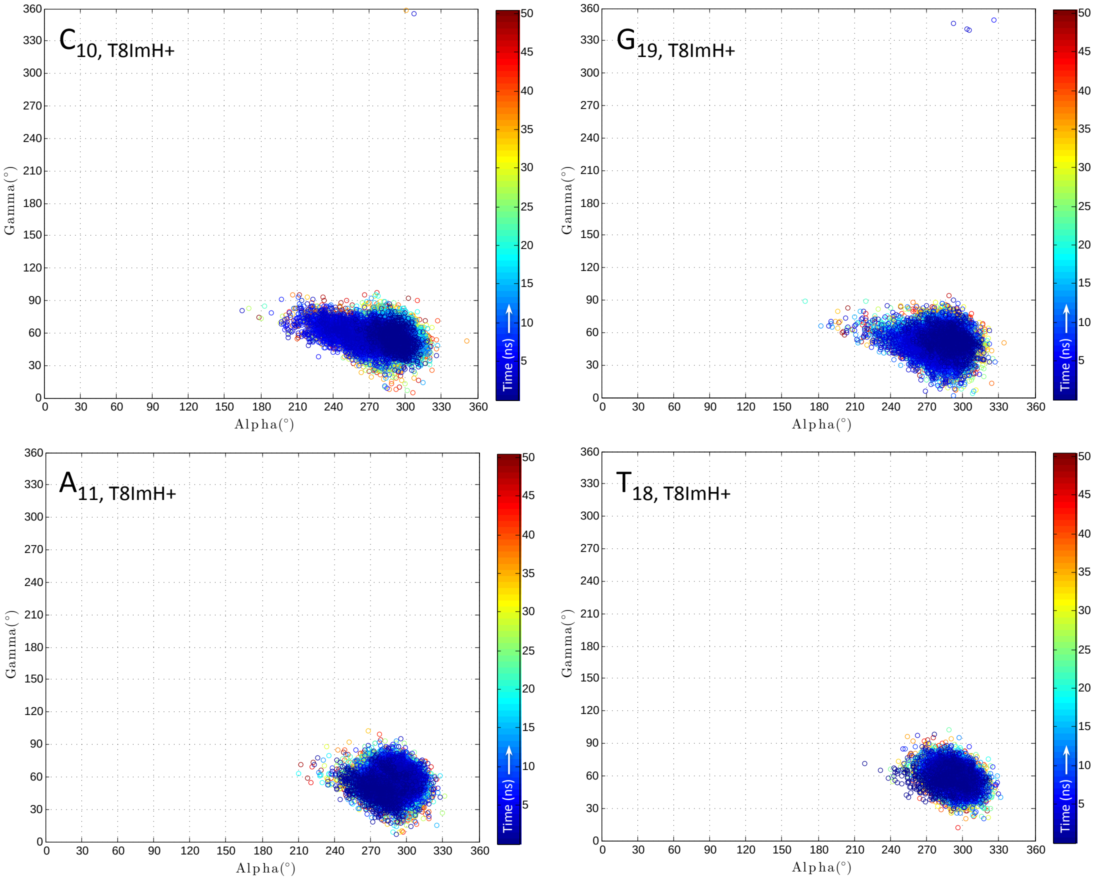
C10, T8ImH+
G19, T8ImH+
A11, T8ImH+
T18, T8ImH+
Gamma(°)
Alpha(°)
Time (ns)

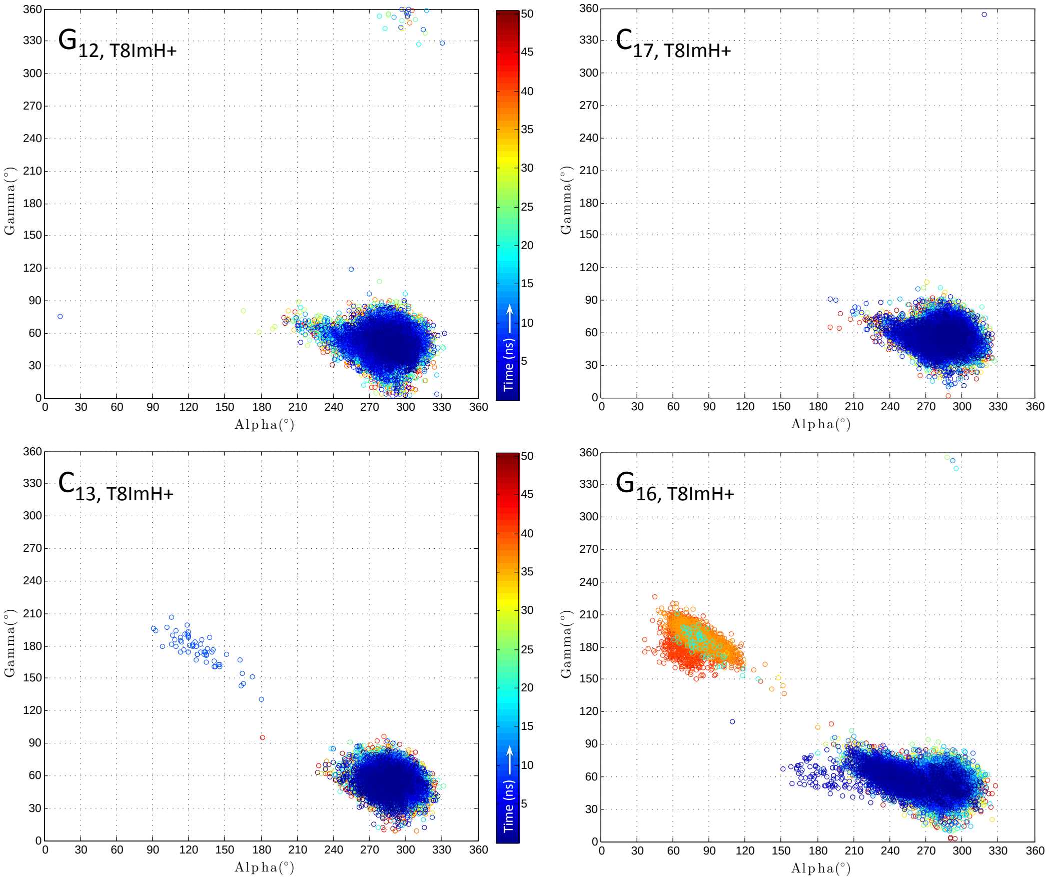

Figure S15. α/γ sampling of the equilibration and production molecular dynamics trajectory of the T_8^{Im} duplex for a total period of 50,120ns. Each data point corresponds with a snapshot every 2ps. All base pairs are shown except for the 5’ and 3’ ends.

Secondly, the B_I/B_{II} conformations control the phosphate positions, influence groove parameters, stacking properties and helicoidal parameters. In general, the lower energy B_I state is characterized by ε/ζ: t/g with ε-ζ ≈ -90° and is predominantly found in the canonical B-DNA duplex. B_{II} on the other hand can be identified by ε/ζ: g⁻/t with ε-ζ ≈ +90° and is slightly less favoured. The angular histograms shown in figure. S16 – S17 show for all nucleotides from both the wild type and T_8^{Im} duplex the corresponding B_I/B_{II} torsional parameter (ε-ζ). Concerning the wild type, every nucleotide resides for the majority of the trajectory in the B_I conformation. Only G_{19} populates for a small but significant time period the B_{II} conformation.

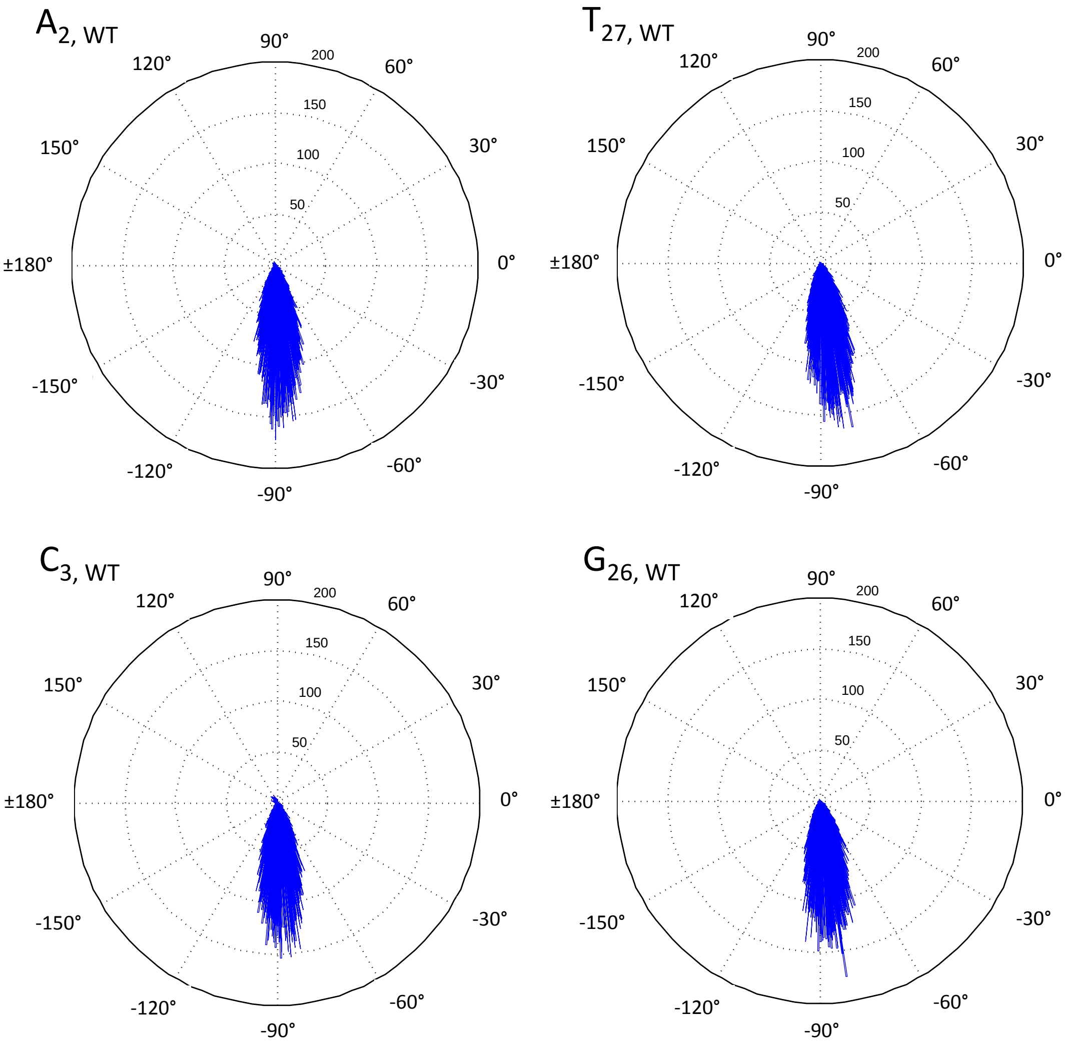

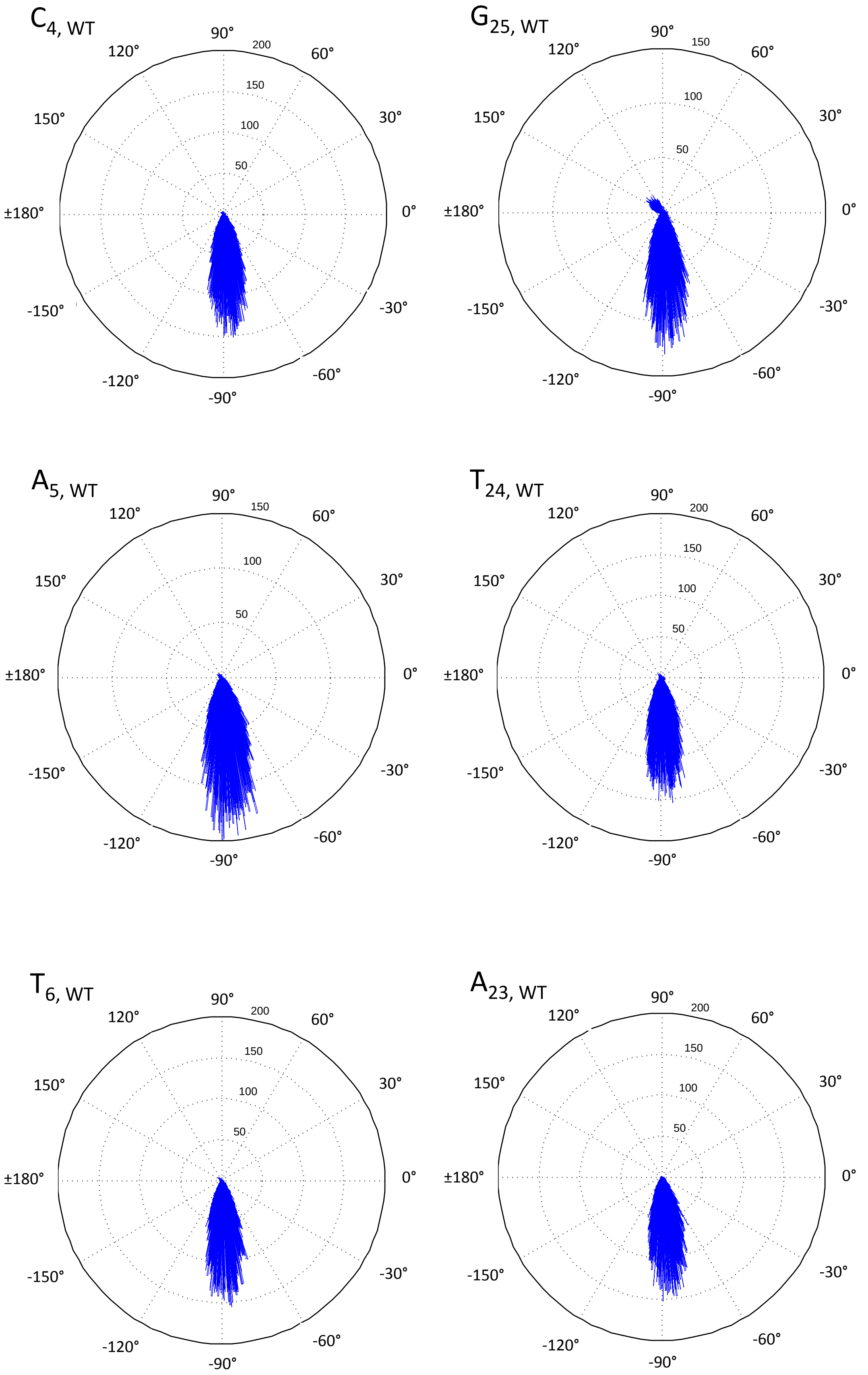

C4, WT
G25, WT
A5, WT
T24, WT
T6, WT
A23, WT
90°
60°
30°
0°
-30°
-60°
-90°
-120°
-150°
±180°
150°
120°
50
100
150
200

$T_{7, WT}$

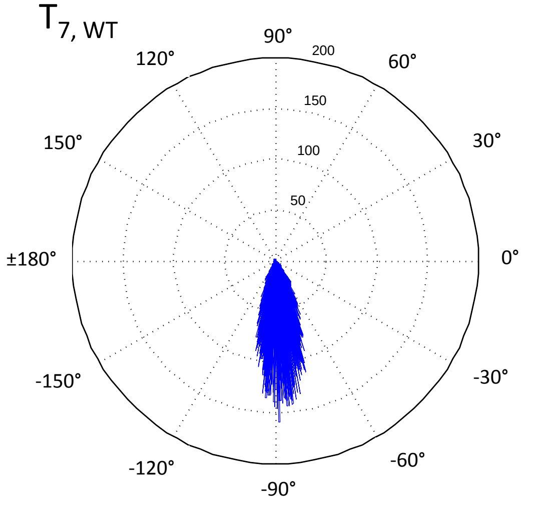

$A_{22, WT}$

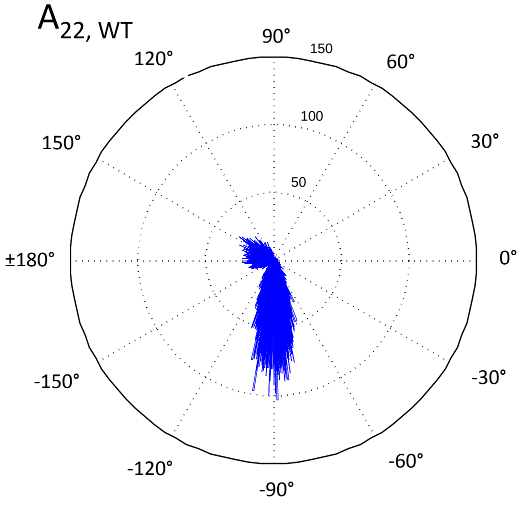

$T_{8, WT}$

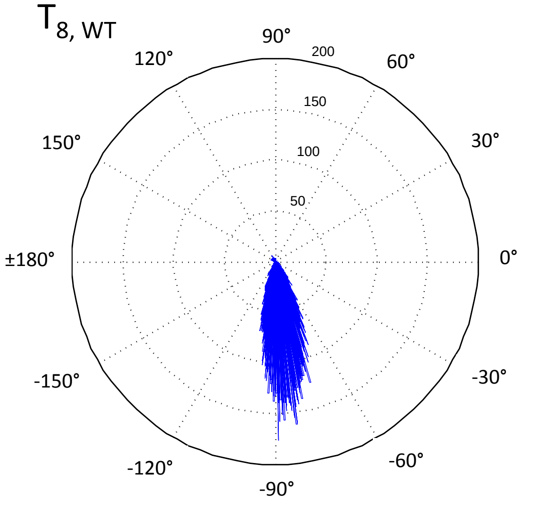

$T_{21, WT}$

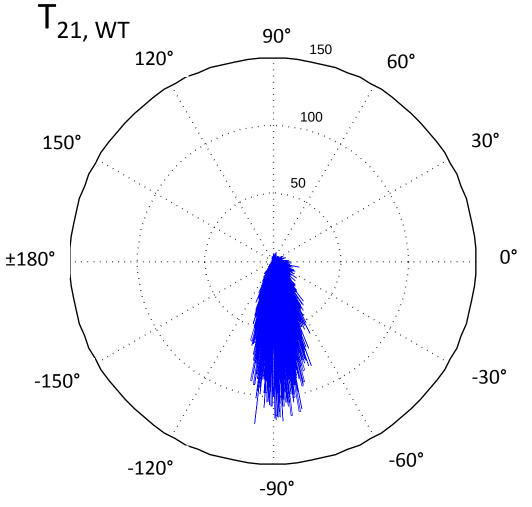

$G_{9, WT}$

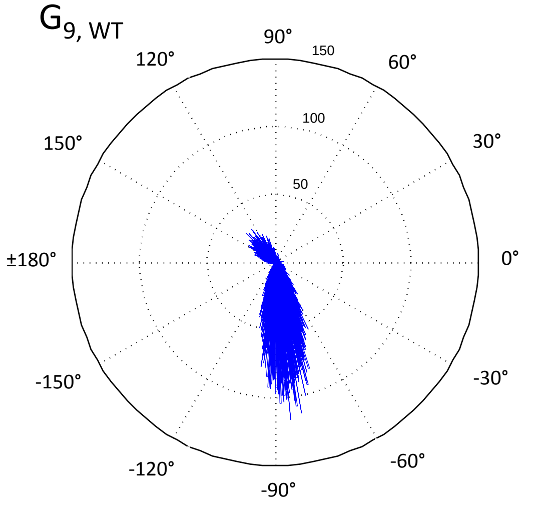

$C_{20, WT}$

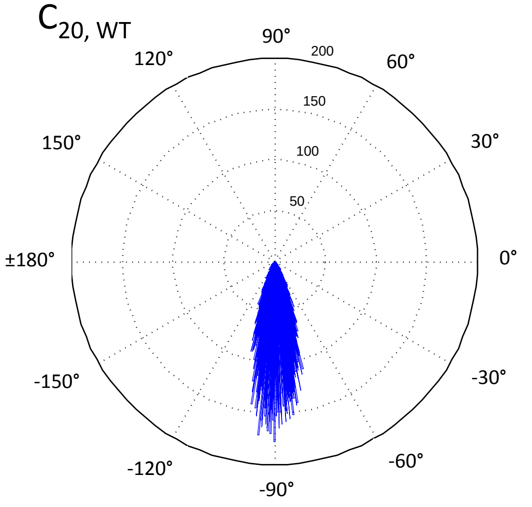

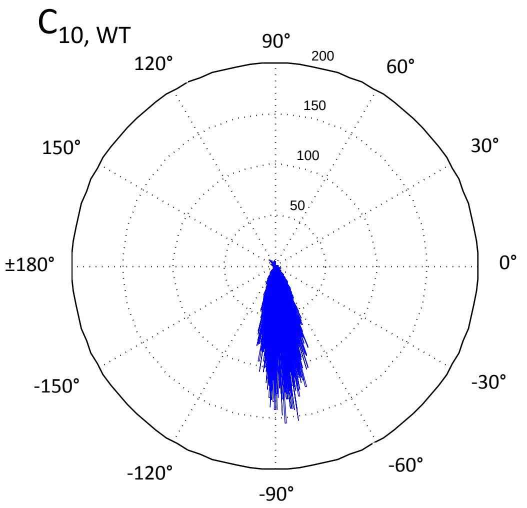
C_{10}, WT
90°
60°
30°
0°
-30°
-60°
-90°
-120°
-150°
±180°
150°
120°
200
150
100
50

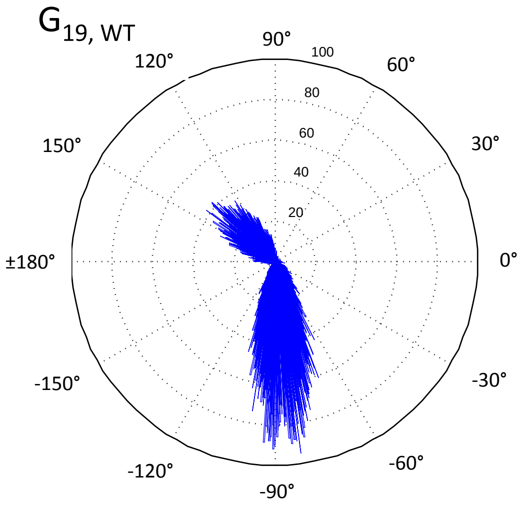
G_{19}, WT
90°
60°
30°
0°
-30°
-60°
-90°
-120°
-150°
±180°
150°
120°
100
80
60
40
20

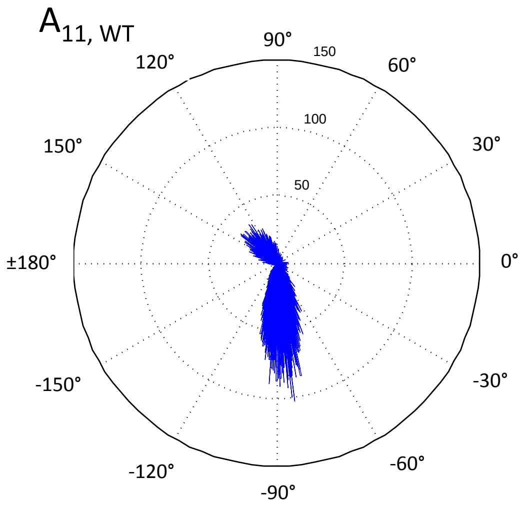
A_{11}, WT
90°
60°
30°
0°
-30°
-60°
-90°
-120°
-150°
±180°
150°
120°
150
100
50

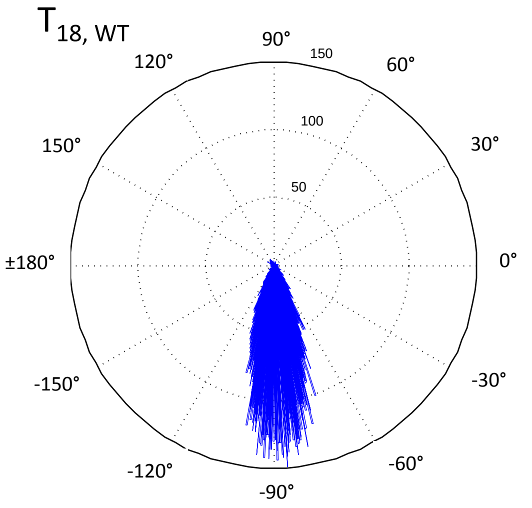
T_{18}, WT
90°
60°
30°
0°
-30°
-60°
-90°
-120°
-150°
±180°
150°
120°
150
100
50

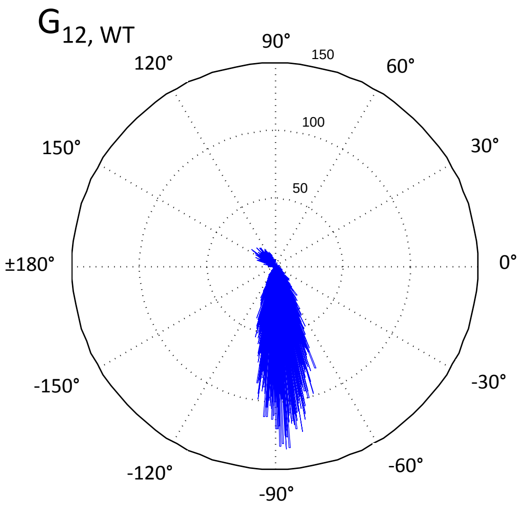
G_{12}, WT
90°
60°
30°
0°
-30°
-60°
-90°
-120°
-150°
±180°
150°
120°
150
100
50

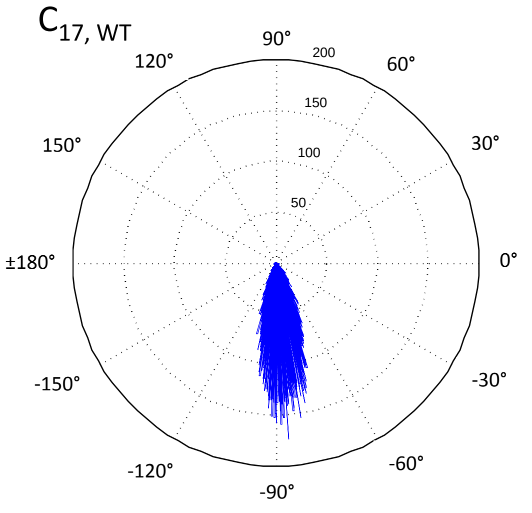
C_{17}, WT
90°
60°
30°
0°
-30°
-60°
-90°
-120°
-150°
±180°
150°
120°
200
150
100
50

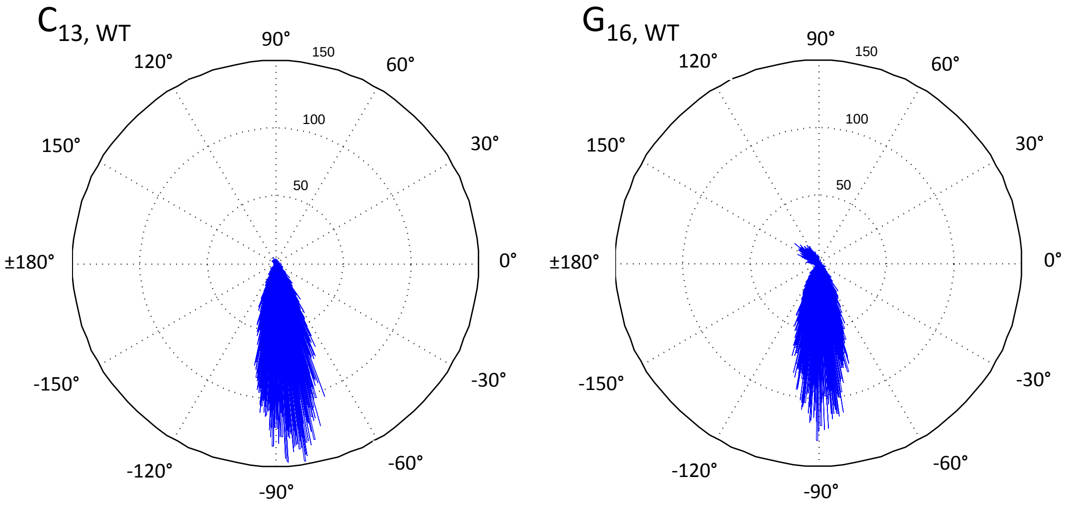

Figure S16. B_I/B_{II} torsional parameter (ε-ζ) sampling of the equilibration and production molecular dynamics trajectory of the wild type duplex for a total period of 50,120ns. Each data point corresponds with a snapshot every 2ps. All base pairs are shown except for the 5' and 3' ends.

In the same fashion figure S17 shows the B_I/B_{II} torsional parameter (ε-ζ) for the T_8^{Im} duplex. At first glance, there are no evident changes in behaviour with the exception of the G_{19} base where for the majority of the simulation time the B_{II} state is populated. In addition but to a lesser extent, G_9 now also shows a bigger sampling of the B_{II} conformation. Considering that $G_{19,}$ complemented with $G_{9,}$ is the main interaction site for the Im modificiation in T_8^{Im}, some distortion of the conformational space is to be expected. With respect to the T_8 modified base itself, it's clear that there's no sampling of B_{II} although some regions of the conformational space unknown to conventional DNA duplexes are populated as well. Considering the unique nature of the duplexes at hand, this behaviour is not entirely unexpected.

A_{2}, T8ImH+

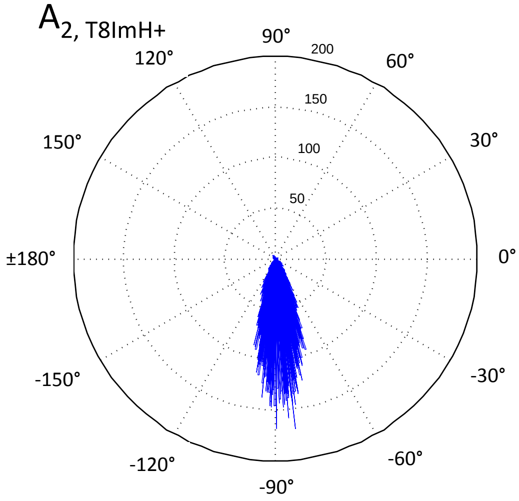

T_{27}, T8ImH+

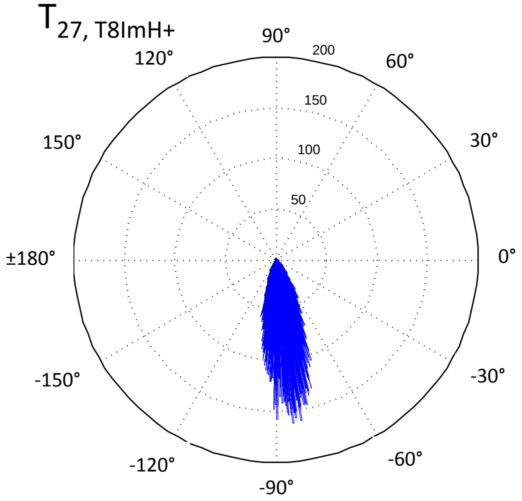

C_{3}, T8ImH+

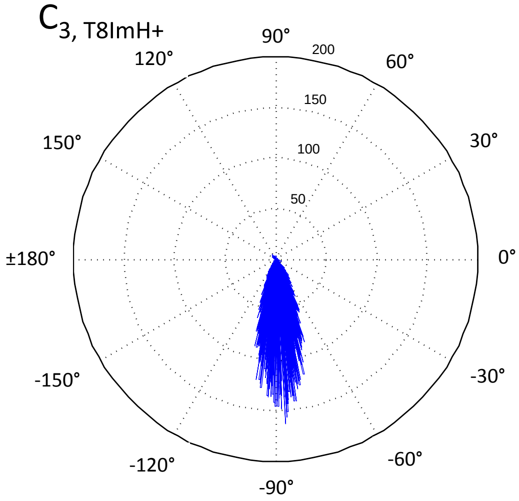

G_{26}, T8ImH+

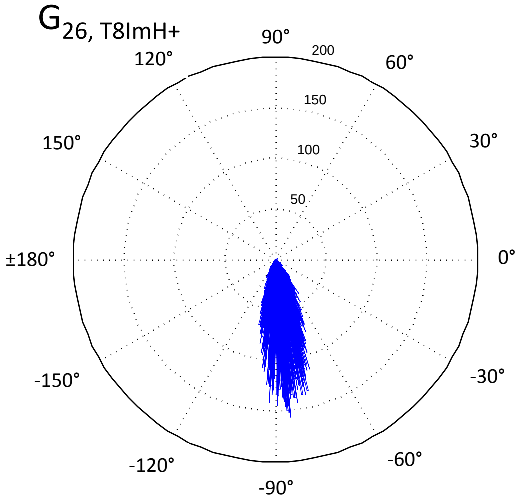

C_{4}, T8ImH+

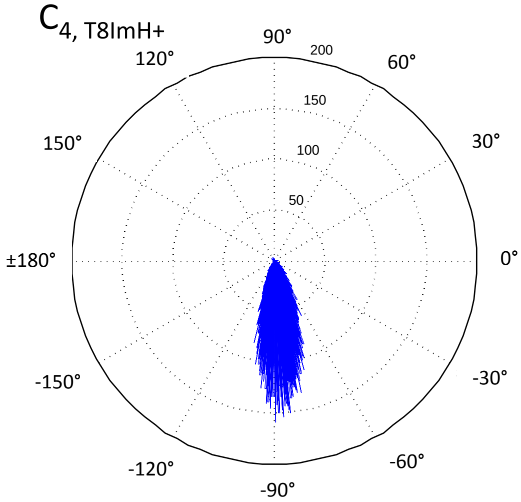

G_{25}, T8ImH+

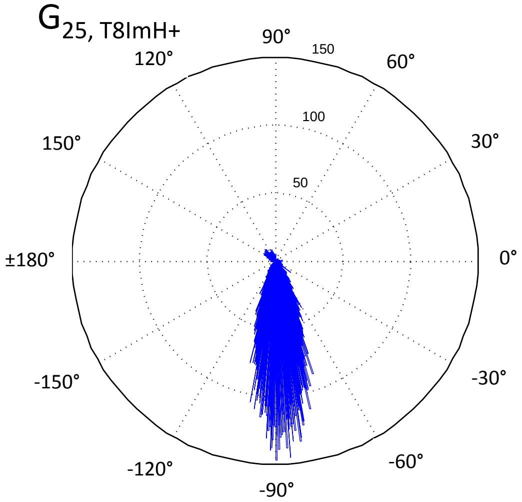

A_{5}, T8ImH+

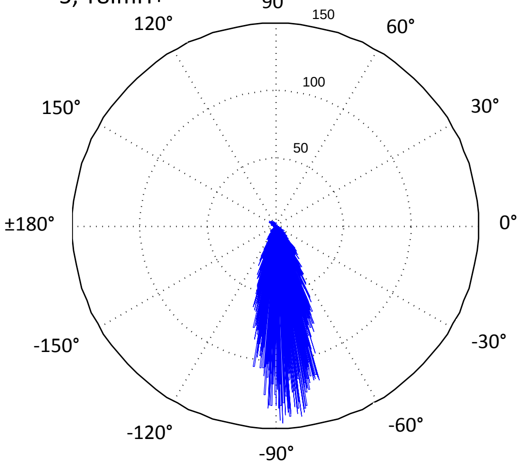

T_{24}, T8ImH+

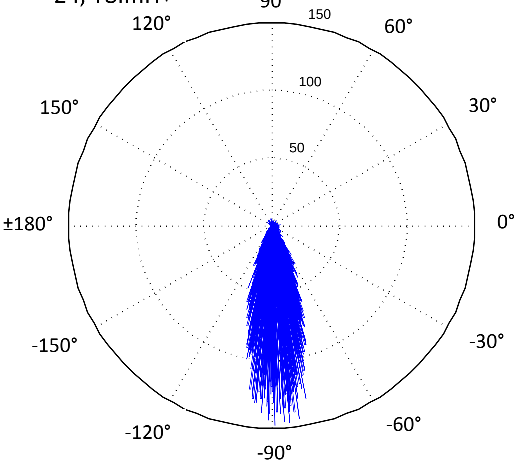

T_{6}, T8ImH+

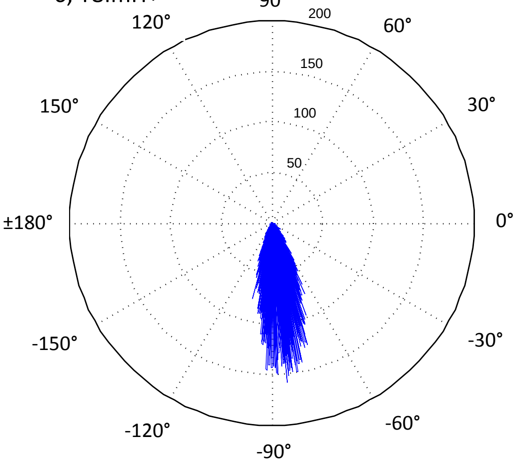

A_{23}, T8ImH+

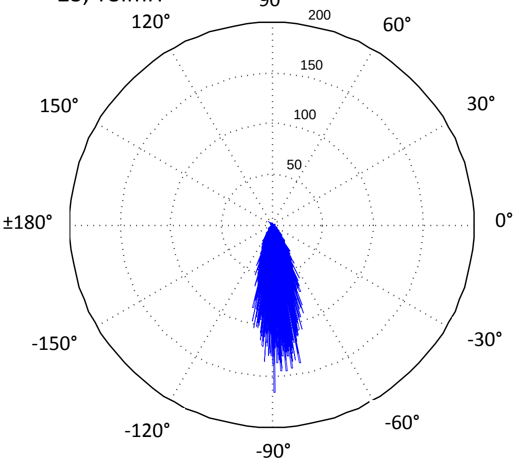

T_{7}, T8ImH+

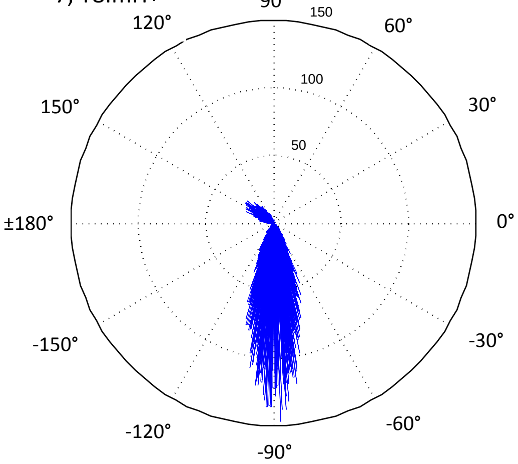

A_{22}, T8ImH+

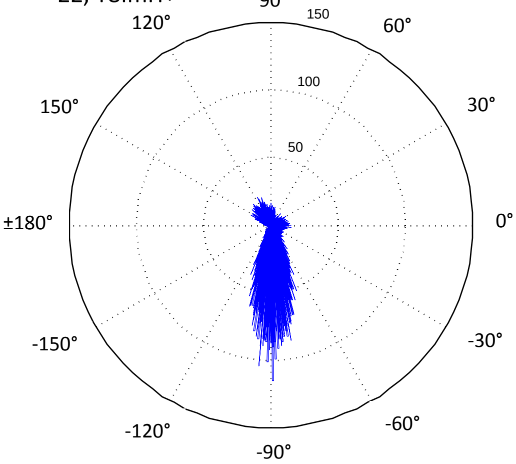

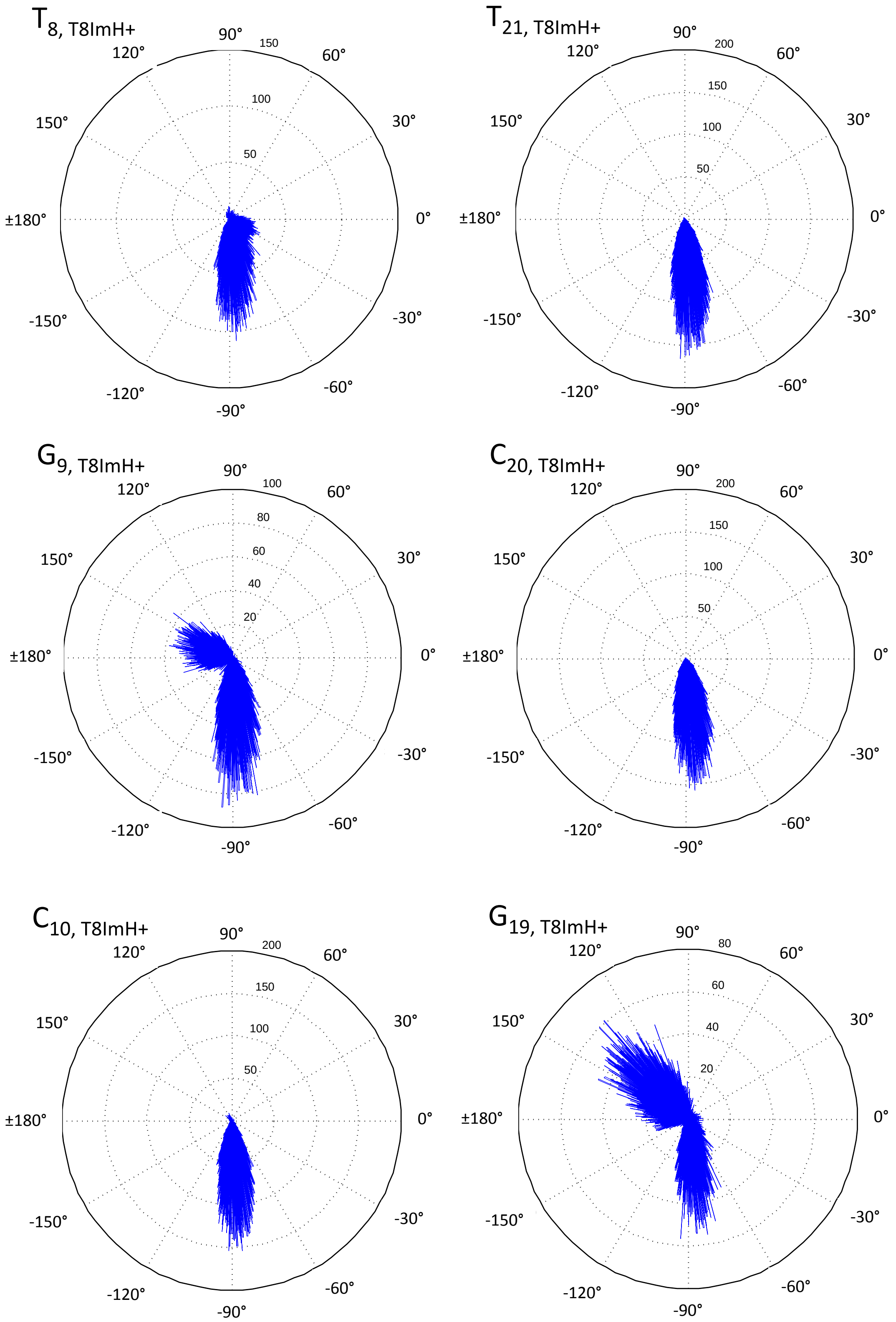

T8, T8ImH+
90°
150
100
50
120°
60°
150°
30°
±180°
0°
-150°
-30°
-120°
-60°
-90°
T21, T8ImH+
90°
200
150
100
50
120°
60°
150°
30°
±180°
0°
-150°
-30°
-120°
-60°
-90°
G9, T8ImH+
90°
100
80
60
40
20
120°
60°
150°
30°
±180°
0°
-150°
-30°
-120°
-60°
-90°
C20, T8ImH+
90°
200
150
100
50
120°
60°
150°
30°
±180°
0°
-150°
-30°
-120°
-60°
-90°
C10, T8ImH+
90°
200
150
100
50
120°
60°
150°
30°
±180°
0°
-150°
-30°
-120°
-60°
-90°
G19, T8ImH+
90°
80
60
40
20
120°
60°
150°
30°
±180°
0°
-150°
-30°
-120°
-60°
-90°

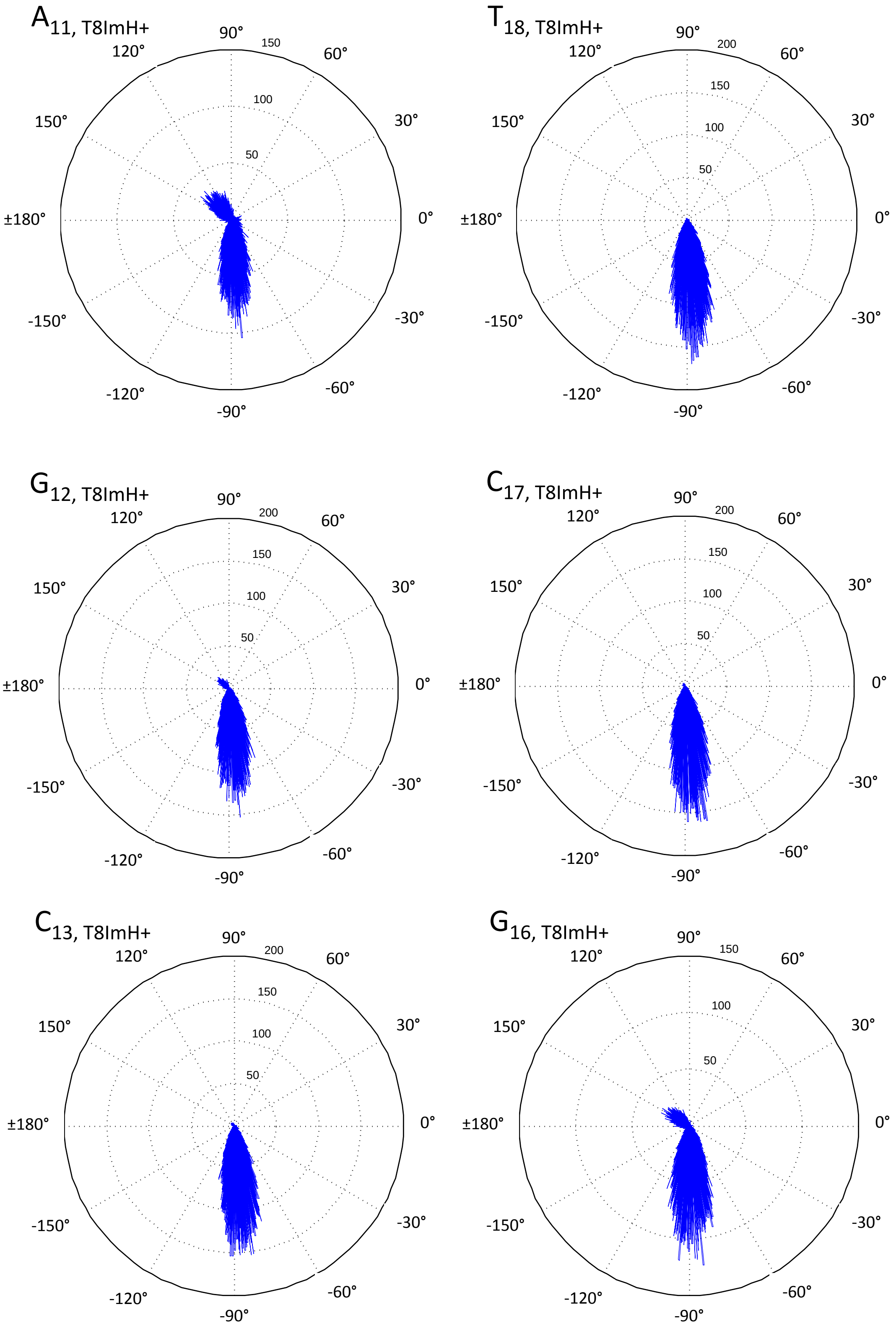

Figure S17. B_I/B_{II} torsional parameter (ε-ζ) sampling of the equilibration and production molecular dynamics trajectory of the $T_8{}^{Im}$ duplex for a total period of 50,120ns. Each data point corresponds with a snapshot every 2ps. All base pairs are shown except for the 5' and 3' ends.

A third type of analysis concerning the structural integrity of the DNA duplex can reveal whether there's any particular bias towards a certain helical morphology (e.g. A-, B-,.. duplex type). In figure S18 and S19 the classification for each dinucleotide step of the wild type and T_8^{Im} duplex are shown. In this context, it has been shown that the phosphate base $Z_p/Z_{p(h)}$ metric is a particularly effective parameter in discriminating between the three mentioned forms of DNA. In general, no unexpected observations can be made regarding both duplexes except for step 7 of the wild type: here it may be clear that there are by and large two partially overlapping but distinct clusters present. In addition, a considerable part of all the structures are located in the TA-like region of the $Z_p/Z_{p(h)}$ space. This is no longer the case for step 7 in T_8^{Im}; here almost the entire range of sampled structures is spent in the B-like DNA region. In addition, again some clustering during specific time periods is visible. The situation is more or less reversed when observing the figures concerning step 8. Again, since the T_8^{Im} duplex is quite unique in its local structure at this position, some distortion of the classic B-DNA duplex is to be expected and indeed can be confirmed when comparing step 8 among wild type and T_8^{Im}. More specifically, in T_8^{Im}, T_8 exhibits a non-negligible amount of structures in the TA-like region. Nevertheless, it is reassuring that although some distortions are noticeable, no extreme outliers of helical morphology can be observed and a more than significant amount of structures are still present in the B-like DNA space.

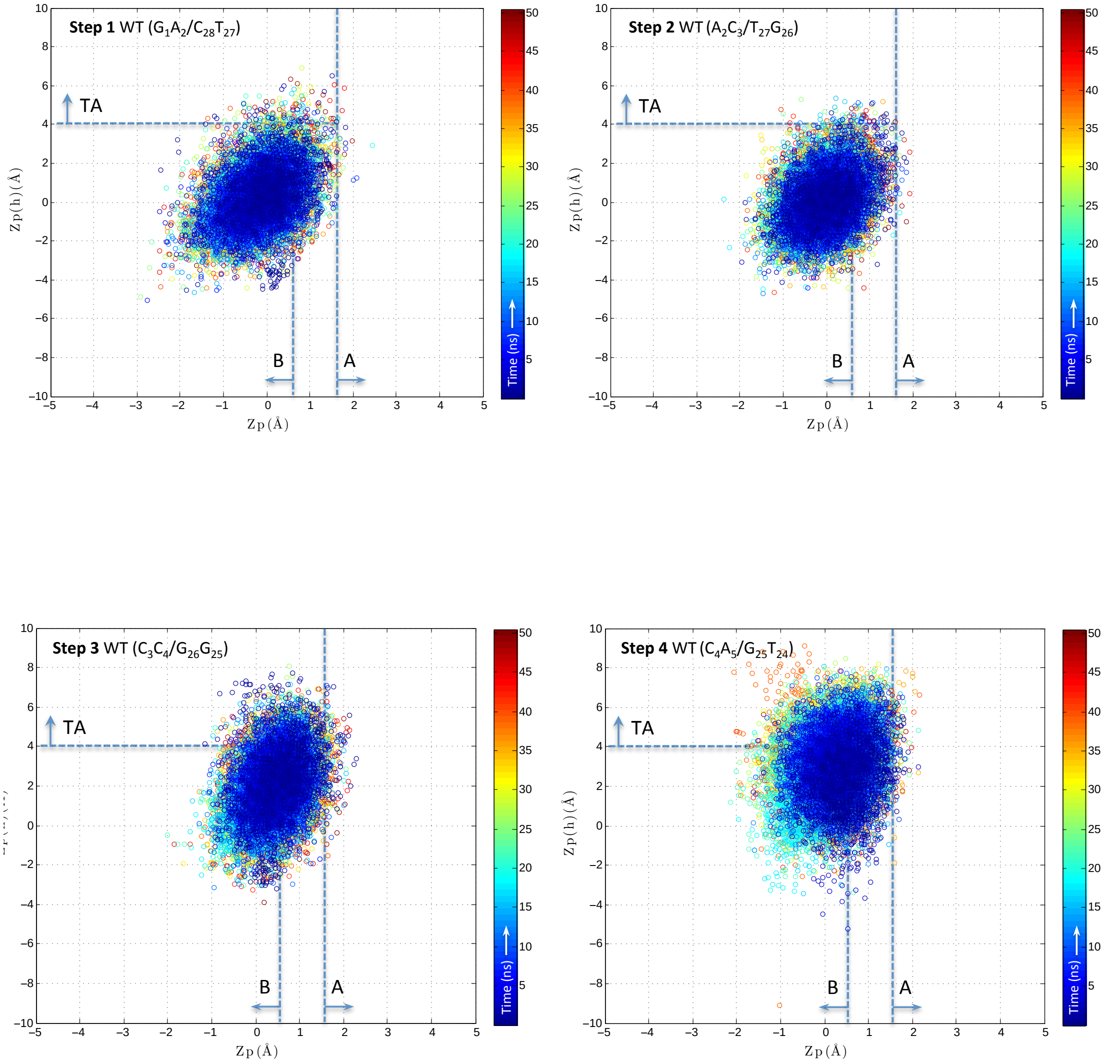

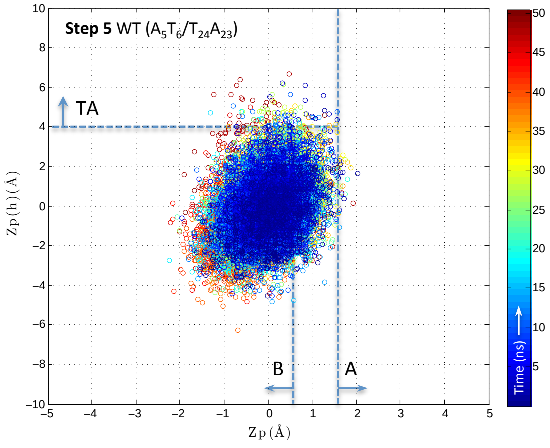
Step 5 WT (A$_5$T$_6$/T$_{24}$A$_{23}$)
TA
B
A
Zp(h)(Å)
Zp(Å)
Time (ns)

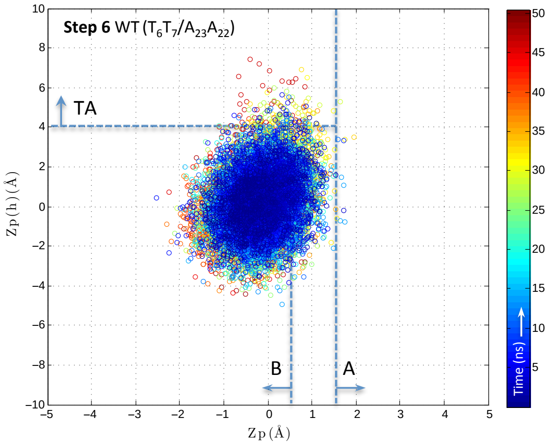
Step 6 WT (T$_6$T$_7$/A$_{23}$A$_{22}$)
TA
B
A
Zp(h)(Å)
Zp(Å)
Time (ns)

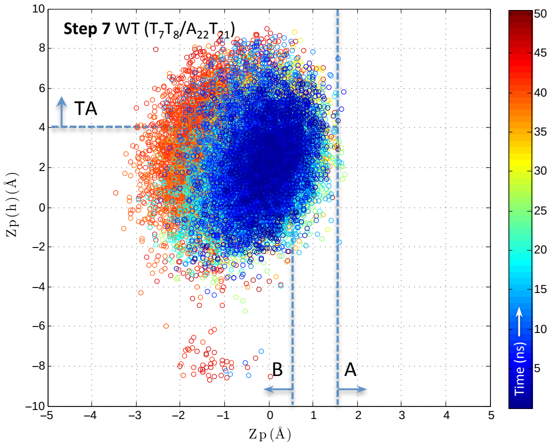
Step 7 WT (T$_7$T$_8$/A$_{22}$T$_{21}$)
TA
B
A
Zp(h)(Å)
Zp(Å)
Time (ns)

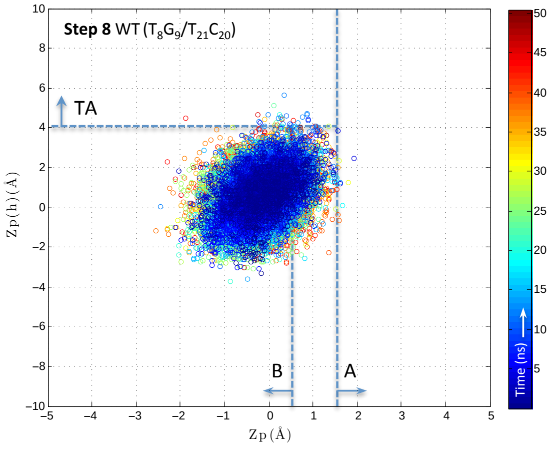
Step 8 WT (T$_8$G$_9$/T$_{21}$C$_{20}$)
TA
B
A
Zp(h)(Å)
Zp(Å)
Time (ns)

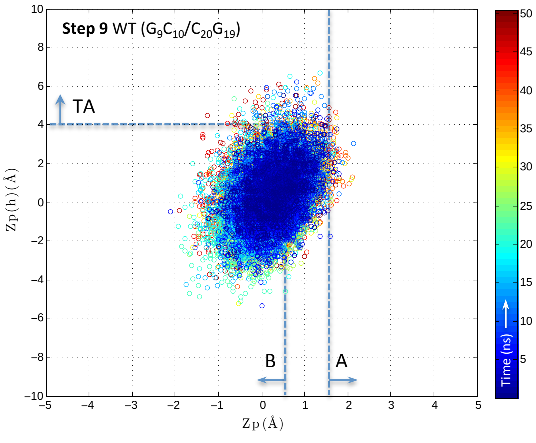
Step 9 WT (G$_9$C$_{10}$/C$_{20}$G$_{19}$)
TA
B
A
Zp(h)(Å)
Zp(Å)
Time (ns)

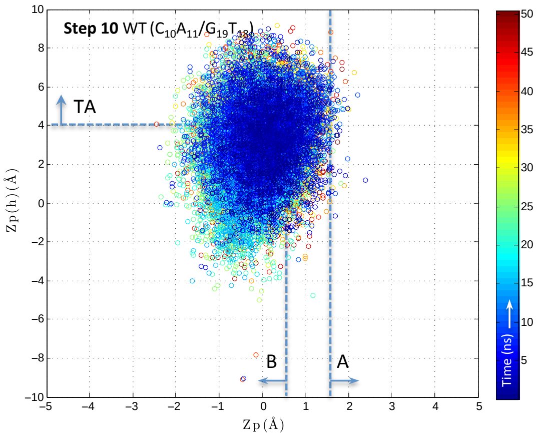
Step 10 WT (C$_{10}$A$_{11}$/G$_{19}$T$_{18}$)
TA
B
A
Zp(h)(Å)
Zp(Å)
Time (ns)

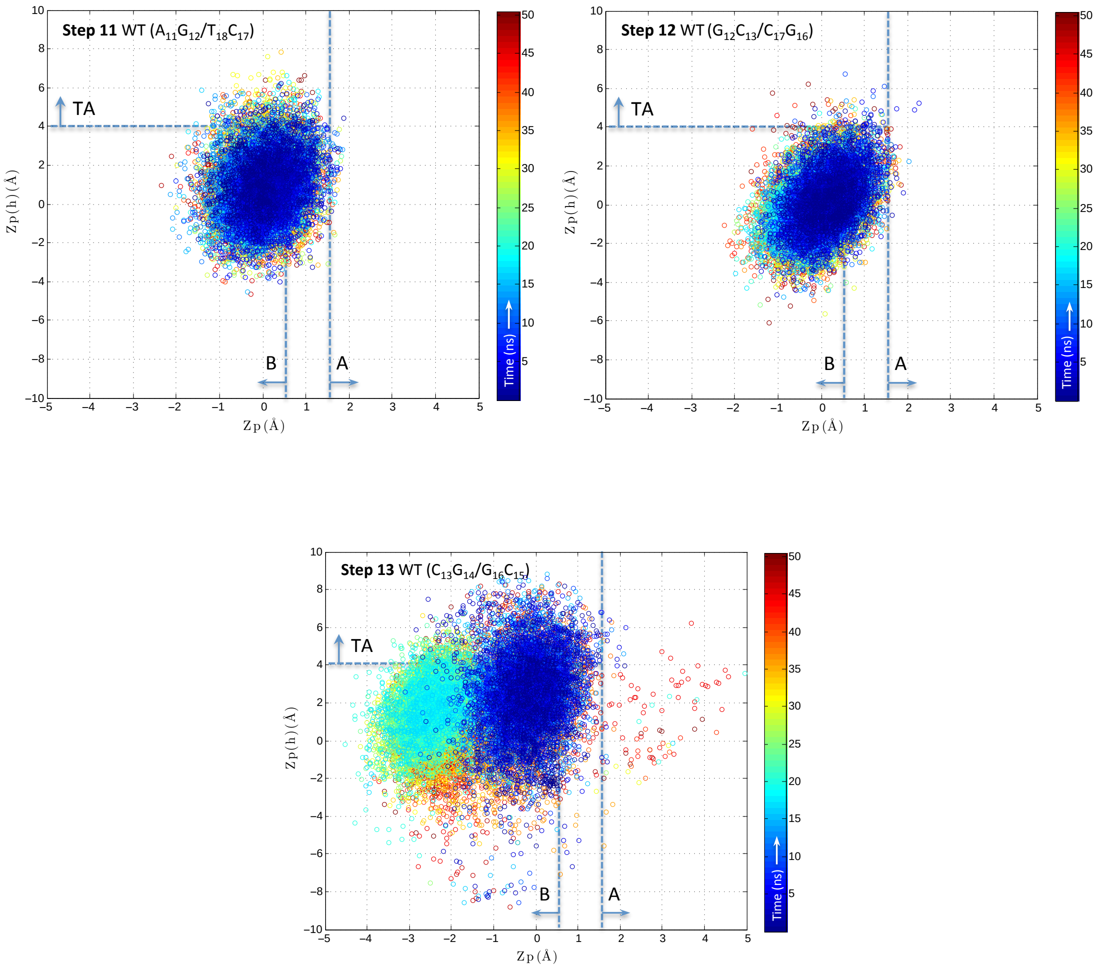

Figure S18. Zp/Zp(h) sampling of the equilibration and production molecular dynamics trajectory of the wild type duplex for a total period of 50,120ns. Each data point corresponds with a snapshot every 2ps. In each figure a dinucleotide step is classified as either A, B or TA-type DNA for every snapshot.

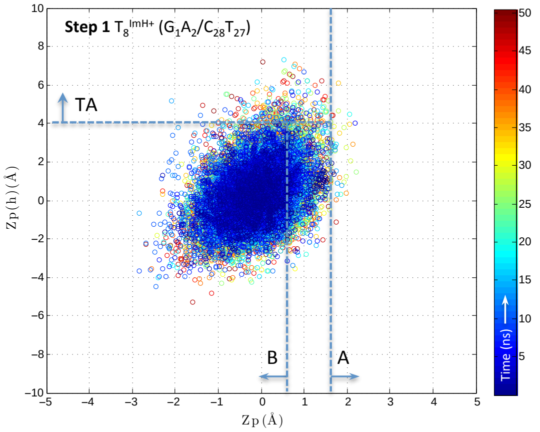
Step 1 T_8^{ImH+} ($G_1A_2/C_{28}T_{27}$)
TA
B
A
Zp(h) (Å)
Zp (Å)
Time (ns)

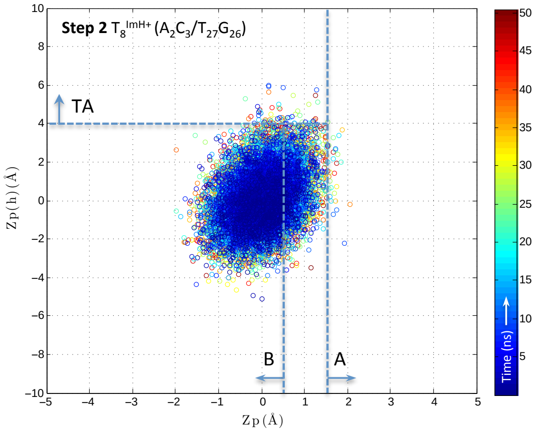
Step 2 T_8^{ImH+} ($A_2C_3/T_{27}G_{26}$)
TA
B
A
Zp(h) (Å)
Zp (Å)
Time (ns)

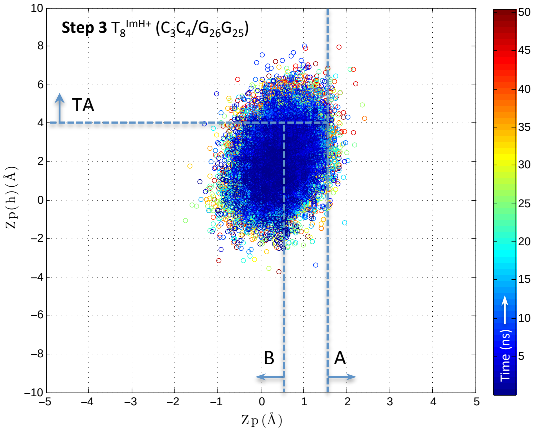
Step 3 T_8^{ImH+} ($C_3C_4/G_{26}G_{25}$)
TA
B
A
Zp(h) (Å)
Zp (Å)
Time (ns)

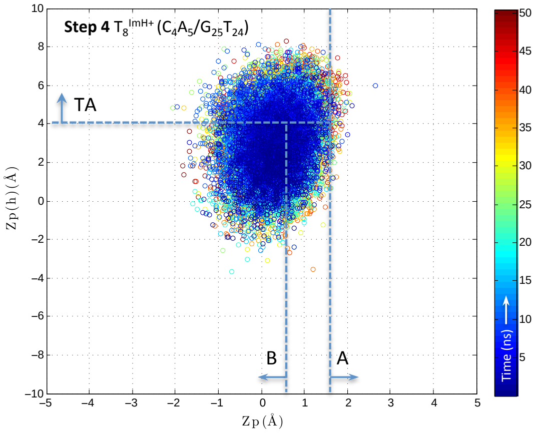
Step 4 T_8^{ImH+} ($C_4A_5/G_{25}T_{24}$)
TA
B
A
Zp(h) (Å)
Zp (Å)
Time (ns)

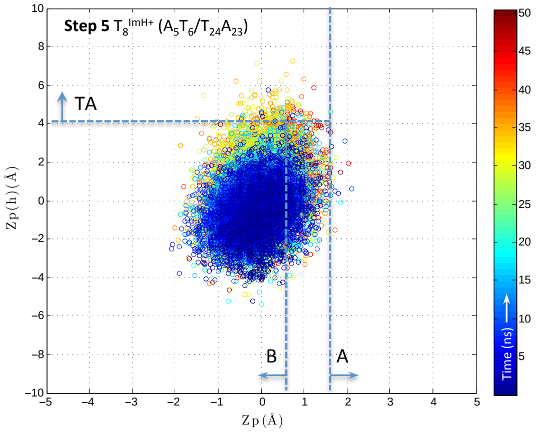
Step 5 T_8^{ImH+} ($A_5T_6/T_{24}A_{23}$)
TA
B
A
Zp(h) (Å)
Zp (Å)
Time (ns)

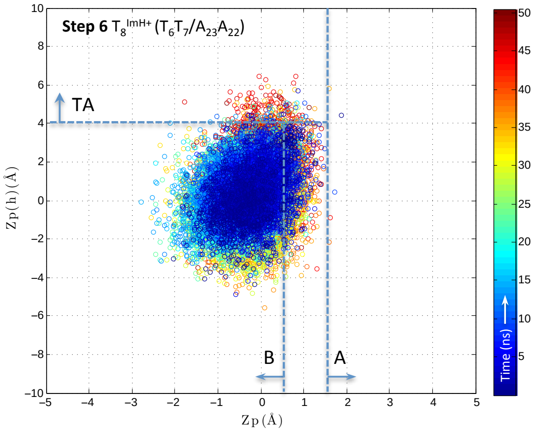
Step 6 T_8^{ImH+} ($T_6T_7/A_{23}A_{22}$)
TA
B
A
Zp(h) (Å)
Zp (Å)
Time (ns)

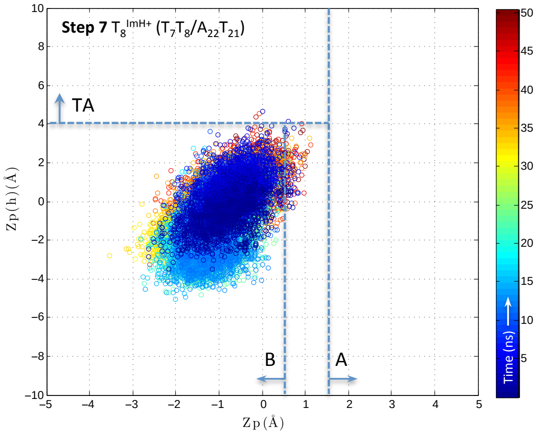
Step 7 T_8^{ImH+} ($T_7T_8/A_{22}T_{21}$)
TA
B
A
Zp(h) (Å)
Zp (Å)
Time (ns)

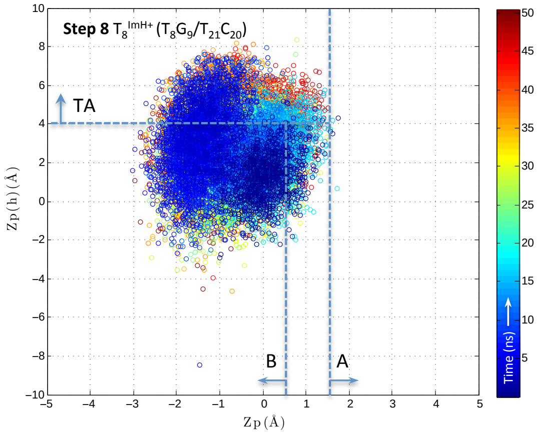
Step 8 T_8^{ImH+} ($T_8G_9/T_{21}C_{20}$)
TA
B
A
Zp(h) (Å)
Zp (Å)
Time (ns)

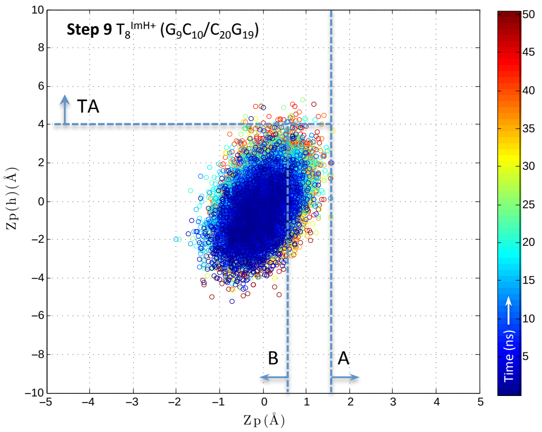
Step 9 T_8^{ImH+} ($G_9C_{10}/C_{20}G_{19}$)
TA
B
A
Zp(h) (Å)
Zp (Å)
Time (ns)

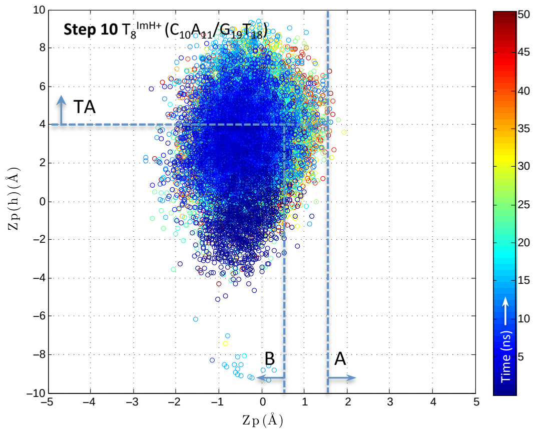
Step 10 T_8^{ImH+} ($C_{10}A_{11}/G_{19}T_{18}$)
TA
B
A
Zp(h) (Å)
Zp (Å)
Time (ns)

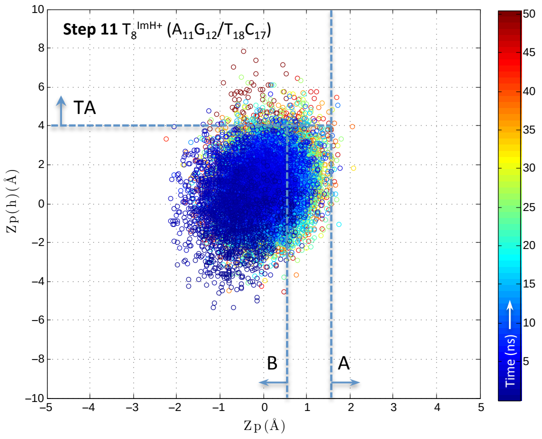
Step 11 T_8^{ImH+} ($A_{11}G_{12}/T_{18}C_{17}$)
TA
B
A
Zp(h) (Å)
Zp (Å)
Time (ns)

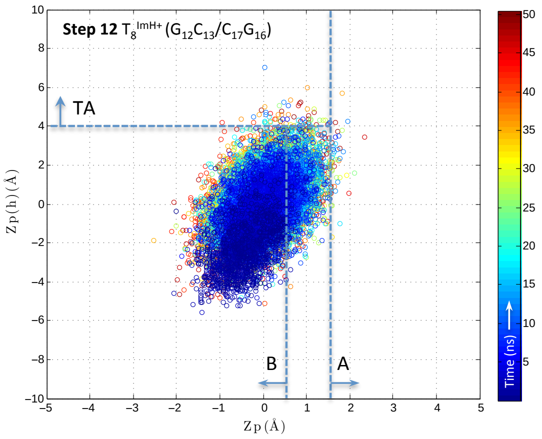
Step 12 T_8^{ImH+} ($G_{12}C_{13}/C_{17}G_{16}$)
TA
B
A
Zp(h) (Å)
Zp (Å)
Time (ns)

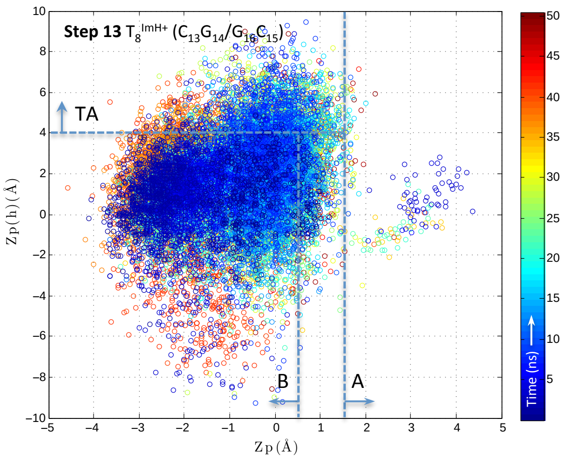

Figure S19. Zp/Zp(h) sampling of the equilibration and production molecular dynamics trajectory of the T_8^{Im} duplex for a total period of 50,120ns. Each data point corresponds with a snapshot every 2ps. In each figure a dinucleotide step is classified as either A, B or TA-type DNA for every snapshot.

Hydrogen bond persistence T_{21}^{Im} duplex

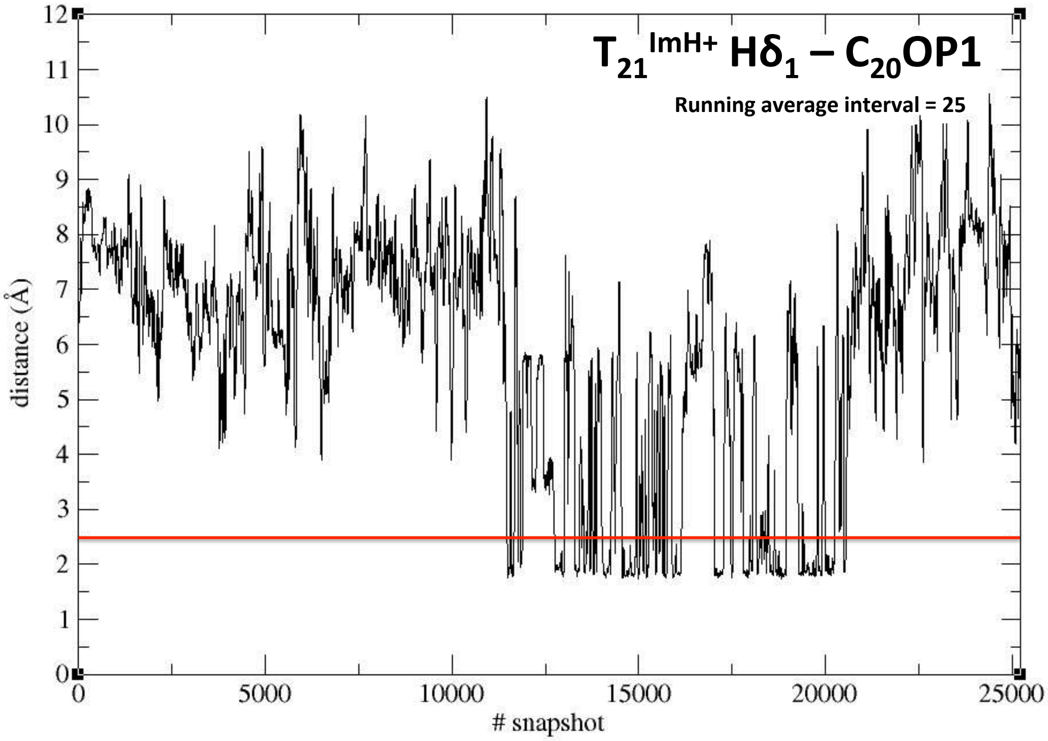

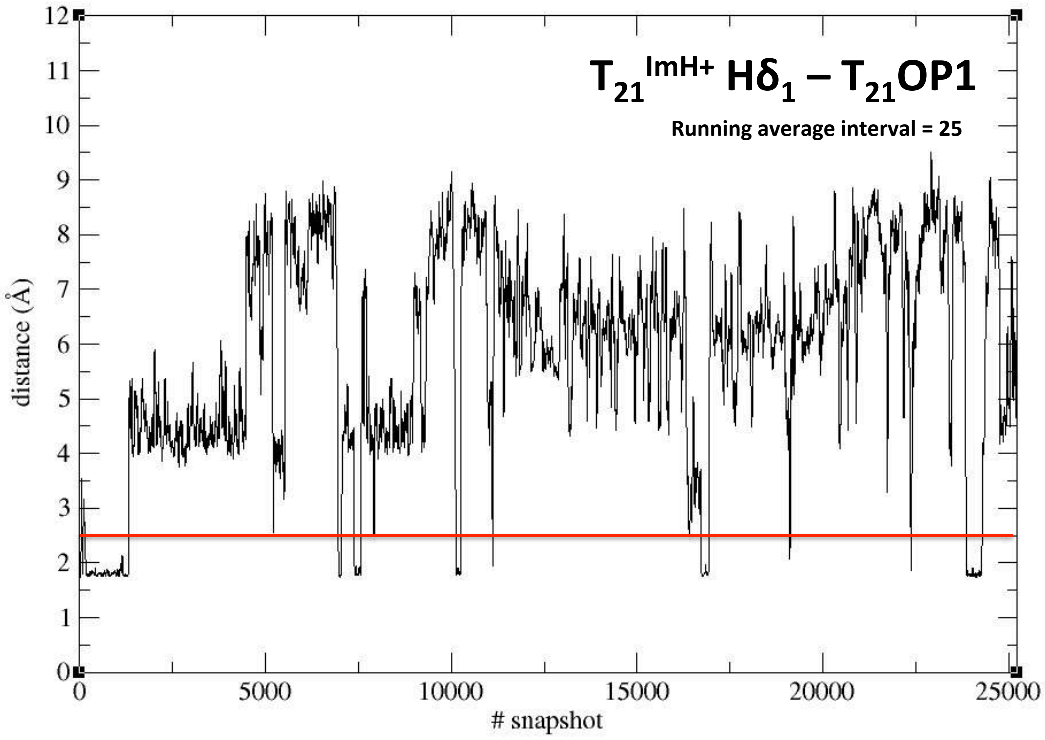

Figure S20. Hydrogen bond persistence of the two hydrogen bond interactions found for the T21Im duplex during the 50ns molecular dynamics trajectory.

Hydrogen bond persistence in the T8ImA21 versus T8Im duplex

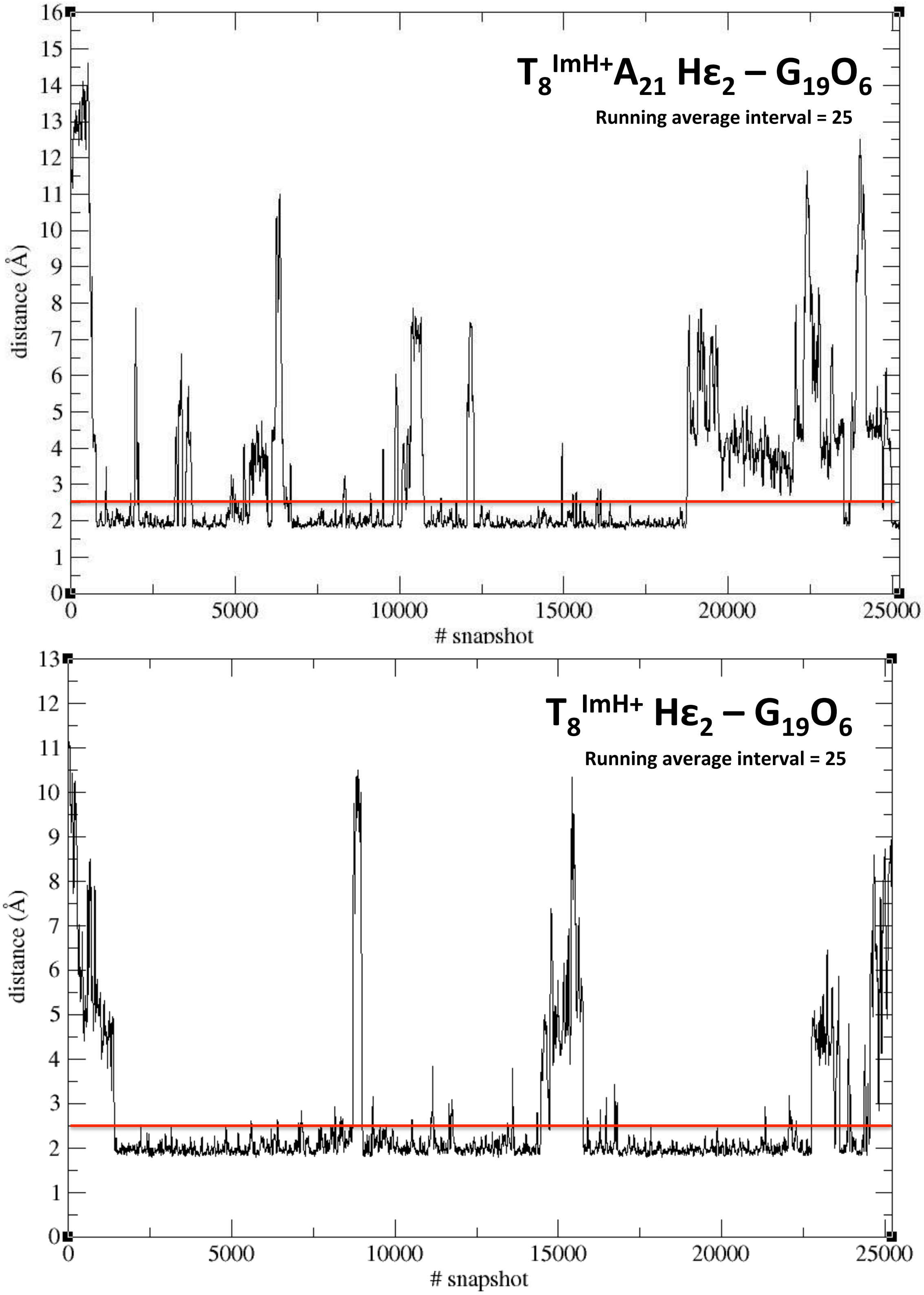

Figure S21. Comparison of the hydrogen bond persistence of the two specific hydrogen bond interactions found for the T_8Im and T_8^{Im}A21 duplex during the 50ns molecular dynamics trajectories.

Overview specific Hε1 nOe contacts

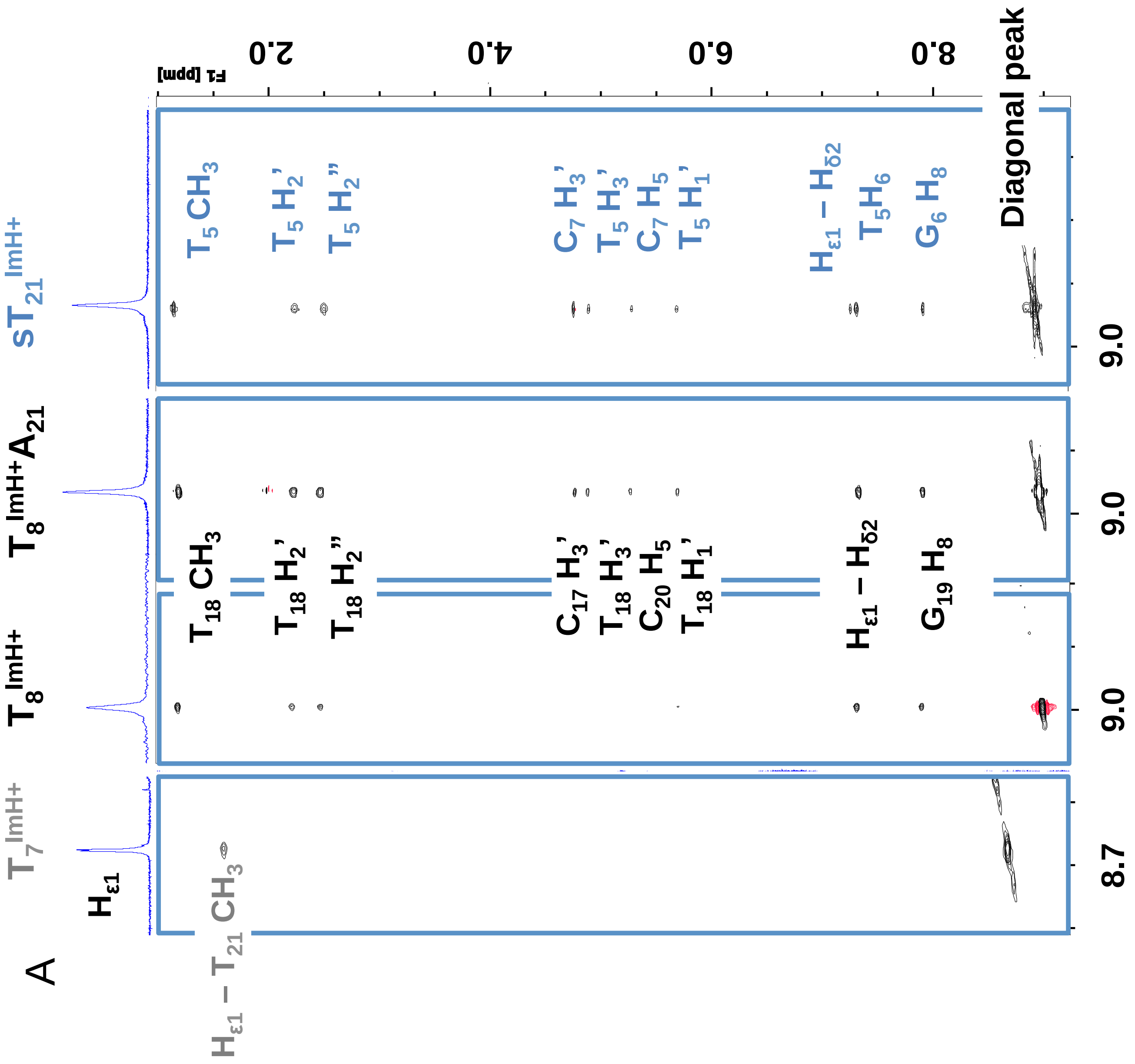

Figure S22. Overview f the specific nOe contacts of the Hε1 proton of T_7^{Im}, T_8^{Im}, $T_8^{Im}A_{21}$ and sT_{21}^{Im} to the DNA backbone (2D NOESY, 200ms, 700MHz, 25°C, D_2O).

Overview specific Hε1 – Hδ2 – Nε2-H imino nOe contacts

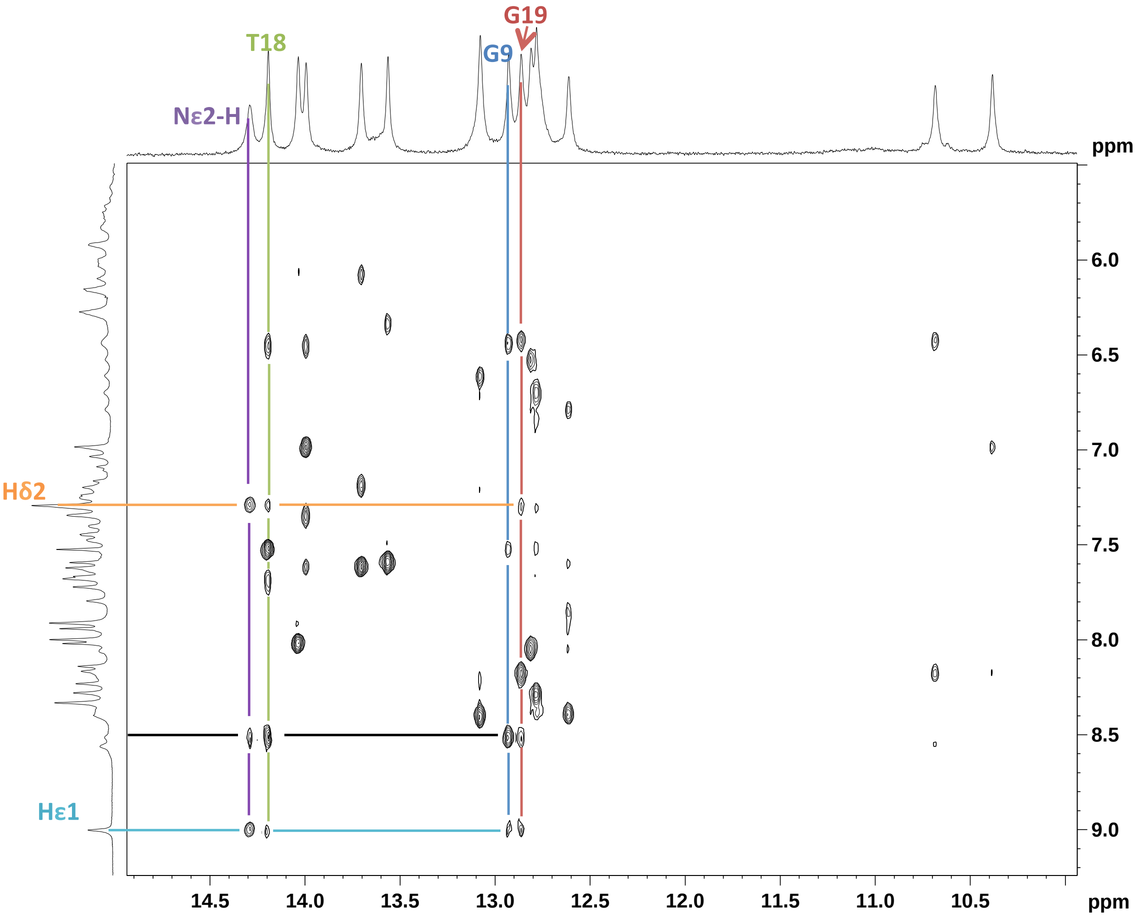

Figure S23. Overview of the specific nOe contacts of the Hε1/Hδ2/Nε2–H proton of T_8^{ImH+} to the DNA exchangeable imino and imidazole protons (2D NOESY, 80ms, 700MHz, 5°C, H_2O/D_2O, pH 5.0).

Overview ^{31}P spectra T_8^{Im} and wild type sequence

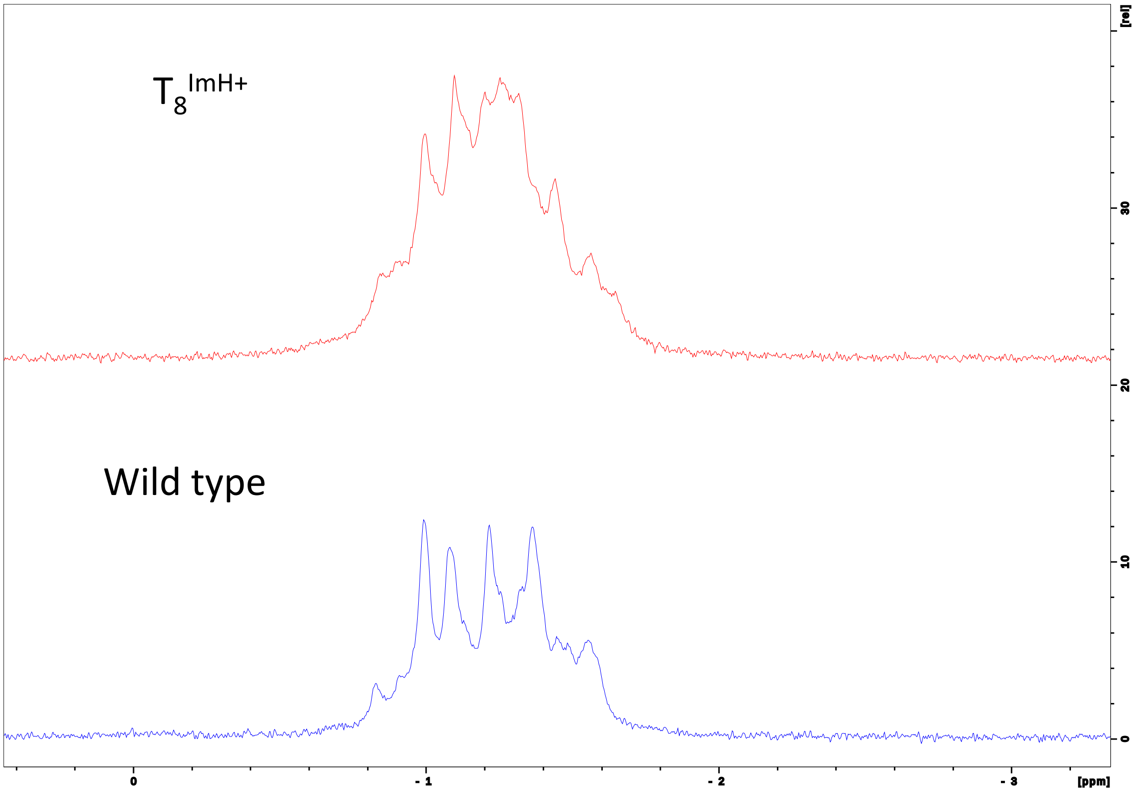

Figure S24. Overlay of the 31P spectra of both the T_8^{Im} (top) and wild type sequence (bottom, 500MHz, 298K, D_2O, pD 5.0)

In order to assess the impact of the imidazole modification and its behaviour on the phosphodiester backbone, ^{31}P spectra for both the wild type and T_8^{Im} sequence were measured at pD 5.0 and 298.15K. As can be observed in the overlay (Figure S24), at first glance the differences between both spectra are limited. In case of the wild type spectrum, the signals are all residing in a region of ± 1 to 1.5 ppm indicating all phosphodiester atoms experience a rather homogeneous chemical environment, which would be the case if the perturbation of the DNA helix is very small to negligible. This is to be expected for the wild type duplex, since the only non-standard base pair present is the T-T mismatch, known to be fairly well incorporated in otherwise ‘normal’ B-DNA duplexes. Comparing this observation to the B_I/B_{II} classification for each nucleotide in the 50ns trajectory of the wild type, it may be clear that only a small fraction of nucleotides actually show limited sampling of the B_{II} sub state, with the exception of G_{19}. These nucleotides are located either in the vicinity of the T-T mismatch or near a terminal, possible fraying base pair where, in both cases, a higher mobility of the backbone is to be expected. Hence, in both cases the perturbation of the wild type phosphodiester backbone appears to be limited.

Concerning the ^{31}P spectrum, the same observation appears to be valid for the T_8^{Im} duplex, since only a limited perturbation of the phoshpodiester signals with respect to the wild type can be observed. Considering the B_I/B_{II} torsional parameter for the T_8^{Im} system, no apparent changes in behaviour can be observed with the exception of the T_7, G_{19} and G_9 nucleotide, which indeed show a higher sampling (>30%) of the B_{II} sub state. This can be expected since these nucleotides are also the main site of interaction for the protonated imidazole modification (G_9, G_{19}) or in close vicinity to the modified T-T mismatch (T_7). Whether these two observations concerning the T_8^{Im} system contradict each other is questionable: the resonances observed in the ^{31}P spectrum are the population weighted average of the chemical shifts due to various environments in many molecules, which may or may not show an interaction of the modification and hence may or may not experience a perturbation at the phosphodiester backbone. Considering the fact that the interaction is only persistent for ±30-35% of the total simulation time, it may be very well possible that the majority of the

molecules are non-interacting and subsequently non-perturbed will dominate the ^{31}P spectrum of this system, resulting in an almost unchanged spectrum.

Extended melting temperatures table including thermodynamic data

	TIm	T-T mismatch				
**System**		WT	T_6^{ImH+}	T_7^{ImH+}	T_{21}^{ImH+}	T_8^{ImH+}
**Tm (°C)[a]**	n.r.[c]	48.6 ± 0.5	50.5 ± 0.2	50.1 ± 0.3	53.3 ± 0.3	54.6 ± 0.1
**ΔTm (°C)**	n.r.	ref[d]	1.9 ± 0.5	1.5 ± 0.6	4.7 ± 0.6	6.0 ± 0.5
**pKaH[b]**	7.17 ± 0.02	n.r.	8.13 ± 0.04	7.92 ± 0.04	7.70 ± 0.02	8.88 ± 0.05
**ΔpKaH**	ref[e]	n.r.	0.96 ± 0.04	0.75 ± 0.04	0.53 ± 0.03	1.71 ± 0.05
**ΔG (kJ/mol)[f]**	n.r.	-69.12	-71.84	-70.95	-71.33	-78.03
**ΔΔG (kJ/mol)**	n.r.	ref[d]	-2.72	-1.83	-2.21	-8.91
	**TA**		**AT**		**AT scrambled**	
**System**	T_8A_{21}	$T_8^{ImH+}A_{21}$	A_8T_{21}	$A_8T_{21}^{ImH+}$	sT_{21}	sT_{21}^{ImH+}
**Tm (°C)**	58.9 ± 0.2	64.1 ± 0.6	57.2 ± 0.2	59.3 ± 0.5	53.2 ± 0.3	59.4 ±0.2
**ΔTm (°C)**	ref	5.2 ± 0.6	ref	2.1 ± 0.5	ref	6.2 ± 0.4
**pKaH**	n.r.	8.72 ± 0.02	n.r.	7.62 ± 0.03	n.r.	9.07 ± 0.02
**ΔpKaH**	n.r.	1.55 ± 0.03	n.r.	0.45 ± 0.04	n.r.	1.9 ± 0.03
**ΔG (kJ/mol)**	-89.17	-98.16	-87.285	-88.226	-75.82	-78.47
**ΔΔG (kJ/mol)**	ref	-8.99	ref	-0.94	ref	-2.65

Table S1. Overview of melting temperatures, pKa_H, ΔG values and the differences with the respective reference systems for all duplexes studied. [a]Tm values were determined in 100 mM NaCl, 10 mM phosphate buffer, 1 μM duplex at pH = 7.0; [b]pKa_H-values for the imidazolium group were established using NMR monitoring of selected resonances, as explained in the text. [c] n.r. not relevant, since there is no melting process for the building block itself or there is no pKaH value to be determined since no imidazole functionality is present. [d]ref: reference system for the determination of the ΔTm or ΔΔG value of the corresponding imidazole modified duplex. [e] ref: reference system for the determination of the ΔpKaH values for all modified systems [f]values are calculated at 298K or 25°C and were determined using the Van ‘t Hoff extraction protocol provided by the “Thermal” software suite of the UV-VIS spectrophotometer (Varian Cary 300 Bio).
